# Systematic Review of the Literature on Dental Caries and Periodontal Disease in Socio-Economically Disadvantaged Individuals

**DOI:** 10.3390/ijerph182312360

**Published:** 2021-11-24

**Authors:** Stefano Cianetti, Chiara Valenti, Massimiliano Orso, Giuseppe Lomurno, Michele Nardone, Anna Palma Lomurno, Stefano Pagano, Guido Lombardo

**Affiliations:** 1 Department of Medicine and Surgery, Odontostomatological University Centre, University of Perugia, 06132 Perugia, Italy; stefano.cianetti@unipg.it (S.C.); chiara.valenti@studenti.unipg.it (C.V.); annalomurno@gmail.com (A.P.L.); guido.lombardo@unipg.it (G.L.); 2 Istituto Zooprofilattico Sperimentale dell’Umbria e delle Marche “Togo Rosati”, 06126 Perugia, Italy; massi.orso@hotmail.it; 3 Department of Medicine and Surgery, Oral Surgery and Ambulatory, S. Maria della Misericordia Hospital, University of Perugia, 06129 Perugia, Italy; giuseppe.lomurno@unipg.it; 4 Local Health Authority Melegnano e della Martesana, 20063 Milan, Italy; nardonemd@gmail.com

**Keywords:** dental caries, periodontitis, socio-economic vulnerability

## Abstract

Dental caries and periodontal disease represent a health problem and a social cost for the entire population, and in particular for socio-economically disadvantaged individuals who are less resistant to disease. The aim of this review is to estimate the prevalence and severity of the two dental pathologies, caries and periodontal disease, in the different classes of socio-economically disadvantaged subjects and to understand which of them are most affected. A systematic search of the literature was performed in MEDLINE (via PubMed), EMBASE and Web of Science after establishing a suitable search strategy for each database, using keywords related to socio-economically vulnerable classes and health outcomes. Socio-economically disadvantaged individuals are more susceptible to tooth decay and periodontal disease (with relative tooth loss) than non-vulnerable people. Additionally, when multiple vulnerabilities are combined in the same subject, these oral diseases worsen. There is no type of vulnerability more affected by caries and periodontitis than others, since overall they all have severe disease indices. The data from this systematic literature review might be useful for health policy makers looking to allocate more resources and services to socially disadvantaged individuals, resulting in making them more resilient to oral disease due to their social marginalization.

## 1. Introduction

The lack of equality in economic development and social progress between countries, political instability, wars and consequent migratory phenomena, ecological calamities and pandemic diseases affect the socio-economic status of populations worldwide, with ever newer and wider categories of disadvantaged groups [1]. However, socio-economically disadvantaged individuals are found in every social context, in both industrialized and developing countries [2].

One of the most common aspects of socio-economically disadvantaged people is poverty, with shared features such as low education [3], unemployment, strenuous and precarious jobs [4], uncertain housing [5], lack of health insurance [6], immigration [7] and incarceration [8]. Other conditions that cause social vulnerability are related to the ebb and flow of life itself, such as childhood, adolescence [9], old age [10] and pregnancy [11]. Finally, there are less easily classifiable but socio-economically important categories of vulnerability such as minorities due to ethnic origin [12], sexual orientation [13] or family status (e.g., single-parent families or families with separated parents) [13]. All of these types of disadvantaged individuals experience isolation and social exclusion as well as poor health and disparate healthcare access [12]. Moreover, it has been highlighted in the literature that individuals living with a low socio-economic status are more likely to have an unhealthy lifestyle through adopting bad habits such as alcohol, tobacco and drug abuse, use of illegal drugs and poor diet and personal hygiene, all of which affect one’s general and oral health [14,15,16].

Dental caries and periodontal disease are classified among the most common non-communicable diseases in the world and, in addition, the most prevalent diseases of the mouth [17]. These diseases result in reduced masticatory, swallowing and speech functions as well as pain and oral infections, leading to an increased risk of systemic diseases (e.g., heart attack, stroke) and psychological and relational disorders [18,19]. Similarly to all non-communicable systemic diseases, caries and periodontal disease are also expected to be more prevalent and severe among socio-economically disadvantaged subjects [19].

This study attempts to offer a comprehensive view of the dental health situation of socially vulnerable classes, considering them in their complexity and variety, since there is a lack of information in the literature.

The aim of this systematic review is to provide updated and exhaustive data (through the analysis of the publications of the last five years, considering that in the literature articles only focus on a single type of vulnerability) of the prevalence and severity of dental and periodontal health of socio-economically disadvantaged subjects, making a comparison with the entire population and between the different classes of vulnerable individuals.

Null hypotheses:

1. There are no differences regarding carious pathology and periodontal disease between socio-economically disadvantaged subjects and the normal population.
2. There are no differences concerning caries and periodontal disease between different categories of socio-economically disadvantaged and vulnerable individuals.
3. There are no differences in terms of caries and periodontal disease in socio-economically disadvantaged individuals who combine different types of socio-economic vulnerability.

## 2. Materials and Methods

This review is in accordance with the Preferred Reporting Items for Systematic Review and Meta-analysis (PRISMA) 2020 Statement in order to maintain a codified organization of the study [20]. In addition, the protocol of this review has been registered in the International Prospective Register of Systematic Reviews database (PROSPERO) (registration number: CRD42021251487).

The outcomes of interest were divided into primary and secondary based on the degree of relevance for this review. Primary outcomes were dental caries, periodontal disease and tooth loss (total or partial edentulism), while secondary ones were dental service attendance, oral health-related quality of life, perceived oral health and emergency treatment. Secondary outcomes will be considered only when their relationship to caries and periodontitis is clear from reading the text (e.g., not when referring to oral cancer or other oral diseases).

Considering the eligibility criteria, any type of observational study design was evaluated as suitable for inclusion in this review such as cross-sectional, case–control, prospective and retrospective cohort studies. Clinical trials were also suitable for inclusion when reporting epidemiological baseline data (useful for this review) before the start of the intervention(s). Only studies published within the last 5 years (30 November 2015 to 30 November 2020) and those investigating the prevalence and severity of dental caries and periodontal disease in adult socio-economically disadvantaged subjects (socio-economically vulnerable people) were considered. Studies written in a language other than English were excluded. Additionally, studies that did not address any of the afore mentioned outcomes or socially vulnerable people were also excluded.

A systematic search of electronic databases was performed using a combination of MeSH terms and free-text words adapted to each database to maximize database specificity. The complete search strategies are reported in Supplementary Table S1. The searched databases included MEDLINE (via PubMed), EMBASE and Web of Science. Further searches were performed in the reference lists of relevant studies, and in book chapters and literature reviews dealing with the topic of interest.

All the studies resulting from the search strategies were imported into an Endnote library and duplicates were removed. Two reviewers (GL and CV) independently assessed the records (title and abstract), selecting the studies that met the eligibility criteria. Any type of disagreement was resolved by consulting a third independent reviewer (SC). After this screening, the records selected were analyzed in their full-text version, and two other reviewers (GL and SP) independently assessed whether they should be included in the review. In case of disagreement, a third author was consulted (SC). The same two reviewers carried out the extraction of the data in a standardized data form, including authors, year of publication, study design, country, setting, characteristics of participants (vulnerability category, number, percentage of women and age), primary and/or secondary outcomes, disease index, study results and notes.

**Figure 1:**
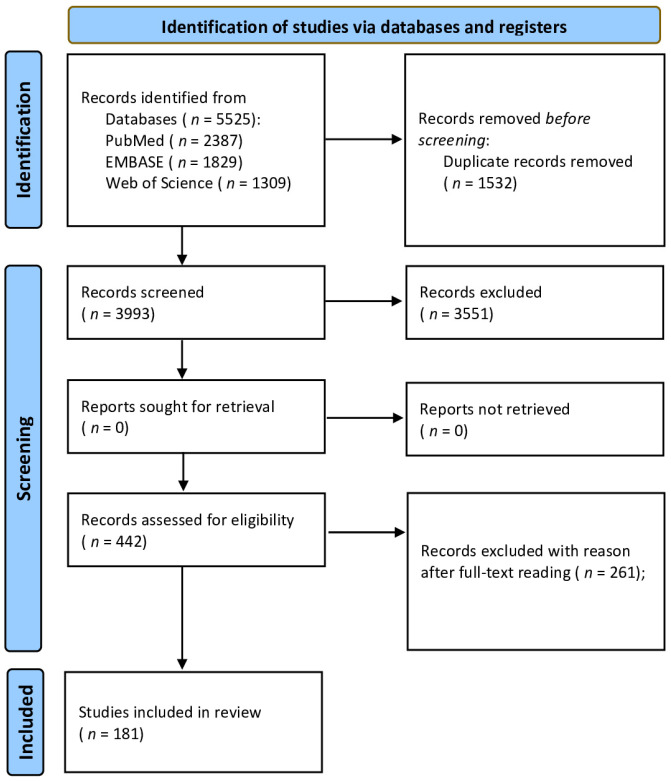
PRISMA flow diagram for systematic review.

The PRISMA flow diagram (Figure 1) was used to report the included studies according to the eligibility criteria and those excluded during the study selection process.

Quality assessment was performed independently by two reviewers (CV and GL) for each included study using the JBI’s Critical Appraisal Tool Checklist for Analytical Cross Sectional Studies, for Case-Control Studies and for Cohort Studies [21]. Any type of disagreement was resolved by consulting a third independent reviewer (SC). With this evaluation tool, the methodological quality of the included studies was evaluated, and the extent to which a study has addressed the possibility of bias in its design, conduct and analysis was determined. JBI’s Tool comprises various check-lists, each referring to a different type of study, of which we utilized three. For the cross-sectional studies, we used the 8-item checklist, considering the following: inclusion criteria definition, subjects and setting description, exposure measurements, standard criteria for measurements, confounding factor identification, strategies to deal with confounding factors, outcome measurements and statistical analysis. For cohort studies, we used the 11-item checklist, which takes into consideration the following: recruitment of population and groups, exposure similarity measures (exposed/unexposed groups), exposure measurements, confounding factor identification, strategies to deal with confounding factors, no outcome at the moment of exposure, outcome measurements, appropriate follow-up, follow-up completeness, strategies to address incomplete follow-up and statistical analysis. For case–control studies, the 10-item checklist was employed: group comparability other than presence/absence of disease, cases and controls matching, cases/controls criteria identification, exposure measurements, procedure similarity for cases and controls, confounding factor identification, strategies to deal with confounding factors, outcome assessment, appropriate exposure period and statistical analysis. The studies could then be rated as good, moderate or poor quality by answering the questions in the categories mentioned with “Yes”, “No”, “Unclear” or “Not/Applicable”.

We assessed whether the homogeneity of the studies considered was sufficient in terms of design, population characteristics and type of oral disease to allow meta-analyses. Furthermore, the results were described narratively in the main text and in the tables. Meta-analysis of the prevalence of caries and periodontal disease in disadvantaged subjects was performed using random effects models, assuming a high heterogeneity between studies, by using the Freeman–Tukey double arcsine transformation for stabilizing the variance. We also pooled the odds ratios reported in studies that compared the occurrence of caries and periodontal disease between disadvantaged subjects and a control group. Additionally, in this case, we used random effects models (inverse variance method) due to a high expected heterogeneity between the included studies. We also calculated weighted means for studies having a single group of participants, and weighted mean difference for studies also having a control group. Secondary analyses were carried out with fixed effects. For all the meta-analyses, summary estimates along with 95% confidence intervals (95% CIs) were reported. Heterogeneity among studies was assessed using I² statistics. We investigated possible causes of heterogeneity through subgroup analyses, comparing participants in institutional vs. non-institutional settings. The risk of publication bias in studies reporting odds ratios was not assessed due to an insufficient number of studies (i.e., <10) included in each meta-analysis. For all the statistical analyses (see Supplementary Figure S1), the software STATA 13/SE was used.

## 3. Results

Of the 3993 records obtained from the analysis of the databases, adopting the search strategy described in the Materials and Methods section, 442 were selected for reading the full text, 181 of which were included as they met the inclusion criteria and satisfied the quality assessment (see Figure 1 and Supplementary Table S2). A total of 168 studies were cross-sectional, four were case–control studies and nine were cohort studies.

### 3.1. Prevalence of Caries

**Figure 2:**
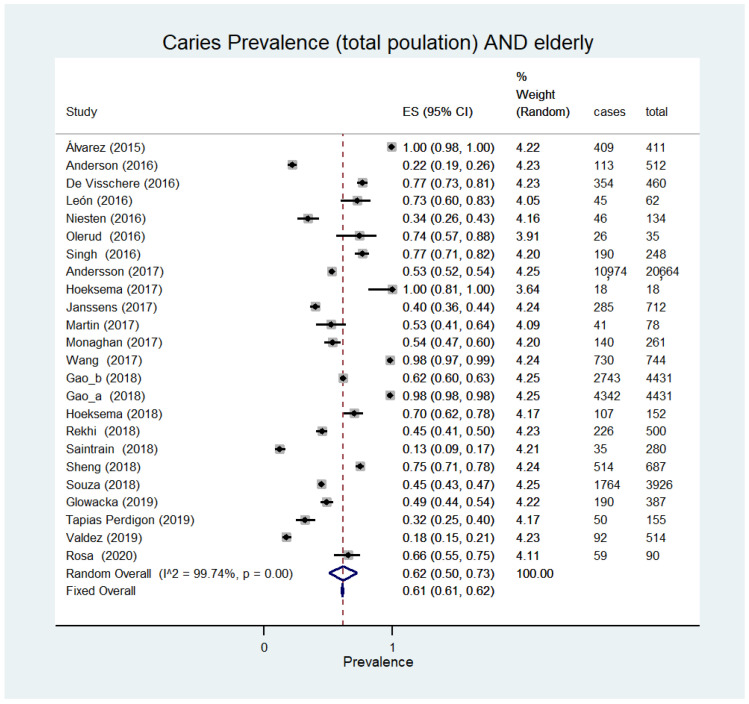
Caries prevalence (total population) AND elderly.

The prevalence of caries was expressed in the six types of disadvantaged subjects, starting in the description with those most affected, such as prisoners with 77% ([95% CI 66–85%]; 803 participants, three studies, I^2^ = 90%) [22,23,24], elderly people with 62% ([95% CI 50–73%]; 38,133 participants, 24 studies, I^2^ = 99.7%) [25,26,27,28,29,30,31,32,33,34,35,36,37,38,39,40,41,42,43,44,45,46,47,48] (Figure 2), alcohol and drug abusers with 60% ([95% CI 56–64]; 592 participants, two studies, I^2^ = not assessable) [49,50], immigrants with 65% ([95% CI 18–99%]; 878 participants, three studies, I^2^ = 99.1%) [39,51,52], followed by pregnant women with 29% ([95% CI 8–56%]; 506 participants, three studies, I^2^ = 97.4%) [53,54,55]. All groups showed high scores of prevalence.

When disadvantaged subjects were compared with the general population, they had a higher level of caries. Low-income subjects, indeed, have a double probability of suffering from caries (OR 2.2 [95% CI 1.7–2.7], *p <* 0.05; 6524 participants, two studies) [56,57], while subjects with a low level of education showed a greater than one and a half times probability (OR 1.6 [95% CI 1.42–1.9], *p <* 0.05; 5653 participants) [57], with a similar odds ratio to immigrants (OR 1.66 [95% CI 1.29–2.13], *p <* 0.05; 3738 participants) [58] and individuals living in rural areas (OR 1.6 [95% CI 1.2–4.3], *p* = 0.01; 280 participants) [59].

### 3.2. Caries Experience (DMFT)

Caries experience (DMFT) was calculated in six types of disadvantaged individuals, of which the elderly were those with the highest DMFT 18.7 SD 2.4 ([95% CI 13.5–24]; 1160 participants, 17 studies) [25,28,30,31,34,40,41,44,48,60,61,62,63,64,65,66], followed by homeless subjects with DMFT 17.3 SD 0.4 ([95% CI 12.6–22.1]; 100 participants, two studies) [67,68], immigrants with 14.9 SD 0.9 ([95% CI 9.6–18.7]; 843 participants, two studies) [51,52], alcohol and drug abusers with 12.9 SD 0.8 ([95% CI 11.2–14.6]; 1891 participants, nine studies) [49,50,69,70,71,72,73,74,75], low-income people with9.9 SD 0.5 ([95% CI 3–16.7];3043 participants, two studies) [60,76], low-education people with 9.8 SD0.03 ([95% CI 9.4–10.2]; 3043 participants, two studies) [60,76] and prisoners with 8.9 SD 0.8 ([95% CI 6.3–10.4]; 802 participants, three studies) [22,23,24]. Lower DMFT was found in sex workers with 2.3 (249 participants) [77] and in subjects who work without any specific qualification with 2.9 (510 participants) [78].

In all three types of disadvantaged individuals in whom caries experience was compared with the general population, DMFT was higher in socially vulnerable people. Specifically, a greater probability of having a higher DMFT index was found in low-education people (OR 1.40 [95% CI 1.29–1.52], *p <* 0.05; 6051 participants) [57], in low-income people (OR 3.7 [95% CI 2.8–4.6], *p <* 0.05; 1695 participants, two studies, I^2^ = 78%) [56,76] and in professionals who sustain an excessive hourly workload (>40 h of overtime) (OR 2.56 [95% CI 1.23–5.33] *p* = 0.012 to OR 3.01 [95% CI 1.13–7.97] *p* = 0.027; 950 participants) [79,80]. Moreover, institutionalized elderly people (resident in care homes) showed a higher caries experience than non-institutionalized elderly people with a DMFT 25.4 SD 2.1 ([95% CI 19.9–30.9]; 2608 participants, six studies) versus 16.1 SD 2.1 ([95% CI 11.2–21]; 8276 participants, nine studies) [29,31,34,40,41,44,48,60,61,63,64,81,82]. This difference between the two was essentially due to an increased number of teeth lost because of caries, with MT 21.4 SD 2.9 ([95% CI 12.2–30.6]; 2123 participants, seven studies) in institutionalized elderly individuals versus 9.7 SD 0.5 ([95% CI 8.4–11]; 7641 participants, four studies) in non-institutionalized ones [29,31,34,40,41,48,62,63,64,66,81].

### 3.3. Gingivitis

**Figure 3:**
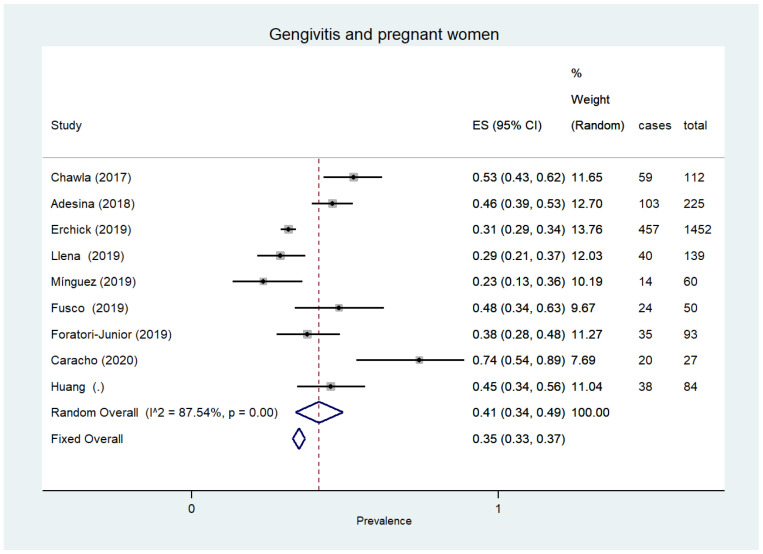
Gingivitis and pregnant women.

Gingivitis was noted in six different types of disadvantaged individuals, two of which showed the highest prevalence, such as alcohol and drug abusers with 82% ([95% CI 43–100%]; 357 patients, three studies, I^2^ 98.8%) [71,72,75] and prisoners with 63% ([95% CI 54–72%]; 802 patients, three studies) [22,23,24]. High prevalence values were also found in pregnant women with 41% ([95% CI 34–49%]; 2242 participants, nine studies) [53,54,55,83,84,85,86,87,88] (Figure 3) and the elderly with 41% ([95% CI 29–52%];11,048 participants, 15 studies) [26,28,35,39,43,47,65,66,81,89,90,91,92,93,94]. Lower prevalence values were found in subjects with low levels of education (19% [95% CI 18–19%]; 75,095 participants, two studies, I^2^ = not assessable) [95,96] and low income (19% [95% CI 18–19]; 54,593 participants, two studies, I^2^ = 0.00) [93,94] and in immigrants (15% [95% CI 12–18%]; 746 participants, two studies, I^2^ = 0.00) [39,51].

When a comparison was made, disadvantaged people showed a higher prevalence of gingivitis than non-disadvantaged people. In particular, gingivitis was more prevalent in people with low education than in individuals with higher education (OR 2.6 [95% CI 2.5–2.7%], *p <* 0.05; 75,095 participants, two studies, I^2^ = 99.1%) [95,96], in low-income people (18.8% [n. = 5615/75,095]) versus higher-income people (7, 5% [n. = 5615/75,095]) [93,94] and in drug addicts versus the general population (39.6% vs. 28.4%, *p <* 0.001; 200 participants) [75].

### 3.4. Periodontitis

**Figure 4:**
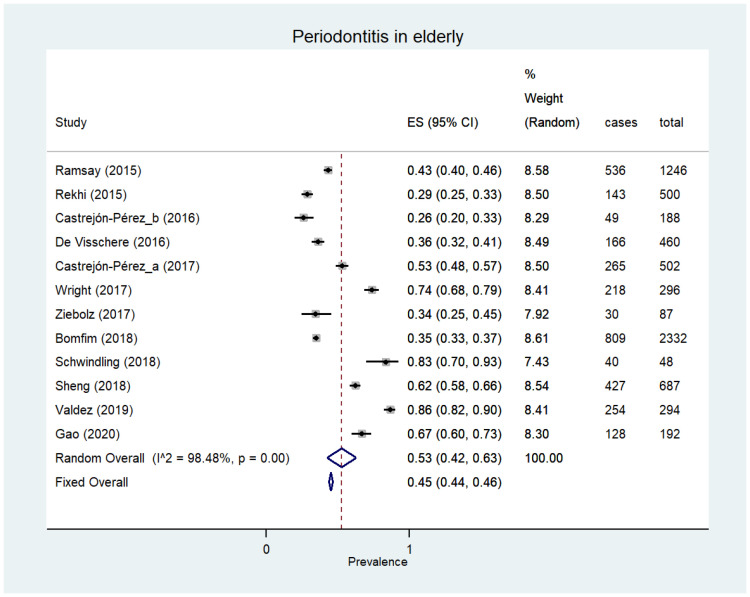
Periodontitis in the elderly.

In the included studies, the presence of periodontitis was expressed by the Community Periodontal Index (CPI 3 or 4) or with at least one gingival site with ≥4 mm of periodontal pocket depth (PPD) or with ≥4 mm loss of clinical attachment (CAL). The prevalence of periodontitis has been reported in seven types of vulnerable individuals. Considering the number of published studies, most of them (12 studies) were focused on the elderly, who showed a prevalence value of 53% ([95% CI 42–63]; 6832 participants, 12 studies, I^2^ = 98.5%) [28,43,47,66,81,89,90,91,92,94,97,98] (Figure 4). Five studies described the prevalence of this disease in pregnant women with a value of 34% ([95% CI 13–58%]; 1757 participants, five studies, I^2^ = 97.6%) [54,83,84,87,88]. Moreover, three studies evaluated immigrants with a prevalence score of 40% ([95% CI 25–56%]; 1625 participants, three studies, I^2^ = 91.4) [39,52,99] and two studies involved subjects having a low level of education with a score of 56% ([95% CI 54–59%]; 1496 participants, two studies, I^2^ = not assessable) [95,100]. In the remaining groups with vulnerabilities, the prevalence data were described through single studies evaluating unskilled workers (51.3% [n. = 261/510]) [78], drug abusers (43% [n. = 46/106]) [101] and low-income subjects (31.0% [n. = 690/2149]) [102].

In the comparative studies, periodontitis was more prevalent and/or severe in socially disadvantaged subjects than in the general population. This comparison described five different types of vulnerable individuals, as follows. In elderly subjects (≥75 years) compared to young ones (25–44 years), there was a higher probability of suffering from periodontal disease (OR 1.68 [1.30–2.17], *p <* 0.001; 1693 participants) [95]. Likewise, low level education individuals showed an increased probability of having this oral disease than more educated people (OR 2.5 [95% CI 2.2–2.8]; 6036 participants, two studies, I^2^ = 91.6) [103]. Conversely, greater education was a protective element, reducing the probability of suffering from moderate–severe (OR 0.25 [0.17–0.38] *p <* 0.01) or severe (OR 0.29 [95% CI 0.17–0.50], *p <* 0.01) periodontitis, as shown in a study of 8886 participants [104]. Low-income individuals, when compared to those with a more stable or higher income, showed a greater prevalence of periodontitis (31.0% [n. = 690/2149] vs. 23.2% [n. = 516/2216] *p <* 0.001) [100] or a greater probability of being affected by this oral disease (OR 1.35 [1.15–1.58], *p <* 0.05; 17,583 participants) [102]. In immigrants without citizenship of their host country, compared to those who obtained it, an increased probability of suffering from periodontitis was demonstrated (OR 1.95 [1.42–2.67], *p <* 0.05; 3738 participants) [58]. Subjects who were addicted to substances such as crack and/or cocaine were found to have a triple probability of suffering from periodontitis (OR 3.44 [1.51–7.86], *p <* 0.01; 106 participants) and an increased mean periodontal pocket value (PPD 2.84 SD 0.76 vs. 2.55 SD 0.73 mm, *p* = 0.04; 106 participants) [101].

### 3.5. Complete Edentulism

The prevalence of edentulism was reported in four types of vulnerable individuals. In the elderly, edentulism was found to affect almost a third of the population of participants (32% [95% CI 28–37]; 116,284 participants, 56 studies, I^2^ = 99.6%) [26,27,28,31,32,33,34,35,36,37,38,39,42,44,46,47,60,63,66,81,90,91,92,95,105,106,107,108,109,110,111,112,113,114,115,116,117,118,119,120,121,122,123,124,125,126,127,128,129,130,131,132,133,134,135,136], while in subjects with a low education level the prevalence was 17% ([95%CI17–31]; 2519 participants, three studies, I^2^ = 97.9%) [56,137,138] and 3% in drug abusers ([95% CI 2–5%]; 666 participants, three studies, I^2^ = 0.00) [50,72]. Finally, in low-income people, although based on the data expressed in a single study, the edentulous showed a prevalence of 22.6% (n. = 152/672) [137].

When a comparison was made, socially vulnerable individuals in all studies were more affected by edentulism than non-vulnerable ones, as described in low-income subjects (22.6% [n. = 152/672] vs. 3.5 [68/1934], *p <* 0.001) [137], low-education subjects (OR 6 [95% 3.3–10.7], *p* = 0.005; 6078 participants, three studies, I^2^ = 80.8%) [56,137,138], drug abusers (4.58% vs. 1.96%, *p <* 0.001; 517 participants) [50] and in black subjects (ethnic minority) (9.7% [n. = 228/2352] vs. 4.3% [n. = 335/7630], *p <* 0.001) [137].Similarly, the elderly (≥65 years), when compared to younger individuals (50–64 years), were more than twice as likely to be edentulous ((OR 2.15), *p <* 0.001; 15,473 participants) [139]. Moreover, institutionalized elders were found to have a higher edentulous prevalence (36% [95% CI 29–44]; 10,998 participants, 17 studies) [28,34,37,38,60,63,81,111,113,118,120,123,125,130,132,135] than non-institutionalized ones (25% [95% CI 21–30]; 89,115 participants, 31 studies), with a statistically significative difference (*p <* 0.05) [26,27,31,35,36,42,44,46,47,90,91,92,97,105,106,107,108,109,112,113,116,119,121,122,124,127,128,129,131,133].

### 3.6. Partial Tooth Loss (Partial Edentulism)

In nine categories of socially disadvantaged subjects, data on tooth loss outcome were expressed. All the studies agree on a greater tooth loss among socially disadvantaged subjects compared to the general population. The results of this outcome have been described for single types of vulnerable populations.

#### 3.6.1. The Elderly

More than half of the elderly showed a non-functional dentition for chewing (≤20 remaining teeth) (56% [95% CI 50–62]; 103,770 participants, 26 studies, I^2^ = 99.7%) [28,35,42,46,60,66,92,96,105,106,111,112,117,118,119,130,131,132,135,140,141,142,143,144,145,146] and more than a third of them a dentition with severe functional impairment (<10 remaining teeth) (36% [95% CI 29–43]; 42,964 participants, eight studies, I^2^ = 99.5) [96,105,117,118,142,143,145,146].

#### 3.6.2. Low-Education People

In terms of prevalence, low-education individuals, when compared to the reference group, showed an increased risk of tooth loss (OR 4.9 [95% CI 0.7–33.9], *p* = 0.083; 11,565 participants, two studies, I^2^ = 61.2%) [147,148] or had non-functional dentition (≤20 remaining teeth) (OR 2 [95% CI 0.97–4.1], *p* = 0.076; 5814 participants, two studies, I^2^ = 61.2%) [57,149]. Furthermore, they are more likely to have fewer teeth than the population median number (59.2%, n. = 734/1289 vs. 42.7%, n. = 216/538, *p <* 0.001) [102]. On the contrary, a high degree of education increases the prevalence of subjects without tooth loss (51.9% [n. = 809/1559] vs. 60.6% [n. = 1064/1753] *p <* 0.001; 6710 participants) [150]. Considering the number of residual teeth as an outcome, low income was found to be a risk factor of tooth loss. This concept was expressed as the average number of teeth per capita (23.4 SD2.6 vs. 26.7 SD 1.1, *p <* 0.01; 36,506 participants) [151,152], as the average difference in number of teeth (MD −0.279 [−3.48 to −2.10] *p <* 0.05; 5084 participants) [153] and, in addiction, as the probability of having a reduced number of teeth in terms of mean ratio (MR 0.93 [0.93–0.94], *p <* 0.001; 9564 participants) [154] or risk ratio (RR0.79 [95% CI 0.75–0.83], *p <* 0.05) [155]. The prevalence of severe tooth loss (<10 remaining teeth) also appeared to be conditioned by a low level of education, with similar scores in both men (OR 2.71 [95% CI 2.27–3.24], *p <* 0.001) and women (OR 3.00 [95% CI 2.31–3.90], *p <* 0.001) as reported in a study involving 34,975 participants [156].

#### 3.6.3. Low-Income People

In terms of prevalence, the low income compared to the reference population was more likely to have both non-functional dentition (≤20 remaining teeth) (OR 3.9 [95% CI 3.2–4.8], *p <* 0.05; 5440 participants, two studies, I^2^ = not assessable) [148,157] and a higher percentage of subjects with a lower than average number of teeth (60.2% [n. = 516/906] vs. 47.2% [n. = 326/714 ], *p <* 0.001) [102]. Conversely, higher income increased the likelihood of a functional dentition (≥20 remaining teeth) (OR 1.39 [1.24–1.56], *p <* 0.05; 9564 participants) [158]. In terms of the number of residual teeth, low-income people were more likely to show fewer teeth than control group individuals (24.48 SD 0.05 vs. 21.81 SD 0.13, *p <* 0.001; 36,026 participants) [152]. This increased tooth loss was also expressed in terms of mean difference (MD −0.69 [–1.22 to –0.15], *p <* 0.05) [153] and of higher probability (mean ratio 1.97, [CI 1.76–2.20], *p <* 0.001; 9564 participants) [154].

#### 3.6.4. Ethnic/Race Minorities

White people compared to black people (ethnic minority) showed a lower prevalence of loss of functional dentition with ≤20 residual teeth (17.8% [n. = 529/2959] vs. 50.3% [n. = 255/507], *p <* 0.001) [159], as well as an increased probability of maintaining a functional dentition (OR 1.10 [1.01–1.20], *p <* 0.05; 9564 participants) [158]. Additionally, in a study conducted on 6366 Brazilians, ethnic minorities were more likely to lose functional dentition in adulthood (40 years) than non-Hispanic whites, who represented the reference group, with an odds ratio that ranged from 1.41 (0.88–2.25) to 2.55 (1.39–4.66) [157].

#### 3.6.5. Populations Living in Rural Areas

Living in a rural area was a risk factor for tooth loss (OR 0.68 (0.56–0.82), *p <* 0.05; 3255 participants) [102], while residing in an urban area was a protective factor (OR 1.22 (1.02–1.46), *p* = 0.021; 3767 participants) [160]. Even in the most severe cases of tooth loss (more than half of teeth being missing), the rural population was more affected than the urban population (70% [n. = 1576/2796] vs. 52.8% [1220/2796], *p <* 0.01) [161].

#### 3.6.6. Subjects with Precarious/Unhealthy Working Conditions

Two studies have dealt with tooth loss in this type of vulnerability. In one study, unskilled workers, when compared with professionals, showed a higher probability of not functional dentition with <20 remaining teeth (OR 1.74 [1.15–2.62], *p* = 0.009; 592 participants) [162]. In the second study, lack of job stability was described as related to greater tooth loss in both males (OR 1.55 [1.18, 2.04], *p <* 0.05) and females (OR 1.44 [1.16, −1.79], *p <* 0.05), evaluating2652 participants [163].

#### 3.6.7. Subjects with Negative Experiences in Childhood

In a study of 6427 participants, the likelihood of partial or total tooth loss by the age of 50 was increased in those individuals who during their childhood experienced the loss of at least one parent or their divorce (OR 1.93 [1.52–2.46], *p <* 0.001), physical abuse (OR 1.33 [1.03–2.06], *p <* 0.05), cigarette smoking (OR 1.76 [1.29–2.39], *p <* 0.01) or poverty (OR 1.81 [1.37–2.38], *p <* 0.001) [102].

### 3.7. Dental Service Attendance

**Figure 5:**
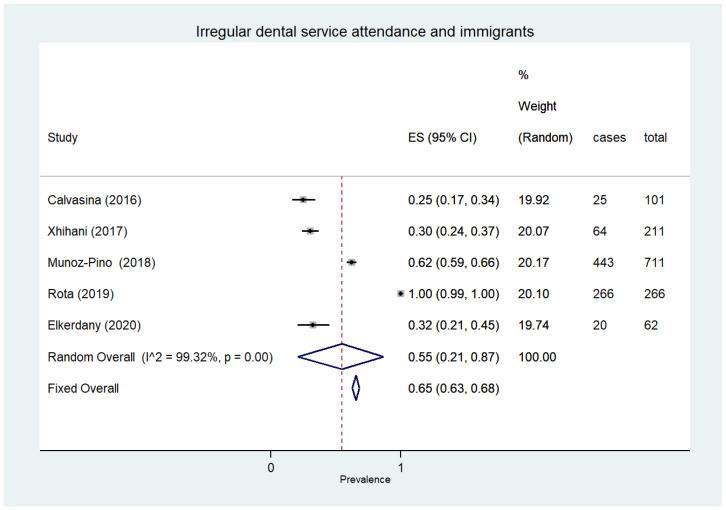
Irregular dental service attendance and immigrants.

All four types of socially vulnerable individuals where this outcome was noted showed a high degree of irregular dentist attendance, involving more than half of the participants evaluated. Specifically, this bad habit affected 75%of the homeless ([95% CI 73–76%]; 2530 participants, two studies) [67,68], 66% of pregnant women ([95%CI48–82]; 5103 participants, eight studies, I^2^ = 99%) [54,83,84,164,165,166,167,168], 55% of immigrants ([95% CI 21–87%]; 1351 participants, five studies) [51,169,170,171,172] (Figure 5) and 54% of the elderly ([95% CI 43–65%]; 33,430 participants, 19 studies, I^2^ = 99.7%) [33,36,41,46,47,90,91,96,97,106,110,116,126,132,139,142,173,174,175].

Furthermore, socially vulnerable people showed a greater tendency to visit the dentist more irregularly than the general population. A low level of education, for example, was identified as a factor that hindered a regular patient–dentist relationship (OR 0.68, 95% CI 0.48–0.95, *p* = 0.004) [176], favoring habits of irregular attendance of the dental service (OR 1.33 [1.26–1.40], *p*≤ 0.01; 90,845 participants) [177]. On the contrary, a high cultural level facilitated a regular relationship with the dentist (OR 2.07 [1.85–2.31], *p* = 0.001; 12,532 participants) [178]. Similarly, low income seemed to increase an irregular dental service attendance, with the data expressed in terms of odds ratio (OR 2.8 [95% CI 0.8–4.2], *p <* 0.07; 100,709 participants, four studies, I^2^ = 99.3%) [142,169,177,179] or risk ratio (RR 2.44 [2.17–2.73], *p <* 0.05; 3323 participants) [179], while high income was a preventive factor favoring a regular dental service attendance (OR 0.855 [0.803–0.911] *p <* 0.001; 12,532 participants) [177]. Considering the phenomenon of immigration, people living in countries hosting immigrants are more likely to visit the dentist regularly than immigrants (OR 1.24 [1.14–1.35], *p <* 0.05; 2648 participants) [180]. Finally, the lack of insurance coverage contributed to an increase in the irregular frequency of dental services (OR 6.54 [3.0, 14.33], *p <* 0.05; 154 participants) [179]. On the contrary, insurance coverage was a factor favoring regular visits to the dentist, with this outcome being described in terms of increased prevalence (72.3% vs. 53.3%, *p <* 0.001) [181] and probability of visiting the dentist (RR 1.8 [95% CI 1.4–2.3], *p <* 0.05; 2702 participants, two studies, I^2^ = not assessable) [175,182]. The type of insurance also affected the degree of attendance, and in fact, individuals with private insurance showed a greater aptitude to visit the dentist regularly than those covered by public insurance such as Medicare (OR 1.25–1.95), *p <* 0.001) or Medicaid (OR 1.29 (1.10–1.51), *p* = 0.001), as reported in a study conducted in the USA on a population sample of 402,077 participants [183].

### 3.8. Oral Health-Related Quality of Life (OHRQoL)

This outcome was described in three types of vulnerable individuals. In the elderly, one third of the subjects examined showed a reduced quality of life due to oral health problems such as pain, difficulty chewing or swallowing and psychological and/or relational discomfort due to the condition of their teeth (28% [95% CI 19–38]; 16,672 participants, 11 studies, I^2^ = 99.3%) [45,111,116,125,132,140,184,185,186,187,188], while the prevalence of this outcome in immigrants was 55% ([95% CI 21–87%]; 709 participants, three studies, I^2^ = 99.3%) [52,189,190]. Finally, in the only comparative study carried out, subjects living in rural areas compared to those in urban areas presented a worse quality of life related to dental or periodontal disorders measured with a specific scale called the Oral Health Impact Profile—14-item questionnaire (11.49 SD 9.733 vs. 5.88 SD 5.588, *p <* 0.001; 100 participants) [191].

### 3.9. Oral Health Perception

The most relevant data were reported in pregnant women, where about half of them showed a worse perception of their oral health (45% ([95% CI 25–66%]; 515 participants, four studies, I^2^ = 95.7%) [54,55,167,168], while this negative perception was less prevalent among the elderly (28% [95% CI 19–38%]; 67,049 participants, 20 studies, I^2^ = 99.3%) [28,36,38,41,45,46,92,96,111,113,123,125,139,140,143,173,174,192,193,194].

When compared, socially vulnerable individuals presented a worse perception of their oral health status than the general population. This increased degree of negative perception was expressed in terms of prevalence (77.5% n. = 224/631 vs. 22.5% n. = 65/342, *p <* 0.001) [195], absolute difference (AD 20.49% [4.95–36.4]; 14,960 participants) [196] and probability, as published in two cross sectional studies (OR 2.8 [95% CI 0.5–14.7], *p* = 0.073; 65,363 participants, I^2^ = 99.0%) [180,197] and in a prospective study (RR1.61 [1.28–2.03] *p <* 0.05; 2812 participants) [155].This perception of poor oral health was also found to be more likely in low-income subjects when compared with the reference population, with this outcome being expressed in terms of odds ratio (2.2 [95% CI 1.7–2.6], *p <* 0.05; 68,966 participants, four studies, I^2^ = 61.2%) [180,197,198,199], risk ratio (3.18 [2.13–4.74], *p <* 0.05; 2812 participants) [155] and absolute difference (AD 15.77% [12.77–18.77]; 14,960 participants) [196]. Even among skilled workers subjected to stress due to excessive working hours (>40 h of overtime), an increased negative perception of their oral health was found compared to subjects working in less stressful conditions (OR 3.25 (1.66–6.35), *p <* 0.001; 950 participants) [79,80].

### 3.10. Emergency Room Treatments Due to Non-Traumatic Injuries

In two groups of socially vulnerable individuals, a greater use of emergency room treatment for non-traumatic dental injuries was demonstrated than with the non-vulnerable population, specifically black individuals (ethnic minority), who had nearly five times more visits (1118 participants) [200], and those without any health insurance, as expressed in the studies of Franciscatto and co-workers (OR 1.36 [1.24–1.49], *p <* 0.001; 6282participants) and of DeLia (2015) (*p <* 0.001; 96,787 participants) [201,202].

### 3.11. People with Multiple Social Vulnerabilities

Where subjects with two (or more) social vulnerabilities were compared with those with a single vulnerability, the former showed a worse dental and/or periodontal health status. For example, when older people with a low level of education were compared with those who were more educated, a higher prevalence of caries was found. (30.8% [n. 961/3119] vs. 11.7% [n. = 365/3119], *p <* 0.05) [118], while a high level of education was found as a preventive factor against caries for elderly people (OR 0.81 [0.74–0.89], *p* = 0.001; 4431 participants) [29]. Similarly, low income, when added to old age, increased the prevalence of caries (28.6% [n. = 892/3119) vs. 9.9% [n. = 365/3119], *p <* 0.05) [118]. In a study conducted by Martin and co-workers, older non-Hispanic whites residing in urban areas appeared less affected by tooth decay than Mexican-American or black (race minorities) elderly people residing in rural areas (*p <* 0.05) [36]. Education was also noted as a preventive factor against severe periodontal disease in the elderly (*p* = 0.004; 30,427 participants) [203], decreasing the likelihood of suffering from this oral disease (RR 0.96 [0.95–0, 97], *p <* 0.05; 2332 participants) [89]. Low income also seemed to increase the prevalence of periodontitis in the elderly (*p <* 0.001; 687 participants) [43] as well as the likelihood of suffering from this disease (OR 1.39 [1.01–1.91], *p <* 0.05; 2147 participants) [204], while high income was a prevention factor against periodontitis (RR 0.91 [0.86 0.96], *p <* 0.05; 2332) [89]. Finally, a rural environment seemed to make the elderly more susceptible to periodontitis than an urban environment (*p* = 0.007; 30,427 participants) [205].

Furthermore, low income in pregnant women was a risk factor for periodontitis (OR 0.61 [0.46–0.81], *p* = 0.001, 160 participants) [88]. Similarly, poor quality of life related to oral health in pregnant women appeared to be linked to unemployment status, low levels of education and immigration status (*p <* 0.05) [167].

### 3.12. Quality Assessment of Included Studies

Using JBI’s Critical Appraisal Tool Checklist, the following considerations were performed on the study methodology of included papers (see Supplementary Table S2). Of the 168 included cross-sectional studies, 126 studies met 6/8 criteria and 42 studies met 8/8 criteria. Of the nine included cohort studies, seven studies met 9/11 criteria and two studies met 11/11 criteria. Of the four included case–control studies, all the studies met 8/10 criteria. Of the 181 selected articles, in 137 studies confounding factors were not clearly investigated.

## 4. Discussion

The socially vulnerable who live on the margins of society with reduced resilience to adverse events constitute a very heterogeneous typology of individuals with different traits and habits regarding age, sex, living environment, origin, socio-economic status and beliefs. Despite these differences, in this systematic review of the literature, it was found that socially vulnerable subjects showed shared traits in terms of their oral health. The first common trait was a higher prevalence and/or severity of caries and periodontal disease (and related tooth loss) compared to the general population. Specifically, our study showed that caries in vulnerable individuals, with a prevalence ranging between 39% and 77.4%, was greater than 29.4% of the general population, indicating the global burden of this disease in 2017 [206]. This increased prevalence was also confirmed by studies included in this review, which made a direct comparison between socially disadvantaged and non-disadvantaged individuals. Additionally, in relation to the severity of the caries (measured by the DMFT index), socially disadvantaged individuals showed higher scores than non-vulnerable people, with values that in most of the different types of vulnerable subjects ranged between 11.8 and 18.7.

A similar epidemiological pattern was found in periodontitis, with higher prevalence scores for socially disadvantaged subjects showing percentages between 43% and 60%, with the exception of pregnant women and low-income subjects where the percentages were less. As with caries and periodontitis, complete edentulism among the vulnerable (where prevalence ranged from 16 to 27% in our study) showed significantly higher scores than the 3.3% of the general population, as reported in 2017 [206]. Additionally, taking into account the partial edentulism in its two clinical variants (<21 or <9 residual teeth in the oral cavity), socially vulnerable people were more affected than individuals with better social conditions.

Other common traits found in different types of vulnerable people were the perception of an unhealthy oral state and a poor oral health-related quality of life (OHRQoL). Moreover, socially vulnerable people were less likely to visit the dentist regularly than non-vulnerable ones. This bad habit makes it more difficult for dental professionals to protect their compromised oral health. Probably a new model of health policy should be developed (in addition to the existing one) with solutions able to meet socially vulnerable subjects in their environment without waiting for the patient to visit the dental office. In other words, this health policy should be based more on education, prophylaxis and minimally invasive treatment of oral diseases, also using innovative transportable devices and instruments in order to reach people living on the margins of the social network. Atraumatic restorative treatment (ART) is the best known of these types of easy intervention, which can be performed outside the dental office [207].

Another shared trait noted among socially disadvantaged subjects was the presence of a worsening of dental–periodontal health when more social vulnerabilities are pooled in the same individuals. This particular worsening is not unlike that described in the literature where multiple health vulnerabilities (with chronic illnesses) occur in a single subject [208].

In this study, we did not include disabled people among the categories of social vulnerability, as they may be affected by a double vulnerability, social and sanitary, at the same time. However, it must be taken into account that social and medical vulnerability combined increase the risk factors for caries and periodontal disease [209].

In recent years, the literature has shown that caries and periodontal disease cause, as a complication, an increased risk of onset of multiple systemic diseases such as cardio-vascular disease, dysmetabolic syndrome, respiratory tract infection and preterm birth in the general population [210,211,212,213]. Based on data described in this review, the risk of these complications should be considered increased for socially disadvantaged subjects due to their more compromised oral health status. A further complication for the vulnerable could be the psychological impact of a bad dental condition (bad smile) on self-esteem, which could worsen their social exclusion situation [214].

No reviews have been found in the literature that dealt with dental–periodontal health in socially vulnerable individuals as a whole. However, some reviews were found that reported scientific evidence of oral health for single individual types of socially disadvantaged individuals. These reviews provided overall results in agreement with the present study in highlighting a health disparity between socially vulnerable and non-vulnerable individuals. The concordance of the results between this review (consisting of studies published in the last five years) and the others found in the literature (including most studies prior to the last five years) showed that the problem of oral health in socially disadvantaged subjects did not improve over time. On the contrary, there is a growing social polarization of dental–periodontal diseases (as demonstrated in this review), even in the context of global improvement concerning caries and periodontitis [215].

### Limitations

1. Considering the scope of providing an overall view of dental–periodontal health involving all types of socially vulnerable subjects, this review does not present a complete picture for two reasons. The first reason is that in the literature (consequently in our review), not all types of vulnerable subjects are mentioned in relation to their dental–periodontal health. The second reason is that in many groups of vulnerable subjects, not all the dental–periodontal health outcomes (those indicated in the “Materials and Methods” section of this review) were described, and in some cases even the primary ones were missing.
2. The high level of heterogeneity (I^2^ > 90%) of results relative to the majority of the outcomes in this review reduces the reliability of conclusions on the dental–periodontal health of vulnerable subjects, who were the focus of our research
3. This systematic review did not take into account the differences between developing and underdeveloped countries in the assessment of socially vulnerable people. This was to limit any subgroup analysis (especially in some groups of vulnerable subjects described by only a few studies with a reduced number of participants) in order to avoid the risk of compromising the accuracy of the results by reducing the sample size of enrolled individuals.

## 5. Conclusions

Despite a certain degree of incompleteness of the data due to the complexity of this topic, the results of our review supported the hypothesis of a more compromised dental–periodontal status in socially disadvantaged subjects than in the non-vulnerable population. The increased risk of dental–periodontal diseases was a finding found in every type of social vulnerability and seemed to worsen in the presence of pooled vulnerabilities. Furthermore, according to the results of this review, to date the health policies adopted by the National Health Systems in most countries seem inadequate to reduce the inequalities in oral health between socially vulnerable and non-vulnerable people. Therefore, the results of this review will hopefully stimulate health policy makers to adopt measures, both legislative and economic, which are more protective of socially vulnerable subjects. In fact, the loss of oral health inevitably creates a negative impact on the overall health of the vulnerable worldwide, with significant financial consequences for National Health Systems and for the global general population.

## Supplementary Materials

The following are available online at https://www.mdpi.com/article/10.3390/ijerph182312360/s1, Table S1: Strategy search, Table S2: Quality assessment, Figure S1: Statistical analysis not included in the main text, Table S3: PRISMA checklist.
