# Supplementary material for: Systematic Review of the Literature on Dental Caries and Periodontal Disease in Socio-Economically Disadvantaged Individuals
Source: Int J Environ Res Public Health. 2021 Nov 24;18(23):12360. doi: 10.3390/ijerph182312360 (PMC8656978; doi:10.3390/ijerph182312360)
Supplement: Supplementary file 1 [file ijerph-18-12360-s001.zip › Supplementary Figure S1.pdf]

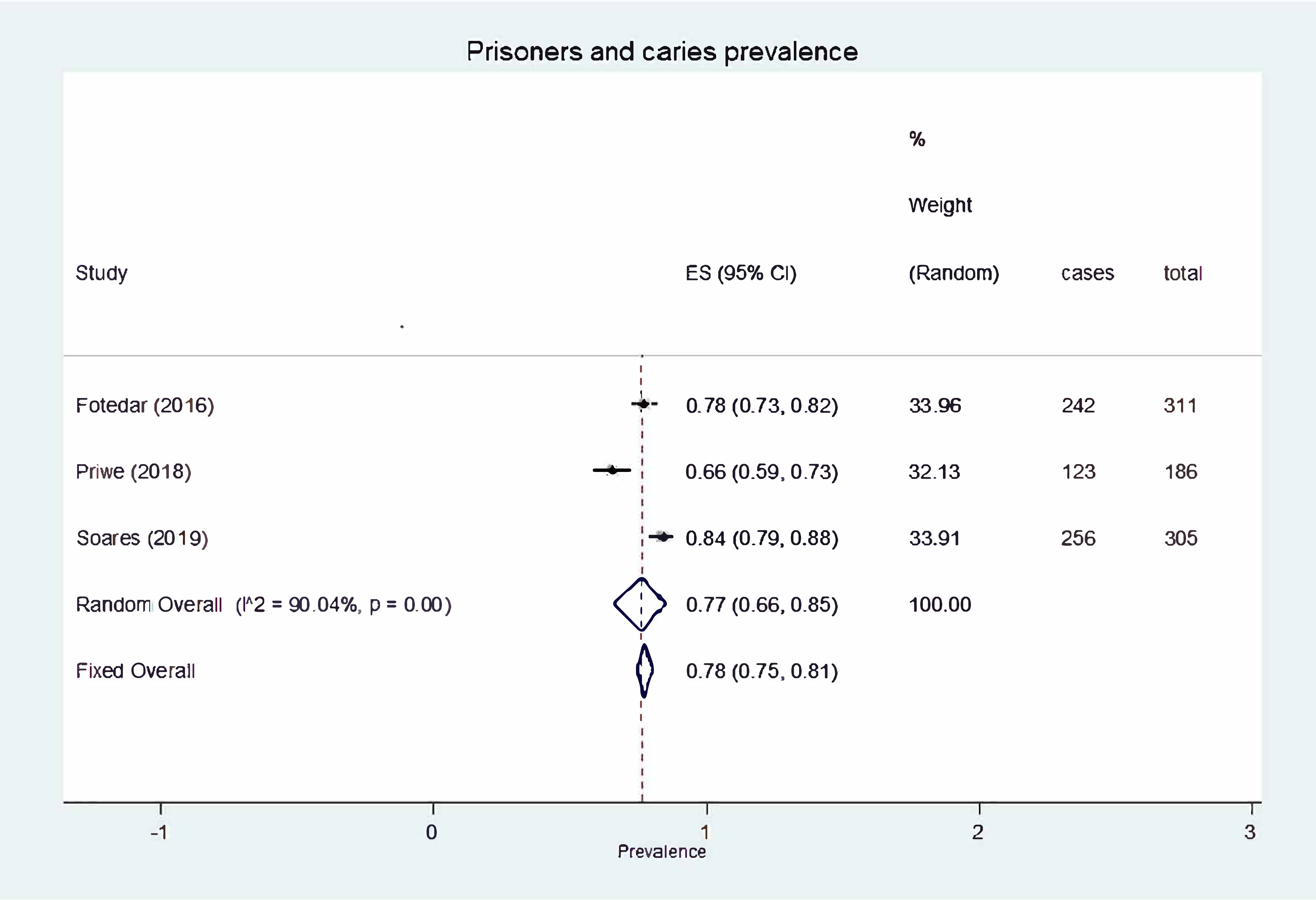
Prisoners and caries prevalence
%
Weight
Study
ES (95% CI)
(Random)
cases
total
Fotedar (2016)
0.78 (0.73, 0.82)
33.96
242
311
Priwe (2018)
0.66 (0.59, 0.73)
32.13
123
186
Soares (2019)
0.84 (0.79, 0.88)
33.91
256
305
Random Overall (I^2 = 90.04%, p = 0.00)
0.77 (0.66, 0.85)
100.00
Fixed Overall
0.78 (0.75, 0.81)
-1
0
1
2
3
Prevalence

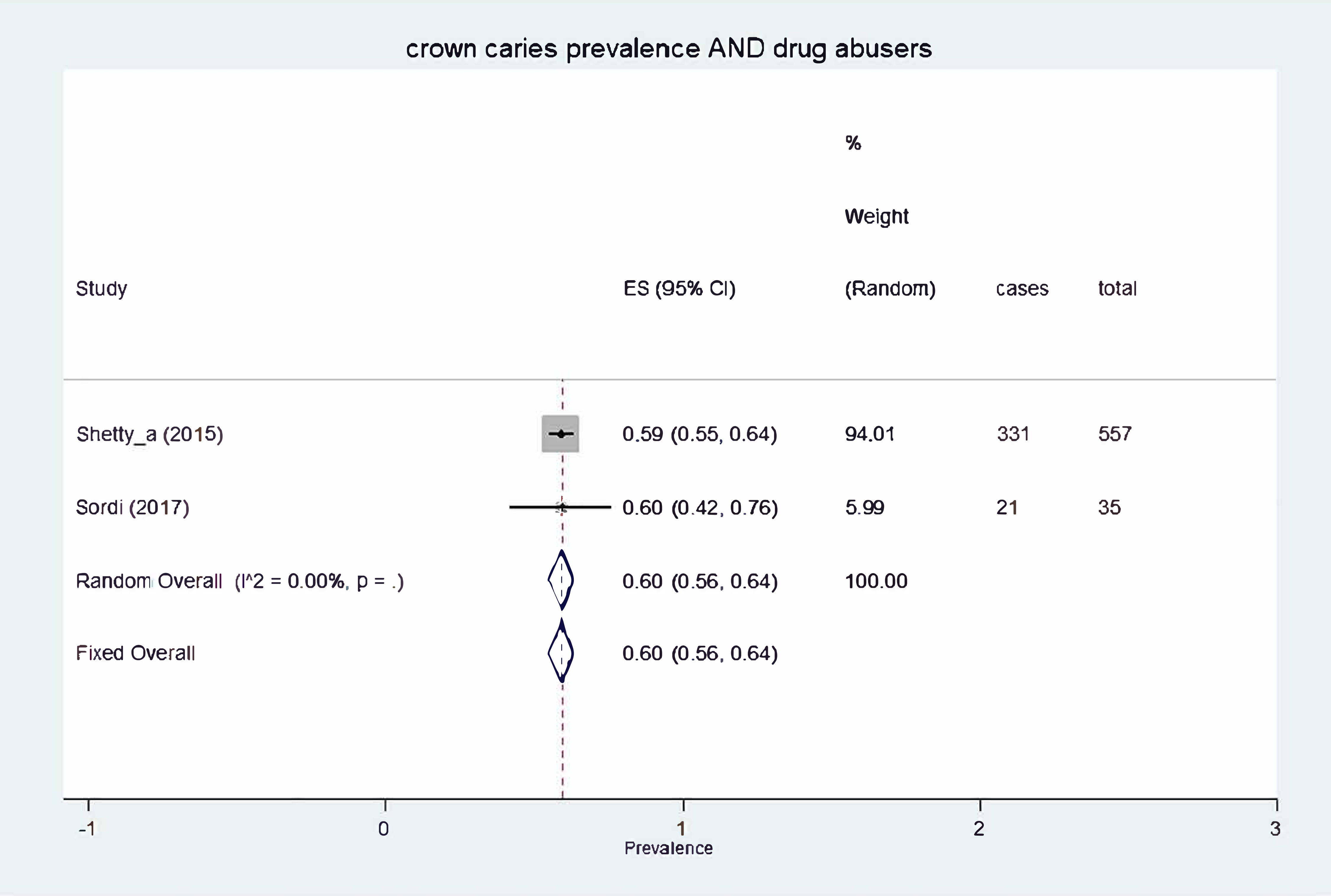
crown caries prevalence AND drug abusers
Study
ES (95% CI)
% Weight (Random)
cases
total
Shetty_a (2015)
0.59 (0.55, 0.64)
94.01
331
557
Sordi (2017)
0.60 (0.42, 0.76)
5.99
21
35
Random Overall (I^2 = 0.00%, p = .)
0.60 (0.56, 0.64)
100.00
Fixed Overall
0.60 (0.56, 0.64)
-1
0
1
2
3
Prevalence

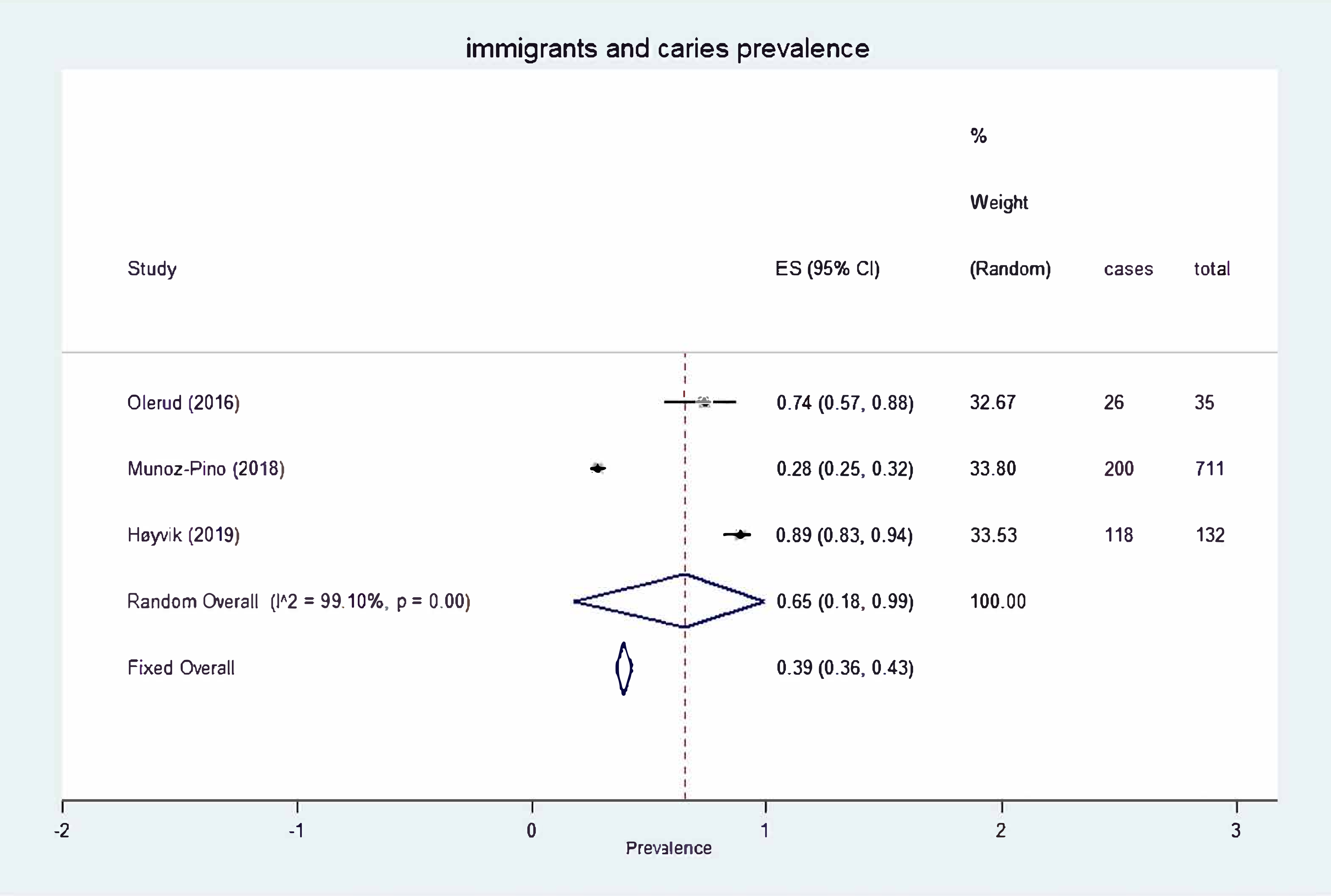
immigrants and caries prevalence
Study
ES (95% CI)
% Weight (Random)
cases
total
Olerud (2016)
0.74 (0.57, 0.88)
32.67
26
35
Munoz-Pino (2018)
0.28 (0.25, 0.32)
33.80
200
711
Høyvik (2019)
0.89 (0.83, 0.94)
33.53
118
132
Random Overall (I^2 = 99.10%, p = 0.00)
0.65 (0.18, 0.99)
100.00
Fixed Overall
0.39 (0.36, 0.43)
-2
-1
0
1
2
3
Prevalence

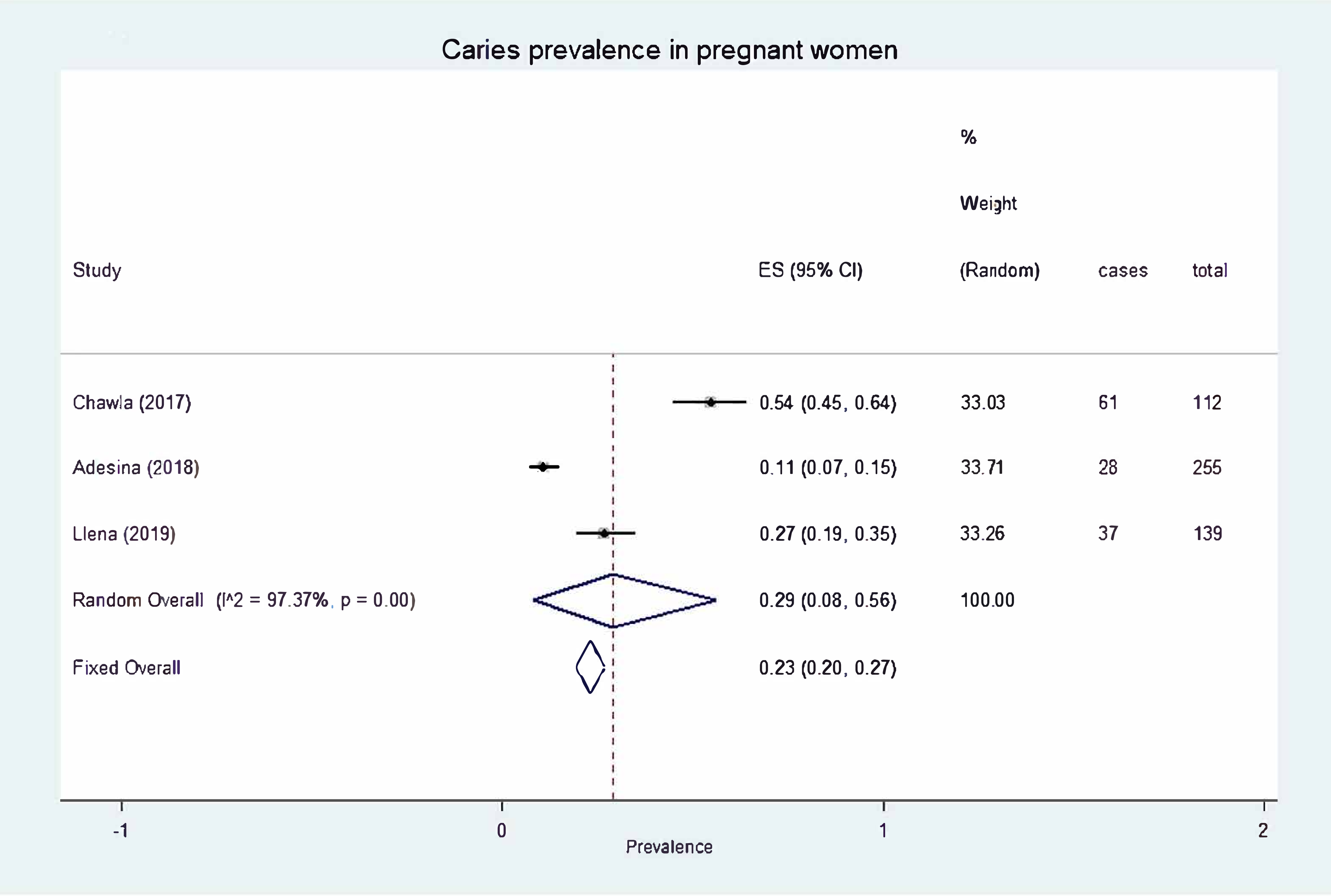
Caries prevalence in pregnant women
Study
ES (95% CI)
% Weight (Random)
cases
total
Chawla (2017)
0.54 (0.45, 0.64)
33.03
61
112
Adesina (2018)
0.11 (0.07, 0.15)
33.71
28
255
Llena (2019)
0.27 (0.19, 0.35)
33.26
37
139
Random Overall (I^2 = 97.37%, p = 0.00)
0.29 (0.08, 0.56)
100.00
Fixed Overall
0.23 (0.20, 0.27)
-1
0
1
2
Prevalence

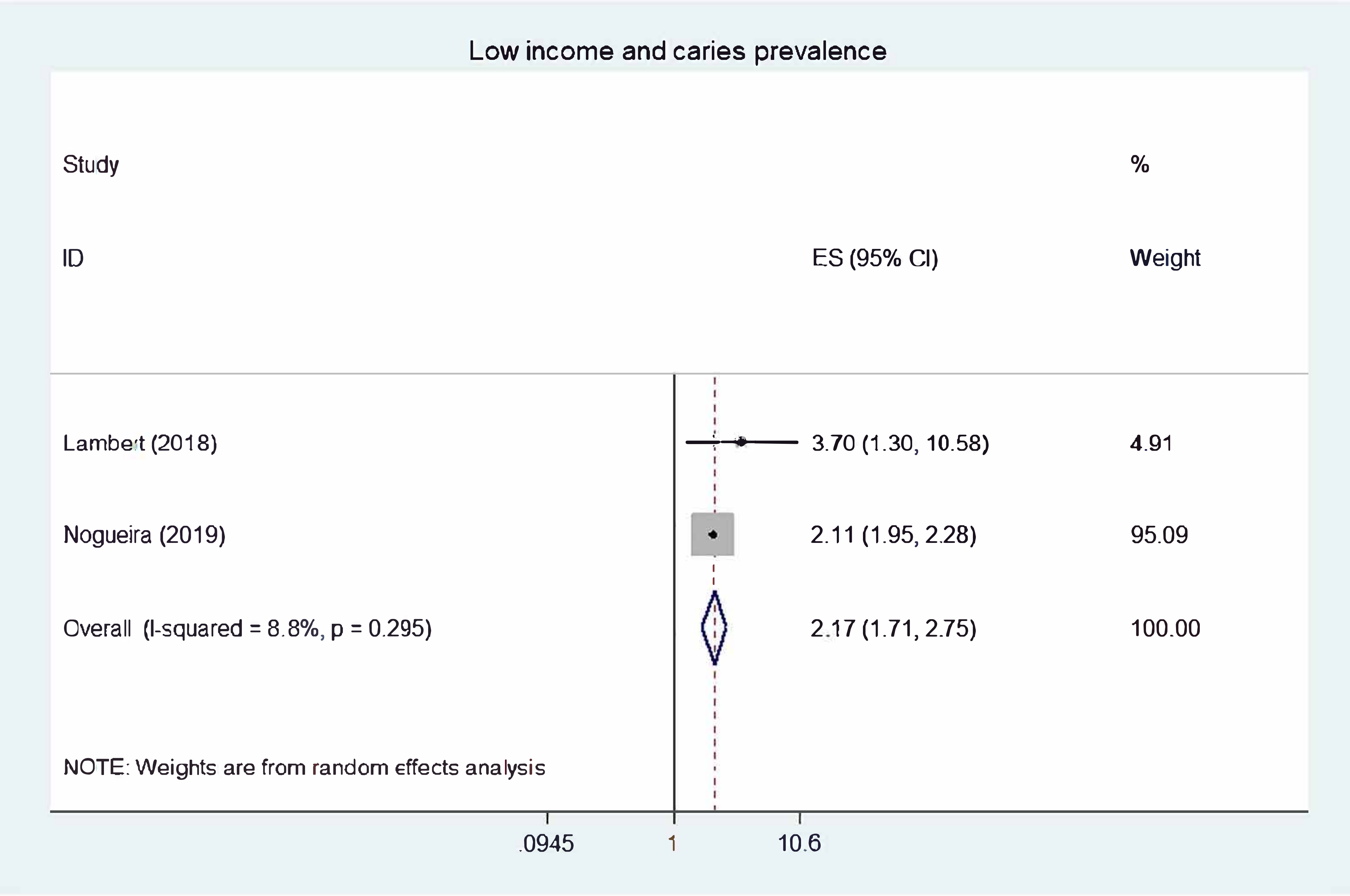
Low income and caries prevalence
Study
ID
ES (95% CI)
%
Weight
Lambert (2018)
3.70 (1.30, 10.58)
4.91
Nogueira (2019)
2.11 (1.95, 2.28)
95.09
Overall (I-squared = 8.8%, p = 0.295)
2.17 (1.71, 2.75)
100.00
NOTE: Weights are from random effects analysis
.0945
1
10.6

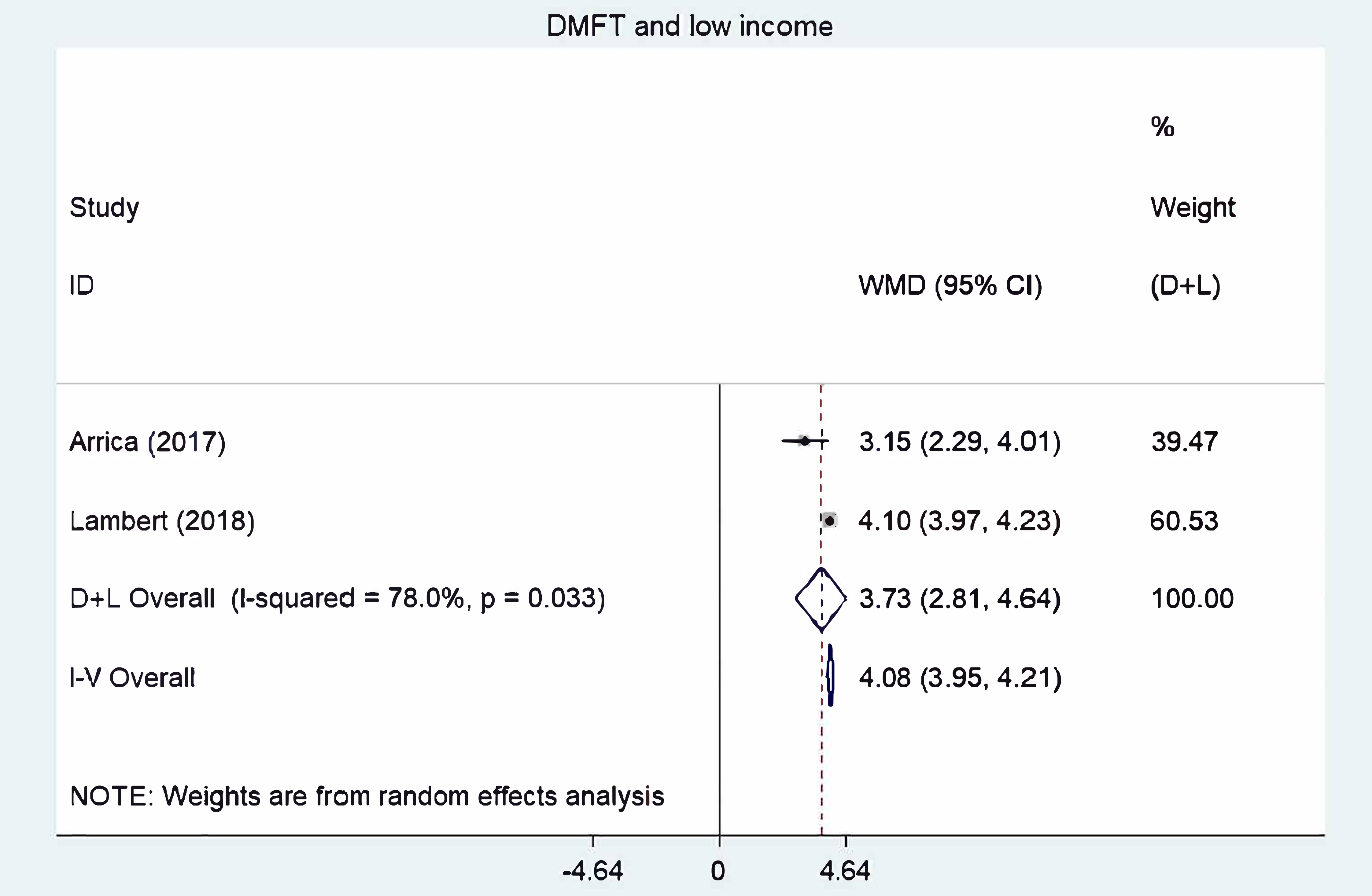
DMFT and low income
Study
ID
WMD (95% CI)
%
Weight
(D+L)
Arrica (2017)
3.15 (2.29, 4.01)
39.47
Lambert (2018)
4.10 (3.97, 4.23)
60.53
D+L Overall (I-squared = 78.0%, p = 0.033)
3.73 (2.81, 4.64)
100.00
I-V Overall
4.08 (3.95, 4.21)
NOTE: Weights are from random effects analysis
-4.64
0
4.64

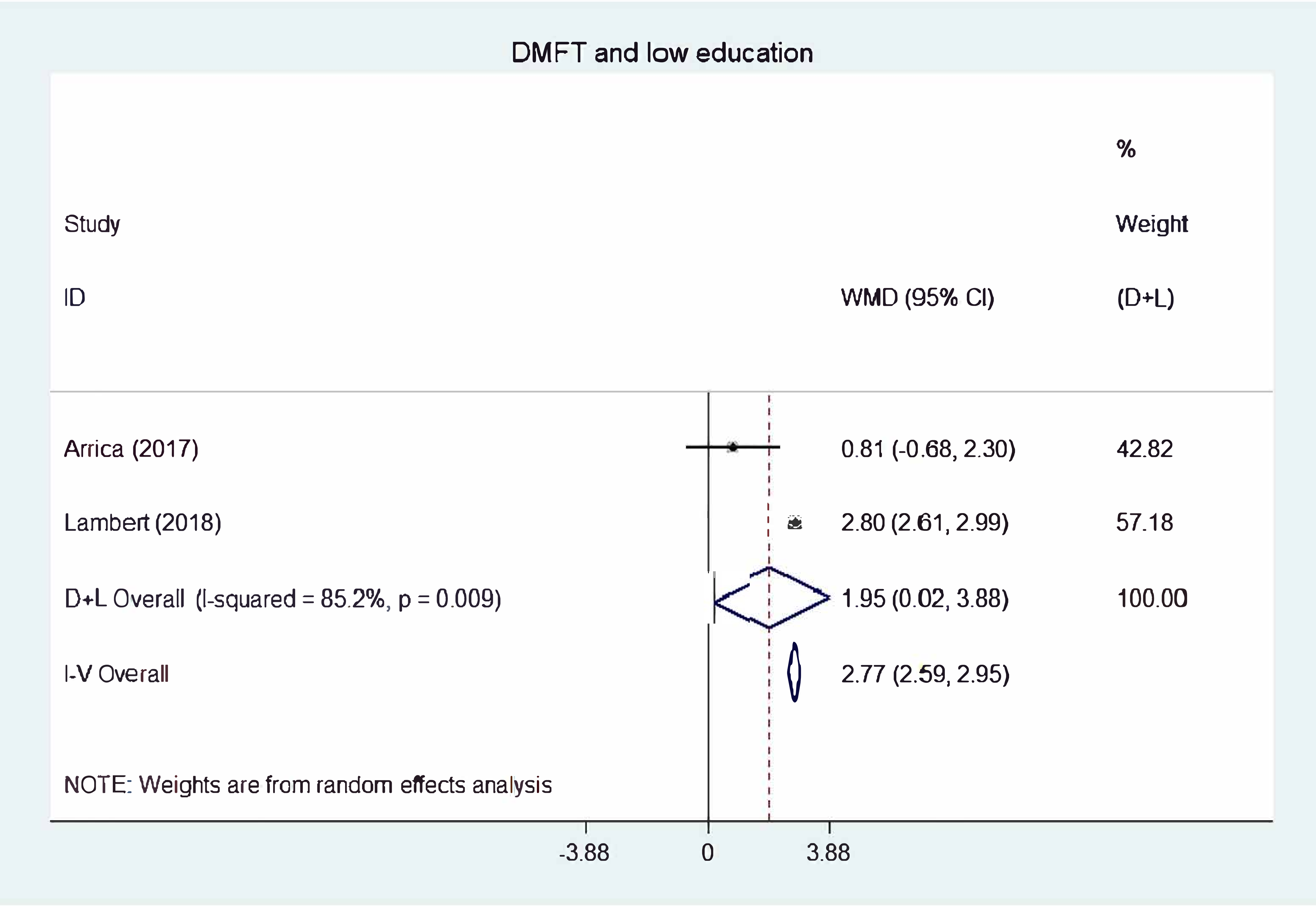
DMFT and low education
Study
ID
WMD (95% CI)
%
Weight
(D+L)
Arrica (2017)
0.81 (-0.68, 2.30)
42.82
Lambert (2018)
2.80 (2.61, 2.99)
57.18
D+L Overall (I-squared = 85.2%, p = 0.009)
1.95 (0.02, 3.88)
100.00
I-V Overall
2.77 (2.59, 2.95)
NOTE: Weights are from random effects analysis
-3.88
0
3.88

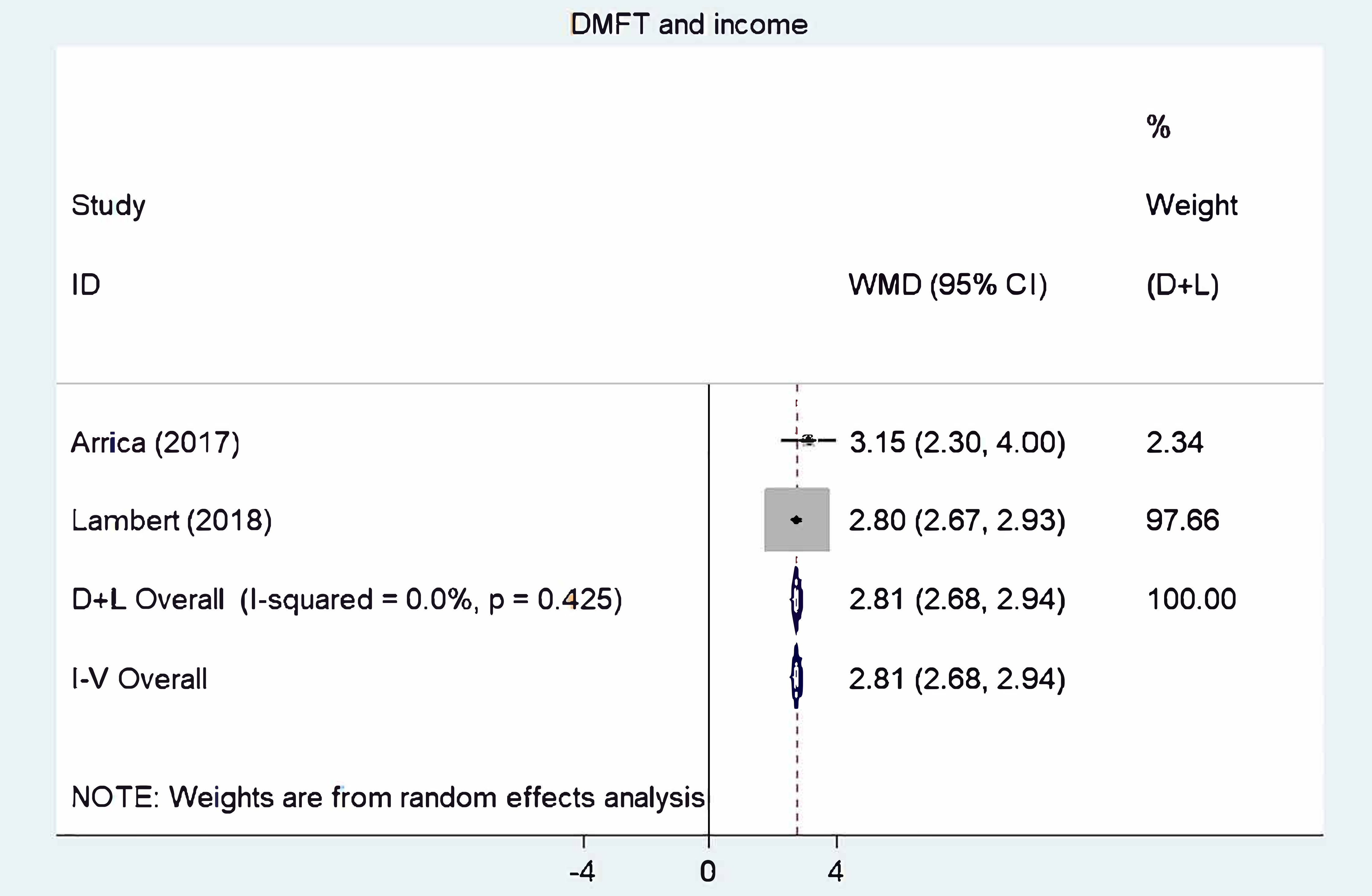
DMFT and income
%
Study
Weight
ID
WMD (95% CI)
(D+L)
Arrica (2017)
3.15 (2.30, 4.00)
2.34
Lambert (2018)
2.80 (2.67, 2.93)
97.66
D+L Overall (I-squared = 0.0%, p = 0.425)
2.81 (2.68, 2.94)
100.00
I-V Overall
2.81 (2.68, 2.94)
NOTE: Weights are from random effects analysis
-4
0
4

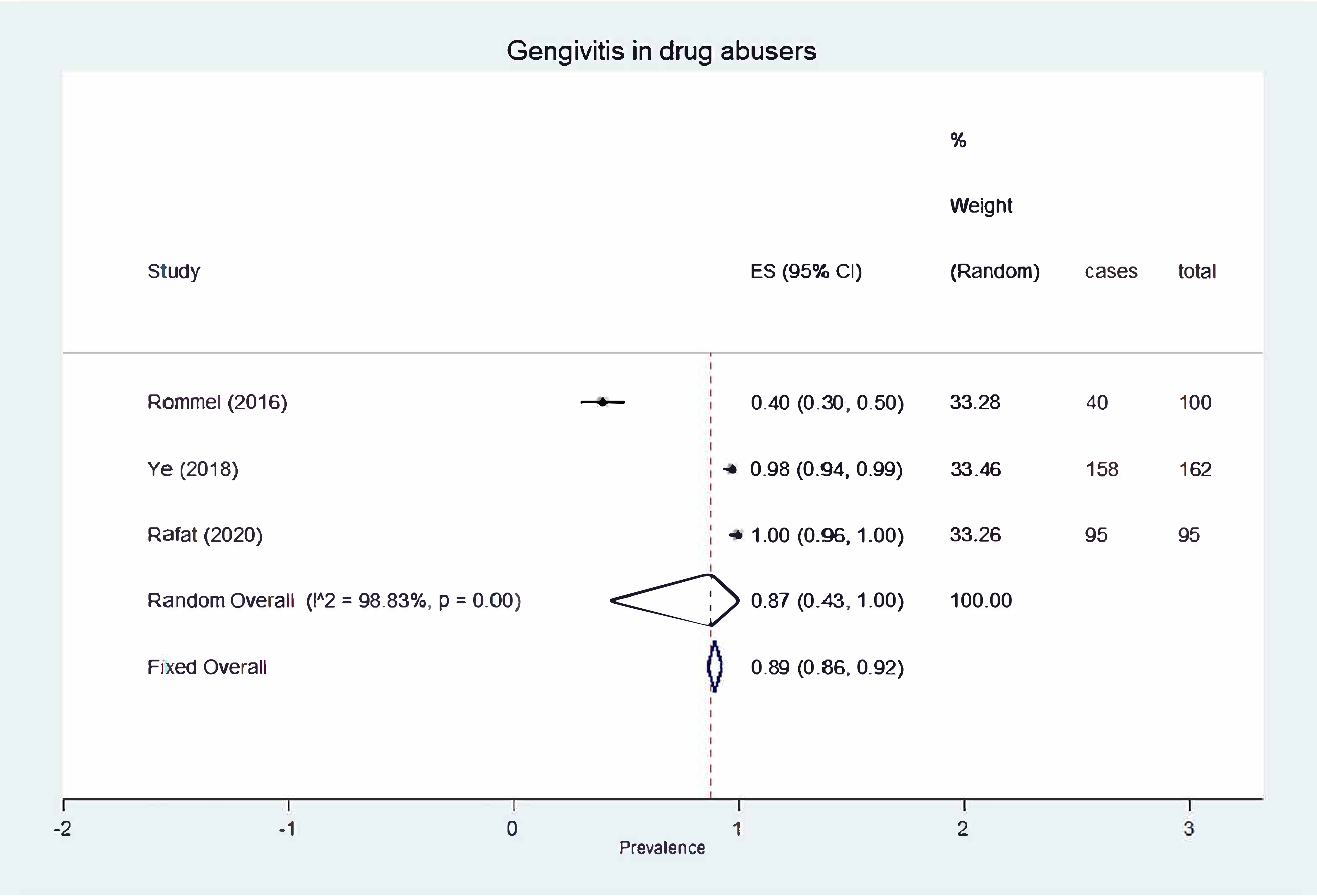
Gengivitis in drug abusers
Study
ES (95% CI)
% Weight (Random)
cases
total
Rommel (2016) 0.40 (0.30, 0.50) 33.28 40 100
Ye (2018) 0.98 (0.94, 0.99) 33.46 158 162
Rafat (2020) 1.00 (0.96, 1.00) 33.26 95 95
Random Overall (I^2 = 98.83%, p = 0.00) 0.87 (0.43, 1.00) 100.00
Fixed Overall 0.89 (0.86, 0.92)
-2
-1
0
1
2
3
Prevalence

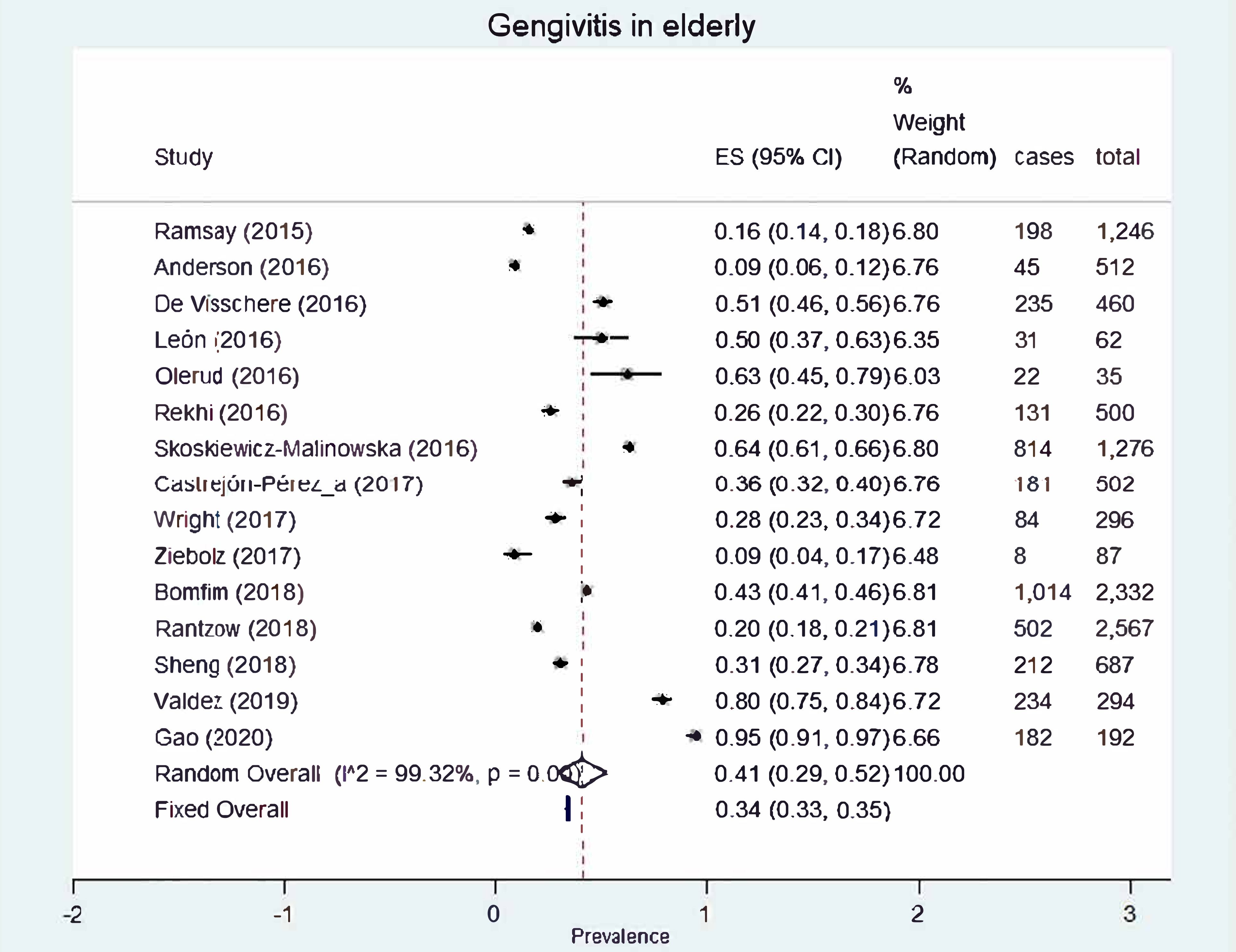
Gengivitis in elderly
Study
ES (95% CI)
% Weight (Random)
cases
total
Ramsay (2015) 0.16 (0.14, 0.18) 6.80 198 1,246
Anderson (2016) 0.09 (0.06, 0.12) 6.76 45 512
De Visschere (2016) 0.51 (0.46, 0.56) 6.76 235 460
León (2016) 0.50 (0.37, 0.63) 6.35 31 62
Olerud (2016) 0.63 (0.45, 0.79) 6.03 22 35
Rekhi (2016) 0.26 (0.22, 0.30) 6.76 131 500
Skoskiewicz-Malinowska (2016) 0.64 (0.61, 0.66) 6.80 814 1,276
Castrejón-Pérez_a (2017) 0.36 (0.32, 0.40) 6.76 181 502
Wright (2017) 0.28 (0.23, 0.34) 6.72 84 296
Ziebolz (2017) 0.09 (0.04, 0.17) 6.48 8 87
Bomfim (2018) 0.43 (0.41, 0.46) 6.81 1,014 2,332
Rantzow (2018) 0.20 (0.18, 0.21) 6.81 502 2,567
Sheng (2018) 0.31 (0.27, 0.34) 6.78 212 687
Valdez (2019) 0.80 (0.75, 0.84) 6.72 234 294
Gao (2020) 0.95 (0.91, 0.97) 6.66 182 192
Random Overall (I^2 = 99.32%, p = 0.00) 0.41 (0.29, 0.52) 100.00
Fixed Overall 0.34 (0.33, 0.35)
-2
-1
0
1
2
3
Prevalence

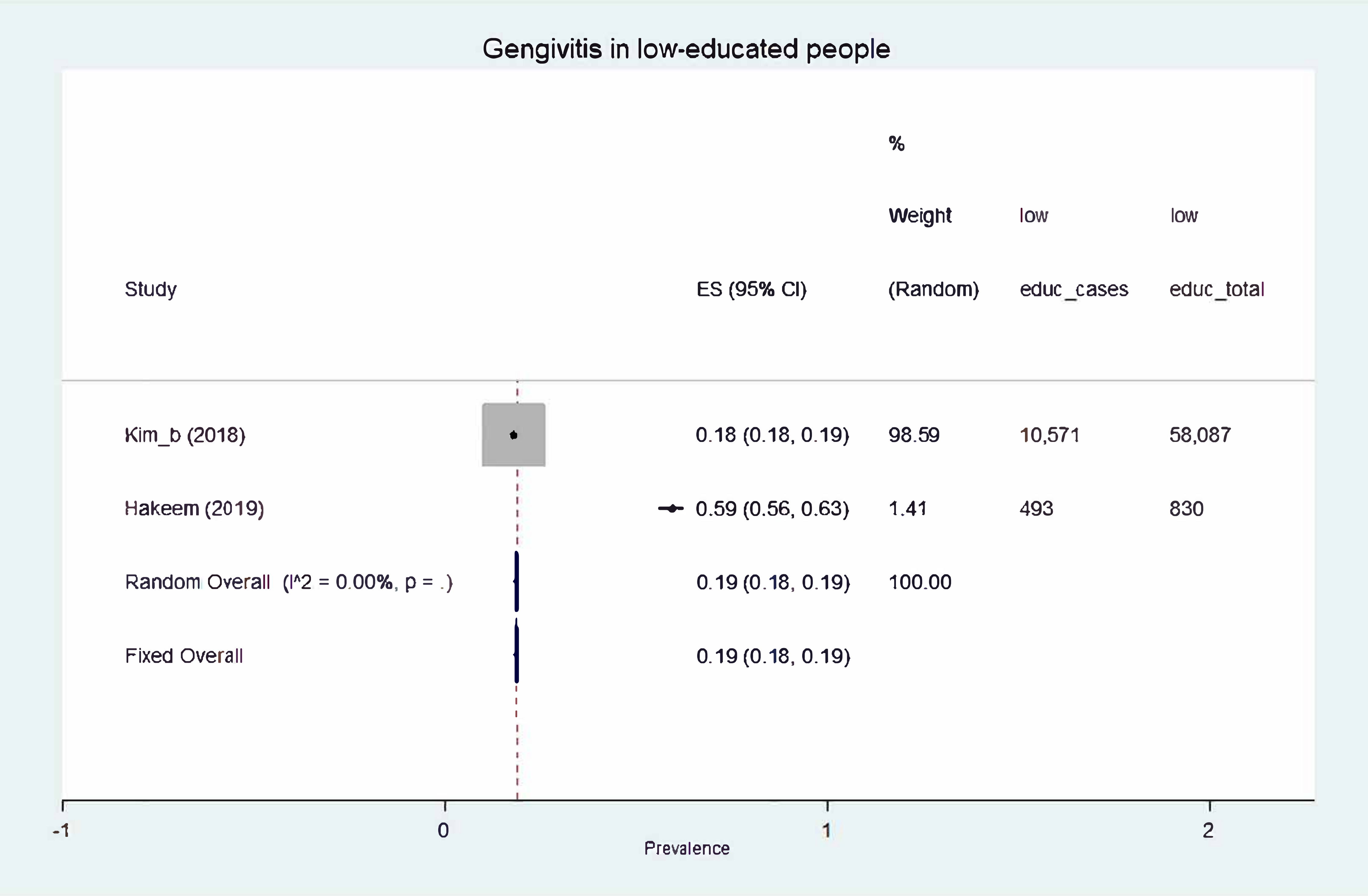
Gengivitis in low-educated people
Study
ES (95% CI)
%
Weight
(Random)
low
educ_cases
low
educ_total
Kim_b (2018)
0.18 (0.18, 0.19)
98.59
10,571
58,087
Hakeem (2019)
0.59 (0.56, 0.63)
1.41
493
830
Random Overall (I^2 = 0.00%, p = .)
0.19 (0.18, 0.19)
100.00
Fixed Overall
0.19 (0.18, 0.19)
-1
0
1
2
Prevalence

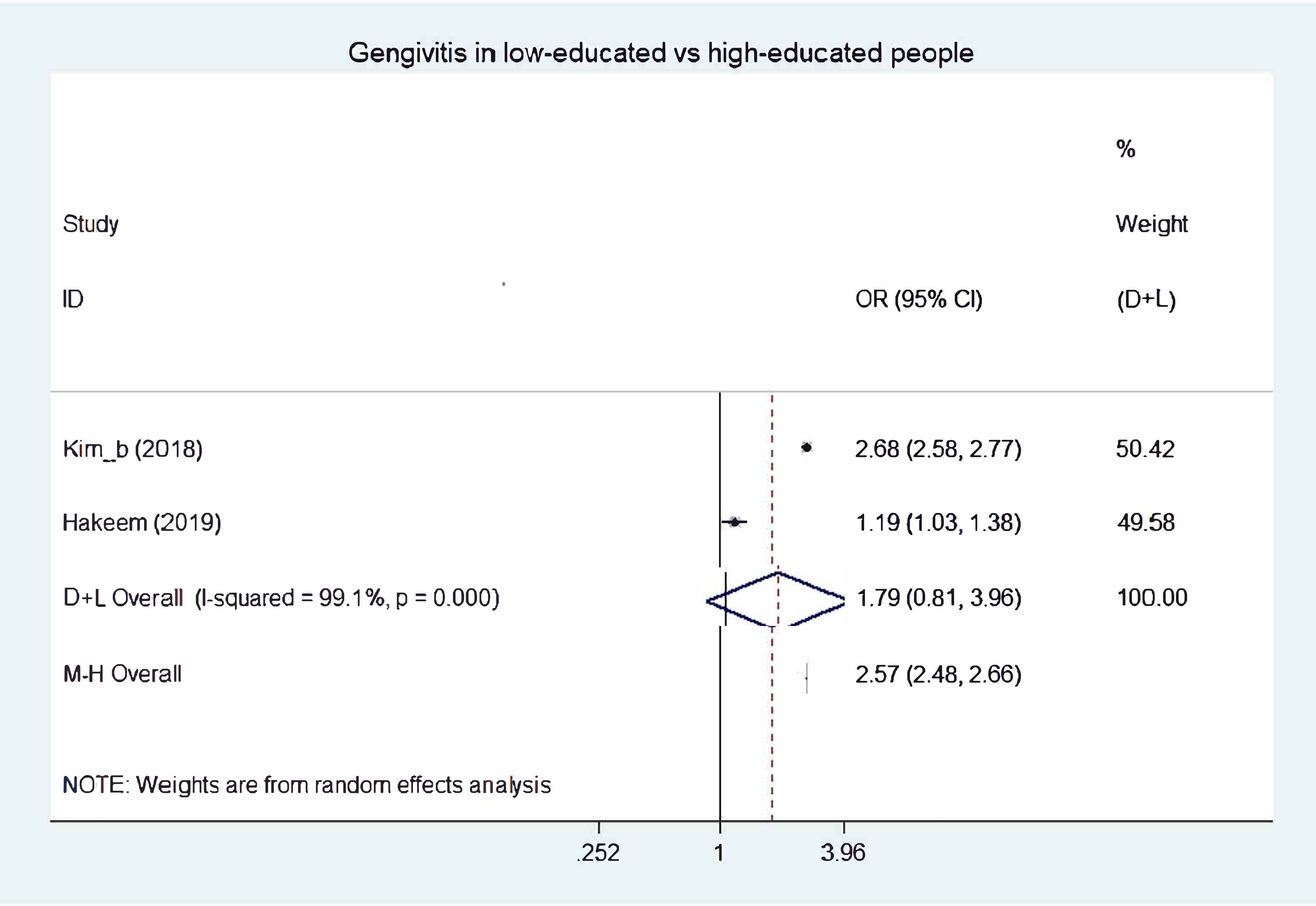
Gengivitis in low-educated vs high-educated people
Study
ID
OR (95% CI)
%
Weight
(D+L)
Kim_b (2018)
2.68 (2.58, 2.77)
50.42
Hakeem (2019)
1.19 (1.03, 1.38)
49.58
D+L Overall (I-squared = 99.1%, p = 0.000)
1.79 (0.81, 3.96)
100.00
M-H Overall
2.57 (2.48, 2.66)
NOTE: Weights are from random effects analysis
.252
1
3.96

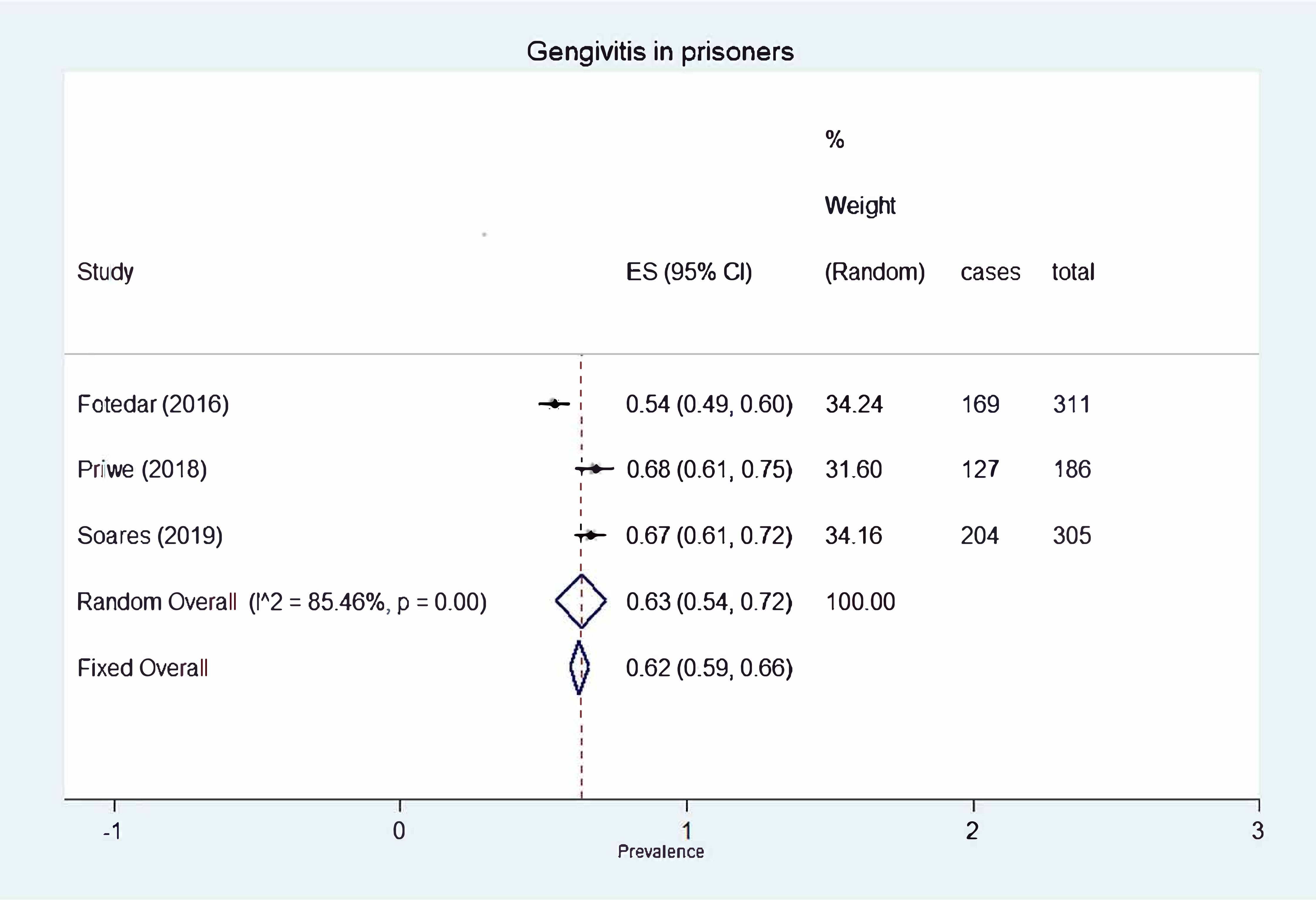

Gengivitis in prisoners
%
Weight
Study
ES (95% CI)
(Random)
cases
total
Fotedar (2016)
0.54 (0.49, 0.60)
34.24
169
311
Priwe (2018)
0.68 (0.61, 0.75)
31.60
127
186
Soares (2019)
0.67 (0.61, 0.72)
34.16
204
305
Random Overall (I^2 = 85.46%, p = 0.00)
0.63 (0.54, 0.72)
100.00
Fixed Overall
0.62 (0.59, 0.66)
-1
0
1
2
3
Prevalence

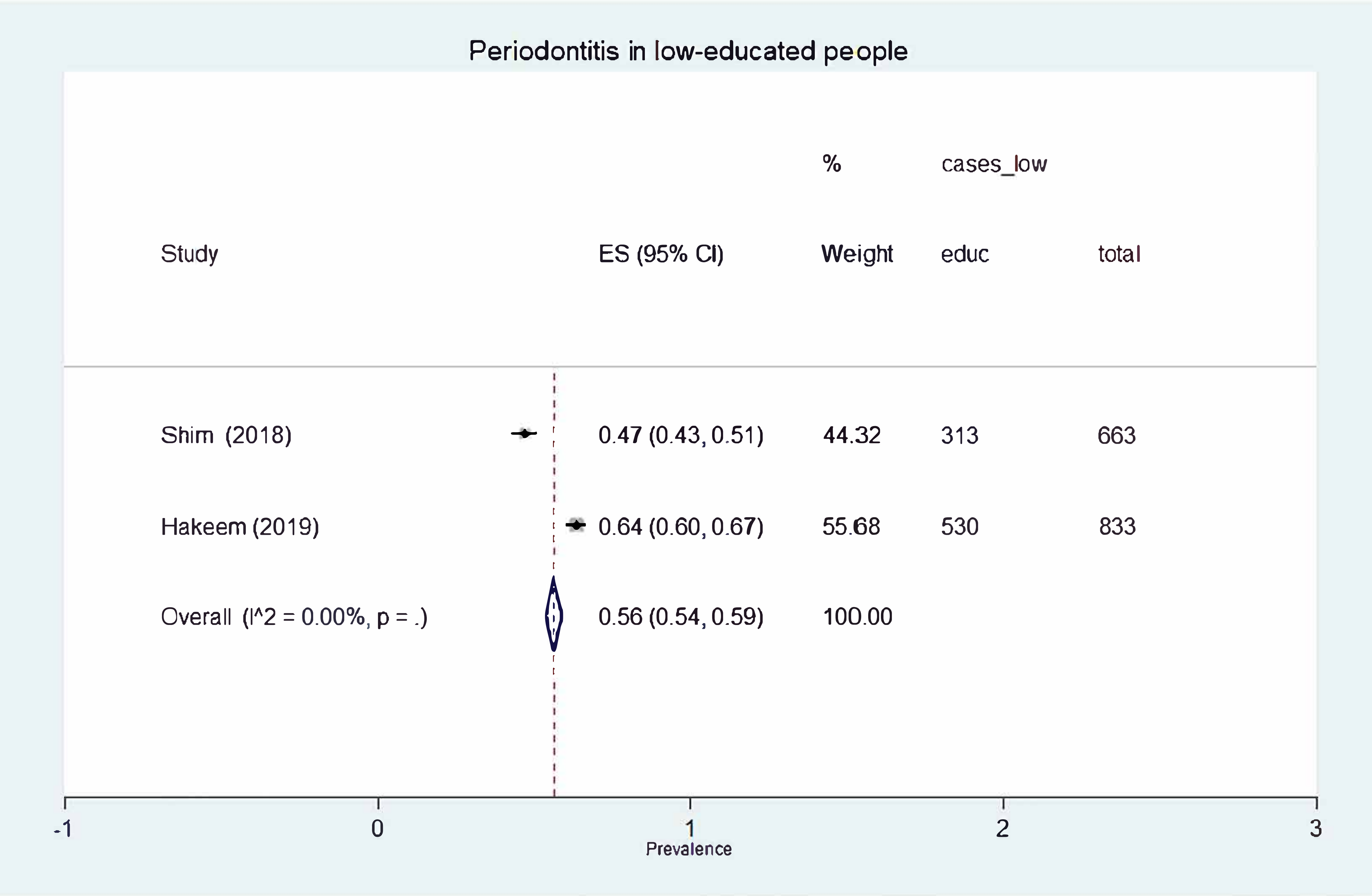
Periodontitis in low-educated people
Study
ES (95% CI)
% Weight
cases_low educ
total
Shim (2018)
0.47 (0.43, 0.51)
44.32
313
663
Hakeem (2019)
0.64 (0.60, 0.67)
55.68
530
833
Overall (I^2 = 0.00%, p = .)
0.56 (0.54, 0.59)
100.00
-1
0
1
2
3
Prevalence

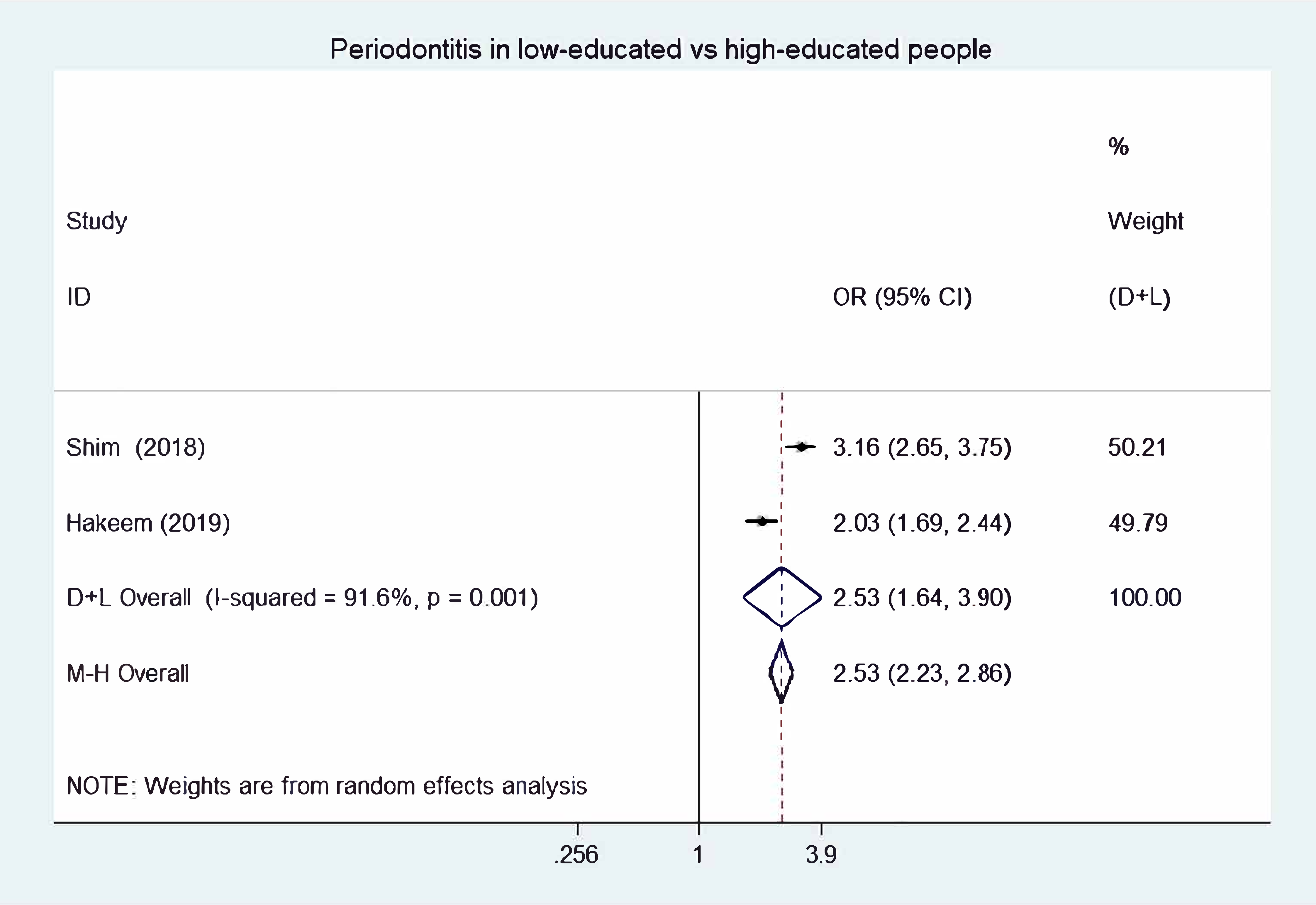

Periodontitis in low-educated vs high-educated people
Study
ID
OR (95% CI)
%
Weight
(D+L)
Shim (2018)
3.16 (2.65, 3.75)
50.21
Hakeem (2019)
2.03 (1.69, 2.44)
49.79
D+L Overall (I-squared = 91.6%, p = 0.001)
2.53 (1.64, 3.90)
100.00
M-H Overall
2.53 (2.23, 2.86)
NOTE: Weights are from random effects analysis
.256
1
3.9

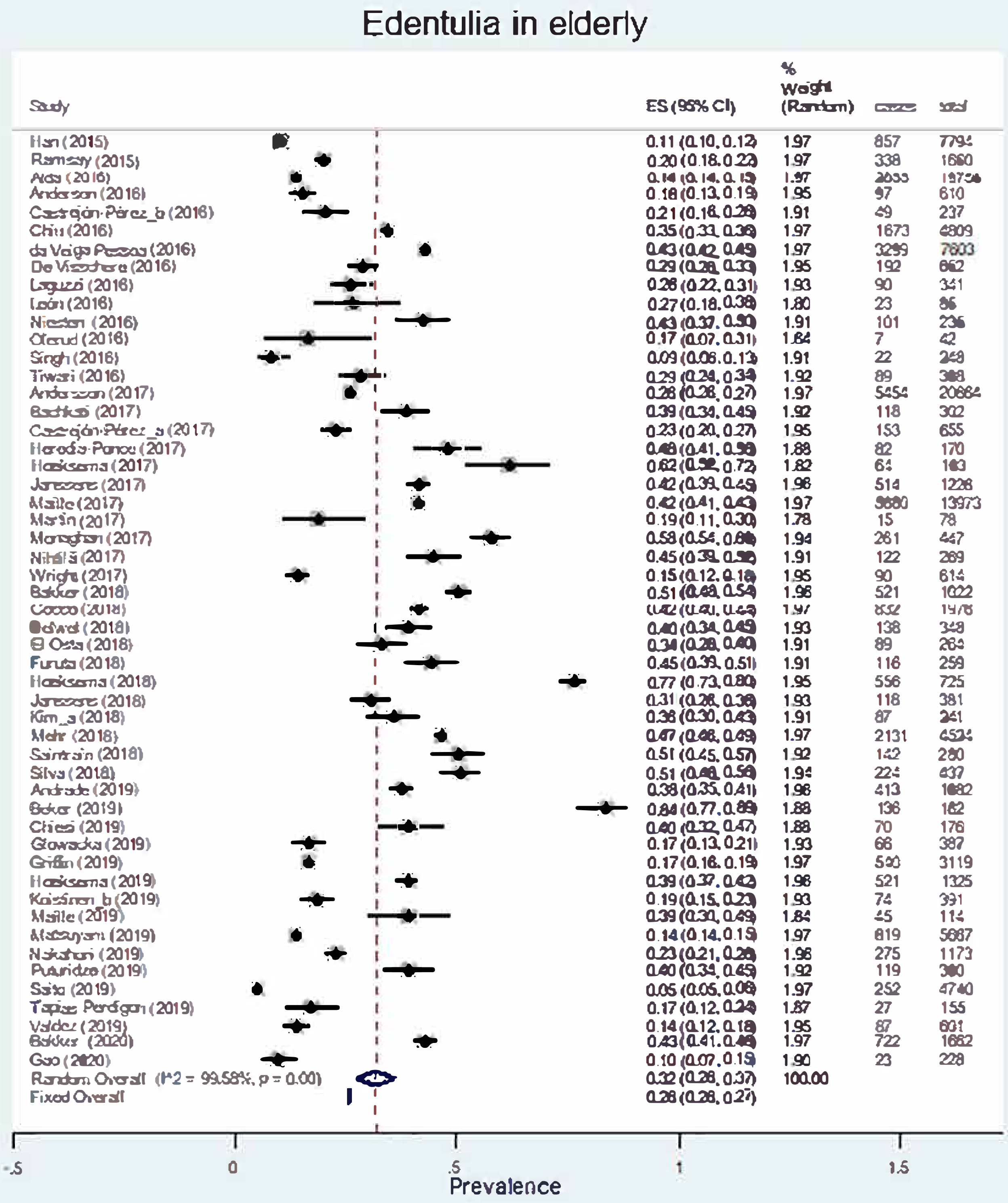

Edentulia in elderly
Study
ES (95% CI)
% Weight (Random)
cases
total
Han (2015) 0.11 (0.10, 0.12) 1.97 857 7794
Ramsay (2015) 0.20 (0.18, 0.22) 1.97 338 1660
Anderson (2016) 0.16 (0.13, 0.19) 1.95 97 610
Castrejón-Pérez_b (2016) 0.21 (0.16, 0.26) 1.91 49 237
Chiu (2016) 0.35 (0.33, 0.36) 1.97 1673 4809
da Veiga Pessoa (2016) 0.43 (0.42, 0.45) 1.97 3299 7603
De Visschere (2016) 0.29 (0.26, 0.33) 1.95 192 662
León (2016) 0.27 (0.18, 0.38) 1.80 23 86
Singh (2016) 0.09 (0.06, 0.13) 1.91 22 249
Tiwari (2016) 0.29 (0.24, 0.34) 1.92 89 308
Anderson (2017) 0.26 (0.26, 0.27) 1.97 5454 20684
Castrejón-Pérez_a (2017) 0.23 (0.20, 0.27) 1.95 153 655
Hoeksema (2017) 0.62 (0.52, 0.72) 1.82 64 103
Maille (2017) 0.42 (0.41, 0.43) 1.97 5880 13973
Martin (2017) 0.19 (0.11, 0.30) 1.78 15 78
Monaghan (2017) 0.58 (0.54, 0.63) 1.94 261 447
Wright (2017) 0.15 (0.12, 0.18) 1.95 90 614
Bakker (2018) 0.51 (0.48, 0.54) 1.96 521 1022
El Osta (2018) 0.34 (0.28, 0.40) 1.91 89 264
Furuta (2018) 0.45 (0.39, 0.51) 1.91 116 259
Hoeksema (2018) 0.77 (0.73, 0.80) 1.95 556 725
Silva (2018) 0.51 (0.46, 0.56) 1.94 224 437
Andrade (2019) 0.38 (0.35, 0.41) 1.96 413 1082
Griffin (2019) 0.17 (0.16, 0.19) 1.97 3119
Hoeksema (2019) 0.39 (0.37, 0.42) 1.96 521 1325
Maille (2019) 0.39 (0.30, 0.49) 1.84 45 114
Saito (2019) 0.05 (0.05, 0.06) 1.97 252 4740
Valdez (2019) 0.14 (0.12, 0.18) 1.95 87 601
Bakker (2020) 0.43 (0.41, 0.46) 1.97 722 1662
Random Overall (I^2 = 99.58%, p = 0.00) 0.32 (0.28, 0.37) 100.00
Fixed Overall
-.5
0
.5
1
1.5
Prevalence

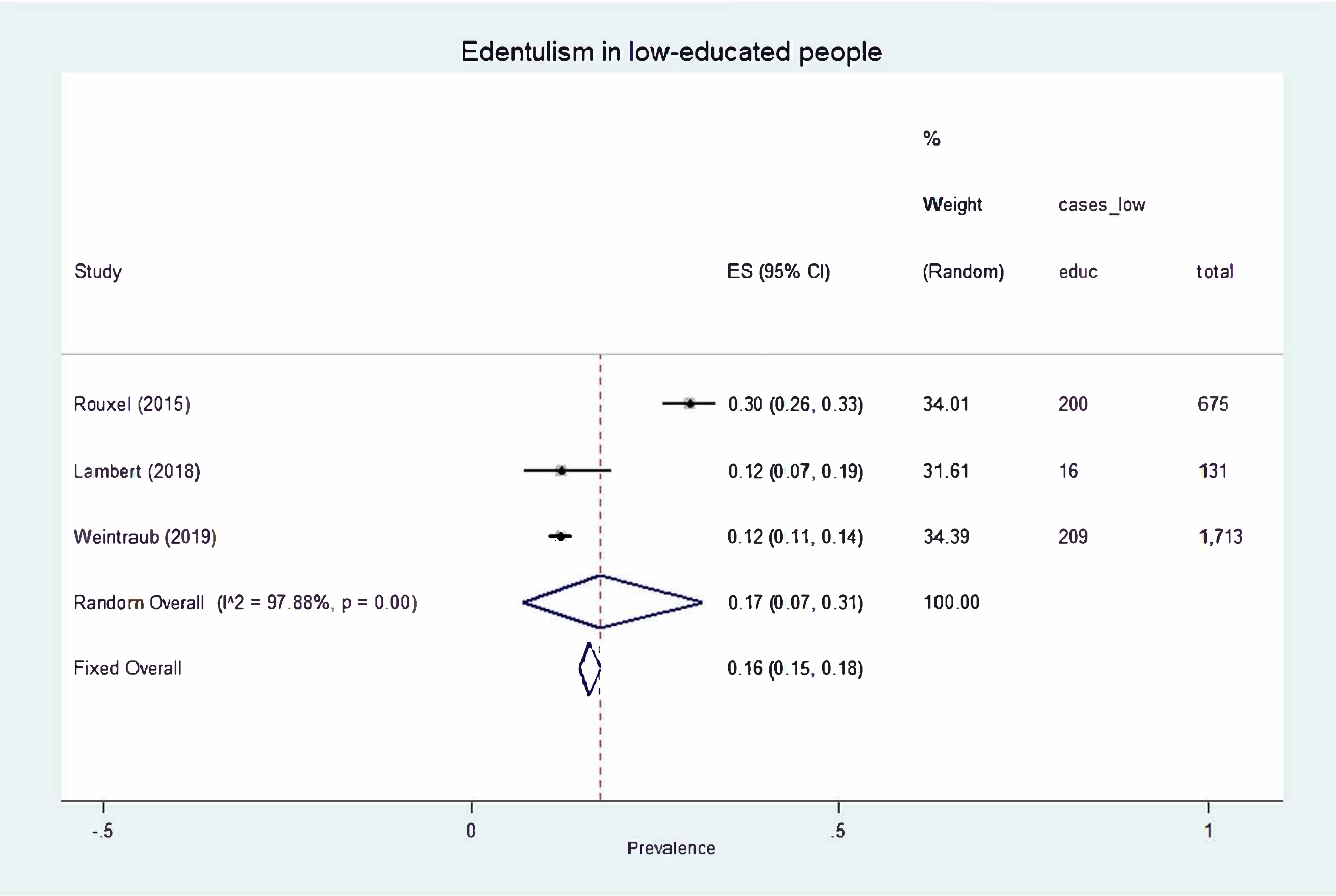
Edentulism in low-educated people
Study
ES (95% CI)
% Weight (Random)
cases_low educ
total
Rouxel (2015) 0.30 (0.26, 0.33) 34.01 200 675
Lambert (2018) 0.12 (0.07, 0.19) 31.61 16 131
Weintraub (2019) 0.12 (0.11, 0.14) 34.39 209 1,713
Random Overall (I^2 = 97.88%, p = 0.00) 0.17 (0.07, 0.31) 100.00
Fixed Overall 0.16 (0.15, 0.18)
-.5
0
.5
1
Prevalence

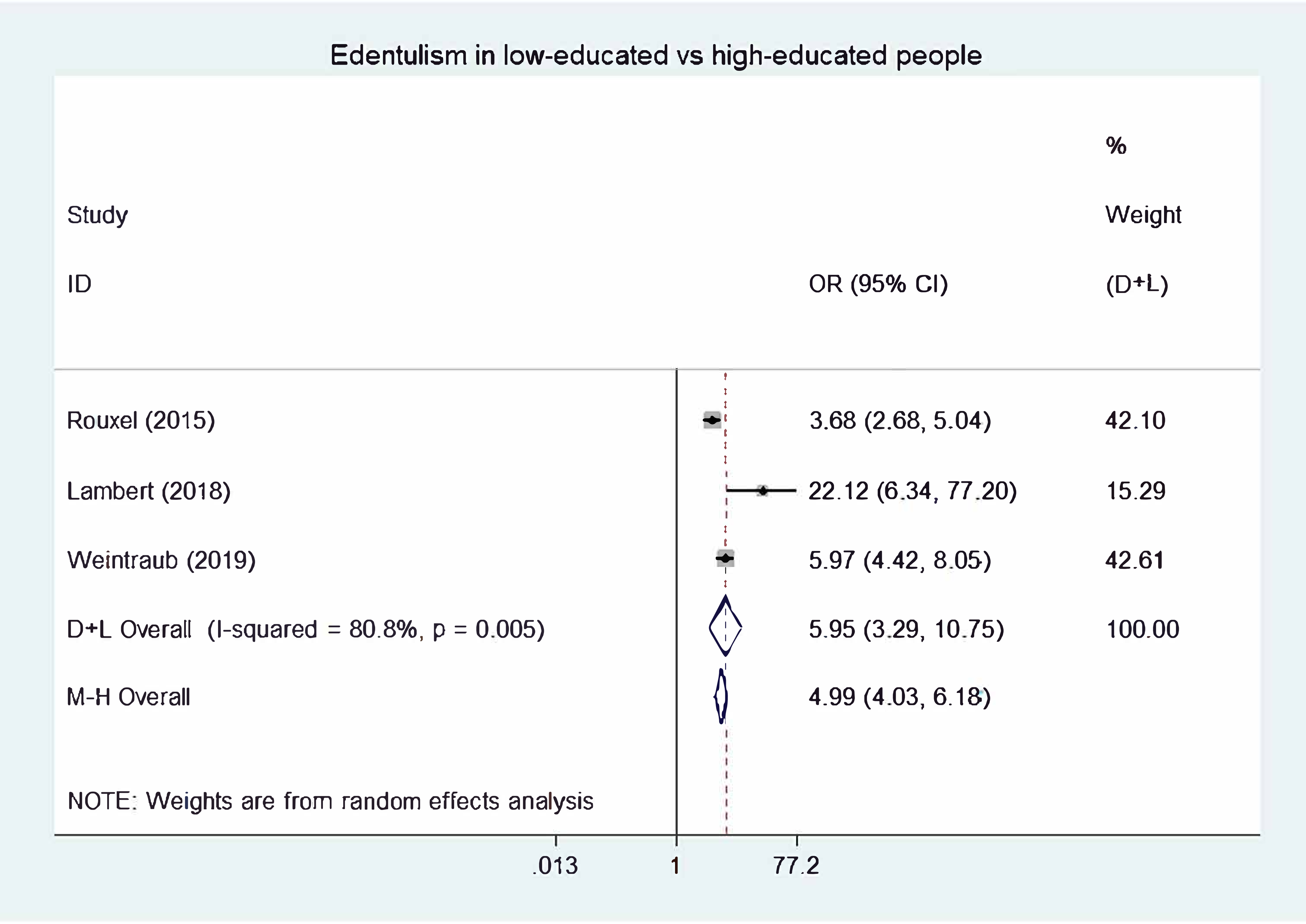

Edentulism in low-educated vs high-educated people
%
Study
Weight
ID
OR (95% CI)
(D+L)
Rouxel (2015)
3.68 (2.68, 5.04)
42.10
Lambert (2018)
22.12 (6.34, 77.20)
15.29
Weintraub (2019)
5.97 (4.42, 8.05)
42.61
D+L Overall (I-squared = 80.8%, p = 0.005)
5.95 (3.29, 10.75)
100.00
M-H Overall
4.99 (4.03, 6.18)
NOTE: Weights are from random effects analysis
.013
1
77.2

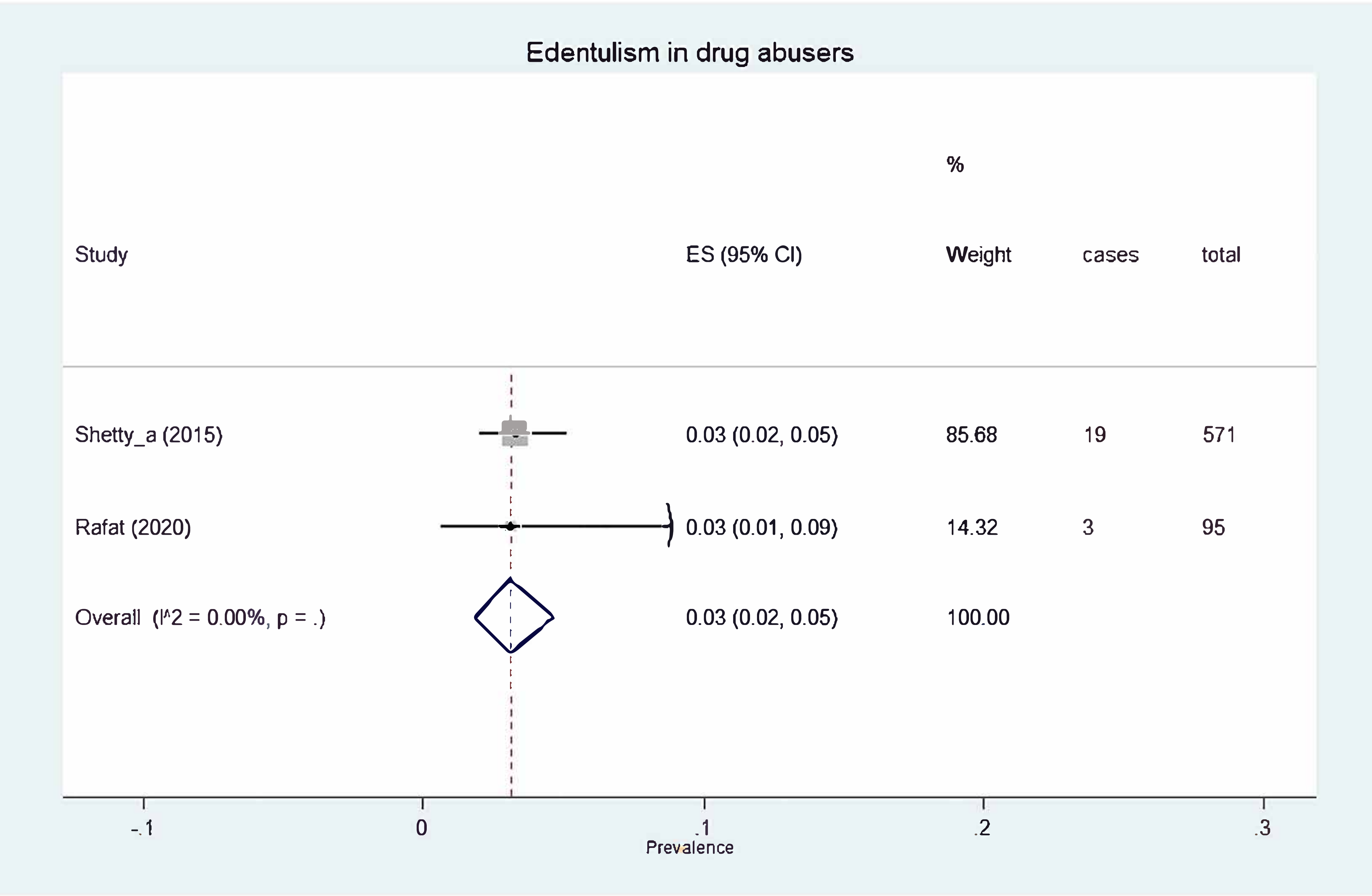

Edentulism in drug abusers
%
Study
ES (95% CI)
Weight
cases
total
Shetty_a (2015)
0.03 (0.02, 0.05)
85.68
19
571
Rafat (2020)
0.03 (0.01, 0.09)
14.32
3
95
Overall (I^2 = 0.00%, p = .)
0.03 (0.02, 0.05)
100.00
-.1
0
.1
.2
.3
Prevalence

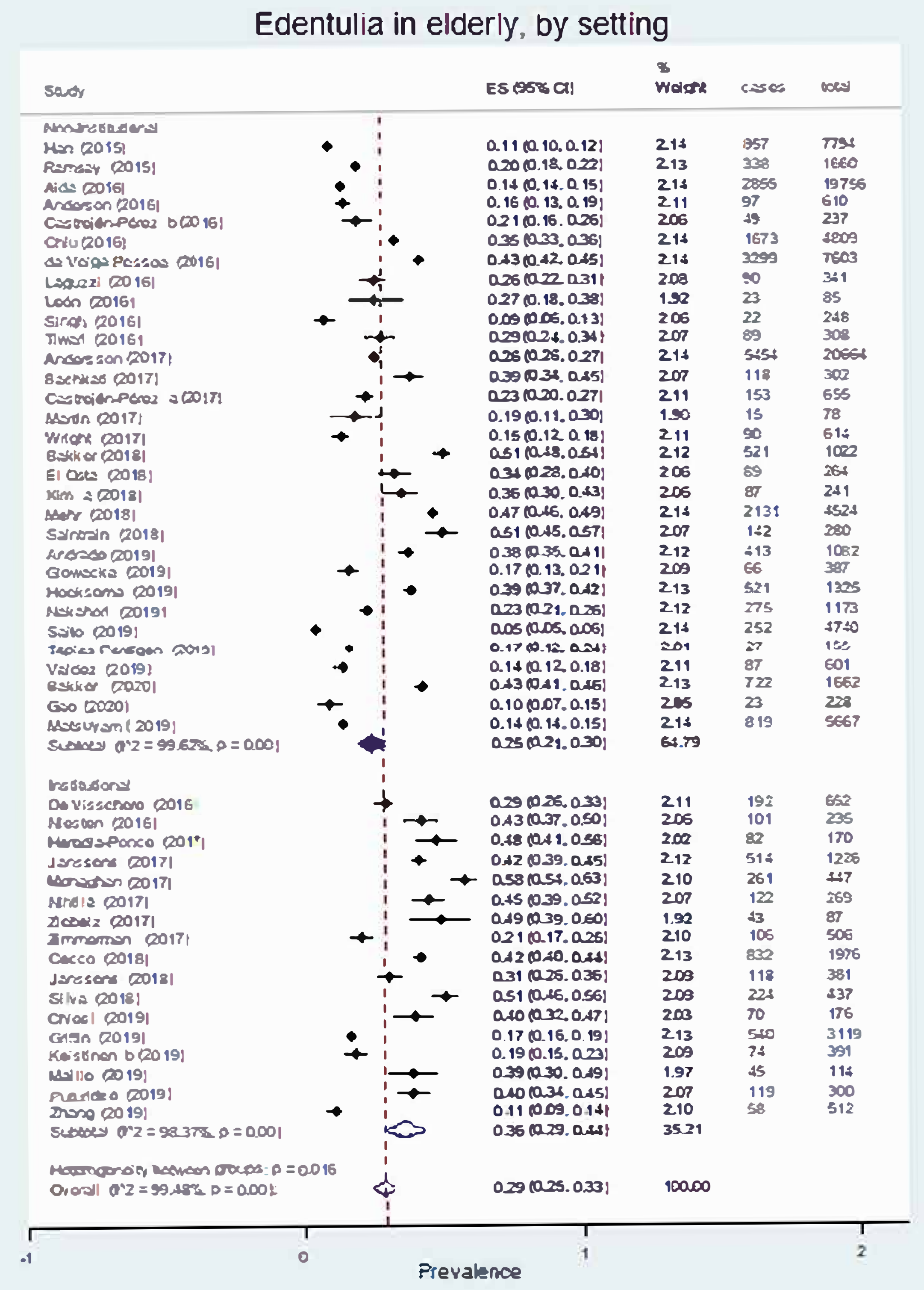
Edentulia in elderly, by setting
Study
ES (95% CI)
% Weight
cases
total
Noninstitutional
Han (2015) 0.11 (0.10, 0.12) 2.14 857 7794
Ramsay (2015) 0.20 (0.18, 0.22) 2.13 338 1660
Aida (2016) 0.14 (0.14, 0.15) 2.14 2856 19756
Anderson (2016) 0.16 (0.13, 0.19) 2.11 97 610
Castrejón-Pérez b (2016) 0.21 (0.16, 0.26) 2.06 49 237
Chiu (2016) 0.35 (0.33, 0.36) 2.14 1673 4809
da Veiga Pessoa (2016) 0.43 (0.42, 0.45) 2.14 3299 7603
Laguzzi (2016) 0.26 (0.22, 0.31) 2.08 90 341
León (2016) 0.27 (0.18, 0.38) 1.92 23 85
Singh (2016) 0.09 (0.06, 0.13) 2.06 22 248
Tiwari (2016) 0.29 (0.24, 0.34) 2.07 89 308
Andersson (2017) 0.26 (0.26, 0.27) 2.14 5454 20664
Bachkati (2017) 0.39 (0.34, 0.45) 2.07 118 302
Castrejón-Pérez a (2017) 0.23 (0.20, 0.27) 2.11 153 656
Martin (2017) 0.19 (0.11, 0.30) 1.90 15 78
Wright (2017) 0.15 (0.12, 0.18) 2.11 90 614
Bakker (2018) 0.51 (0.48, 0.54) 2.12 521 1022
El Osta (2018) 0.34 (0.28, 0.40) 2.06 69 264
Kim a (2018) 0.36 (0.30, 0.43) 2.06 87 241
Mehr (2018) 0.47 (0.46, 0.49) 2.14 2131 4524
Saintrain (2018) 0.51 (0.45, 0.57) 2.07 142 280
Andrade (2019) 0.38 (0.36, 0.41) 2.12 413 1082
Glowacka (2019) 0.17 (0.13, 0.21) 2.09 66 387
Hoeksema (2019) 0.39 (0.37, 0.42) 2.13 521 1325
Nakshad (2019) 0.23 (0.21, 0.26) 2.12 275 1173
Saito (2019) 0.05 (0.05, 0.06) 2.14 252 4740
0.17 (0.12, 0.24) 2.01 27
Valdez (2019) 0.14 (0.12, 0.18) 2.11 87 601
Bakker (2020) 0.43 (0.41, 0.46) 2.13 722 1662
Gao (2020) 0.10 (0.07, 0.15) 2.05 23 228
Mattsuyama (2019) 0.14 (0.14, 0.15) 2.14 819 5667
Subtotal (I^2 = 99.62%, p = 0.00) 0.25 (0.21, 0.30) 64.79
Institutional
De Visschere (2016) 0.29 (0.26, 0.33) 2.11 192 652
Niesten (2016) 0.43 (0.37, 0.50) 2.06 101 235
Heredia-Ponce (2017) 0.48 (0.41, 0.56) 2.02 82 170
Janssens (2017) 0.42 (0.39, 0.45) 2.12 514 1226
Monaghan (2017) 0.58 (0.54, 0.63) 2.10 261 447
Nihtila (2017) 0.45 (0.39, 0.52) 2.07 122 269
Ziebolz (2017) 0.49 (0.39, 0.60) 1.92 43 87
Zimmerman (2017) 0.21 (0.17, 0.26) 2.10 106 506
Cocco (2018) 0.42 (0.40, 0.44) 2.13 832 1976
Janssens (2018) 0.31 (0.26, 0.36) 2.08 118 381
Silva (2018) 0.51 (0.46, 0.56) 2.08 224 437
Chiesi (2019) 0.40 (0.32, 0.47) 2.03 70 176
Gilsin (2019) 0.17 (0.16, 0.19) 2.13 540 3119
Keistinen b (2019) 0.19 (0.15, 0.23) 2.09 74 391
Malilo (2019) 0.39 (0.30, 0.49) 1.97 45 114
Pueyrredo (2019) 0.40 (0.34, 0.45) 2.07 119 300
Zhang (2019) 0.11 (0.09, 0.14) 2.10 58 512
Subtotal (I^2 = 98.37%, p = 0.00) 0.36 (0.29, 0.44) 35.21
Heterogeneity between groups: p = 0.016
Overall (I^2 = 99.48%, p = 0.00); 0.29 (0.25, 0.33) 100.00
-1
0
1
2
Prevalence

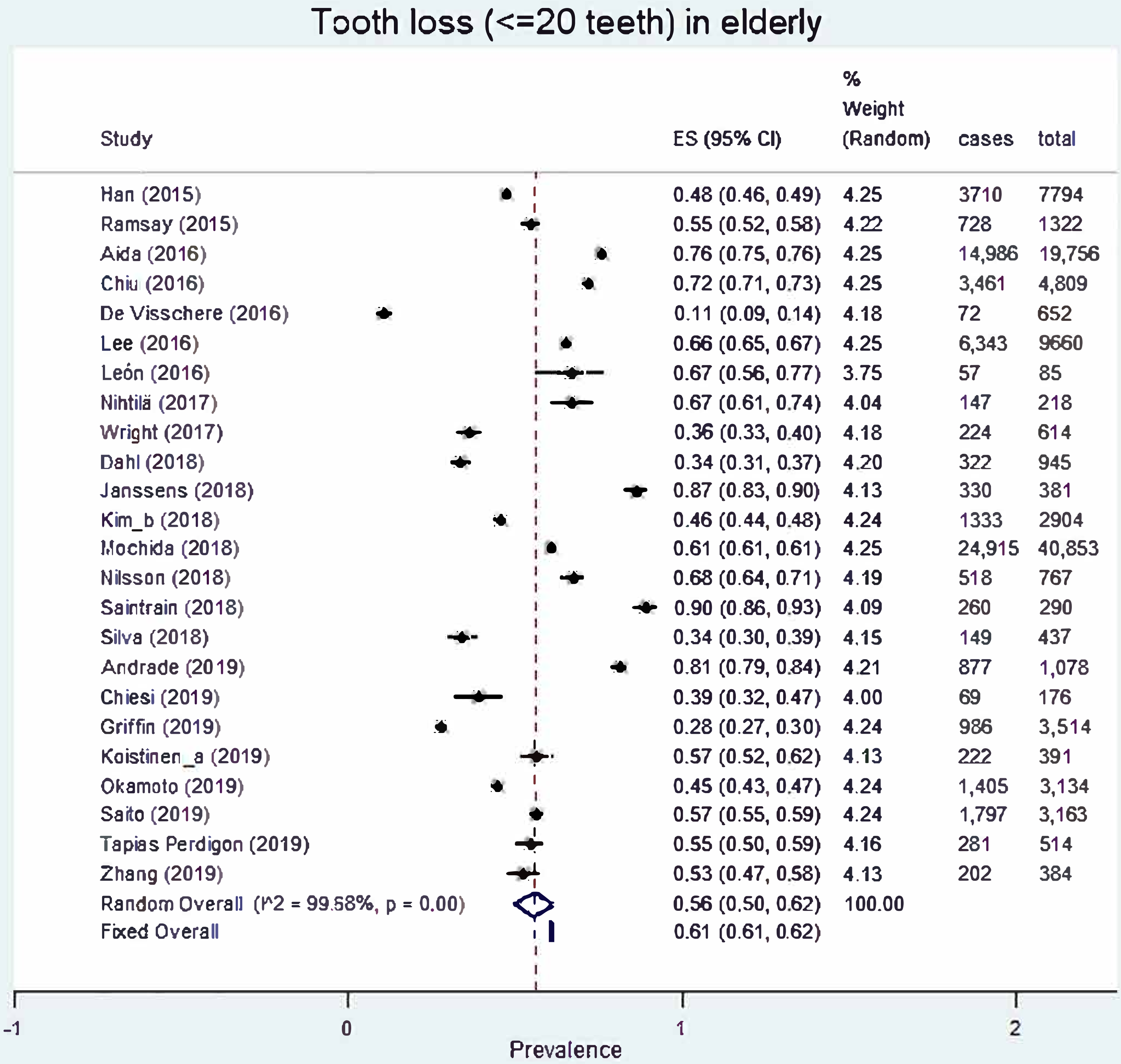
Tooth loss (<=20 teeth) in elderly
Study
ES (95% CI)
% Weight (Random)
cases
total
Han (2015) 0.48 (0.46, 0.49) 4.25 3710 7794
Ramsay (2015) 0.55 (0.52, 0.58) 4.22 728 1322
Aida (2016) 0.76 (0.75, 0.76) 4.25 14,986 19,756
Chiu (2016) 0.72 (0.71, 0.73) 4.25 3,461 4,809
De Visschere (2016) 0.11 (0.09, 0.14) 4.18 72 652
Lee (2016) 0.66 (0.65, 0.67) 4.25 6,343 9660
León (2016) 0.67 (0.56, 0.77) 3.75 57 85
Nihtilä (2017) 0.67 (0.61, 0.74) 4.04 147 218
Wright (2017) 0.36 (0.33, 0.40) 4.18 224 614
Dahl (2018) 0.34 (0.31, 0.37) 4.20 322 945
Janssens (2018) 0.87 (0.83, 0.90) 4.13 330 381
Kim_b (2018) 0.46 (0.44, 0.48) 4.24 1333 2904
Mochida (2018) 0.61 (0.61, 0.61) 4.25 24,915 40,853
Nilsson (2018) 0.68 (0.64, 0.71) 4.19 518 767
Saintrain (2018) 0.90 (0.86, 0.93) 4.09 260 290
Silva (2018) 0.34 (0.30, 0.39) 4.15 149 437
Andrade (2019) 0.81 (0.79, 0.84) 4.21 877 1,078
Chiesi (2019) 0.39 (0.32, 0.47) 4.00 69 176
Griffin (2019) 0.28 (0.27, 0.30) 4.24 986 3,514
Koistinen_a (2019) 0.57 (0.52, 0.62) 4.13 222 391
Okamoto (2019) 0.45 (0.43, 0.47) 4.24 1,405 3,134
Saito (2019) 0.57 (0.55, 0.59) 4.24 1,797 3,163
Tapias Perdigon (2019) 0.55 (0.50, 0.59) 4.16 281 514
Zhang (2019) 0.53 (0.47, 0.58) 4.13 202 384
Random Overall (I^2 = 99.68%, p = 0.00) 0.56 (0.50, 0.62) 100.00
Fixed Overall 0.61 (0.61, 0.62)
-1
0
1
2
Prevalence

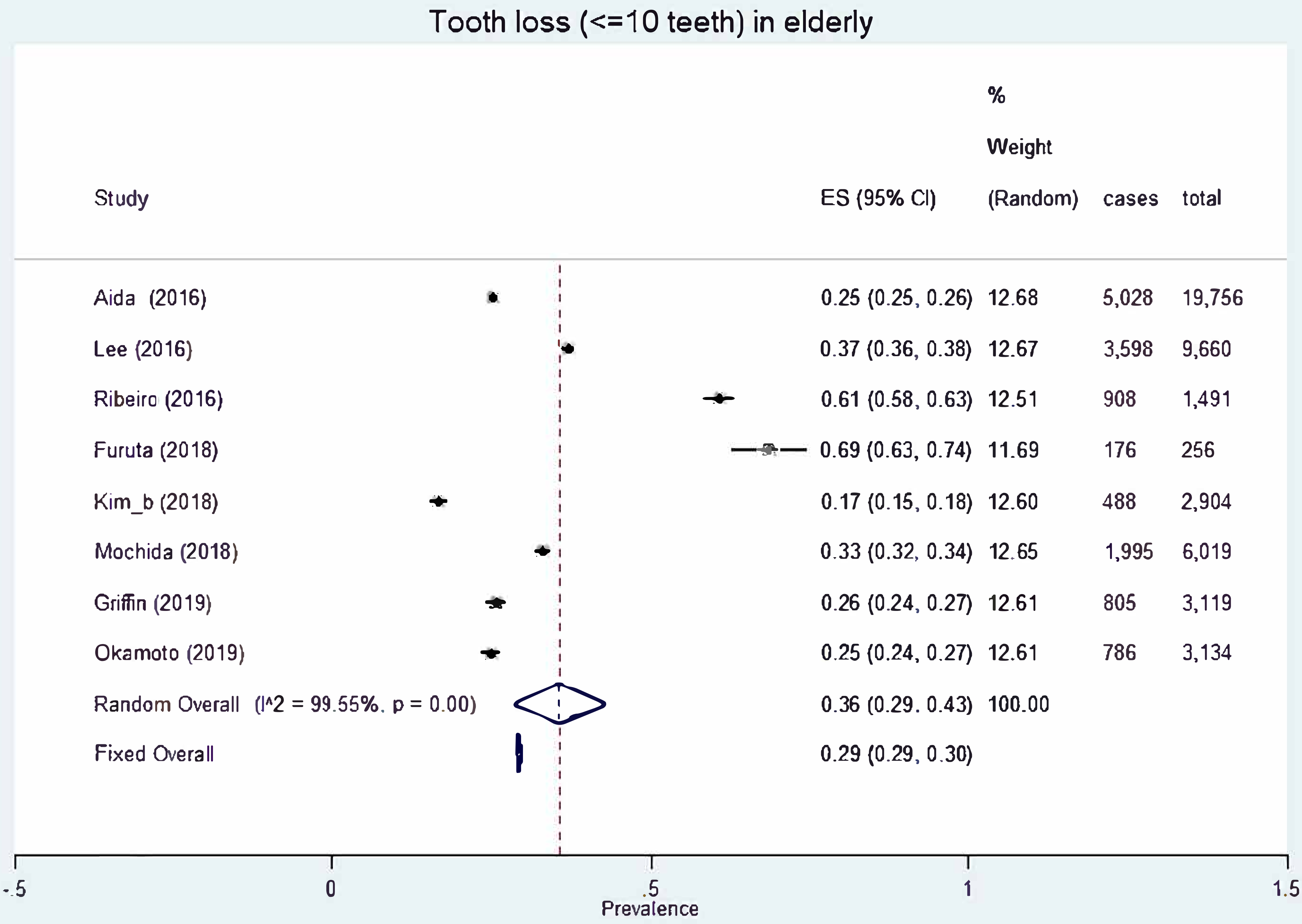

Tooth loss (<=10 teeth) in elderly
Study
ES (95% CI)
% Weight (Random)
cases
total
Aida (2016) 0.25 (0.25, 0.26) 12.68 5,028 19,756
Lee (2016) 0.37 (0.36, 0.38) 12.67 3,598 9,660
Ribeiro (2016) 0.61 (0.58, 0.63) 12.51 908 1,491
Furuta (2018) 0.69 (0.63, 0.74) 11.69 176 256
Kim_b (2018) 0.17 (0.15, 0.18) 12.60 488 2,904
Mochida (2018) 0.33 (0.32, 0.34) 12.65 1,995 6,019
Griffin (2019) 0.26 (0.24, 0.27) 12.61 805 3,119
Okamoto (2019) 0.25 (0.24, 0.27) 12.61 786 3,134
Random Overall (I^2 = 99.55%, p = 0.00) 0.36 (0.29, 0.43) 100.00
Fixed Overall 0.29 (0.29, 0.30)
-.5
0
.5
1
1.5
Prevalence

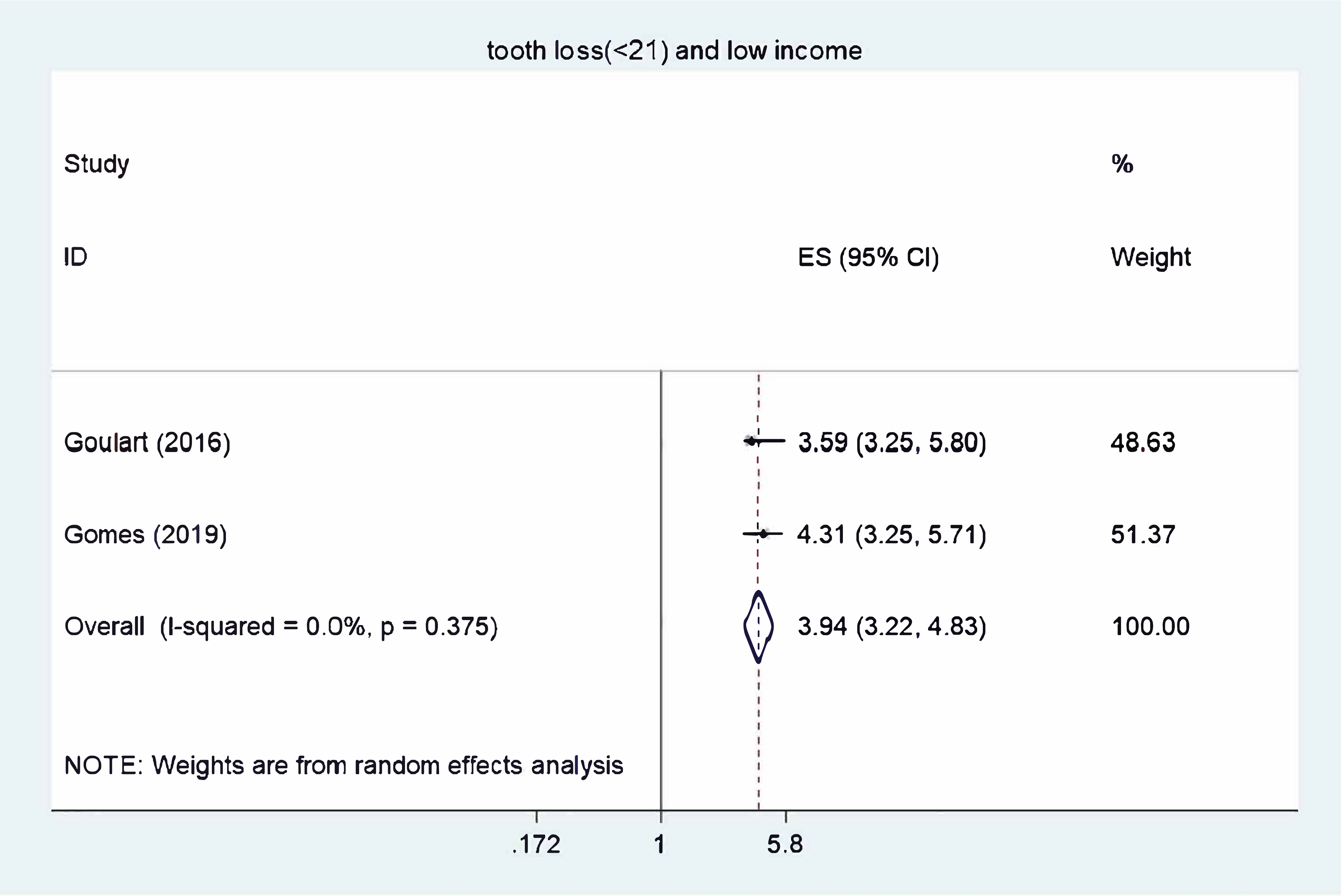
tooth loss(<21) and low income
Study
ID
ES (95% CI)
%
Weight
Goulart (2016)
3.59 (3.25, 5.80)
48.63
Gomes (2019)
4.31 (3.25, 5.71)
51.37
Overall (I-squared = 0.0%, p = 0.375)
3.94 (3.22, 4.83)
100.00
NOTE: Weights are from random effects analysis
.172
1
5.8

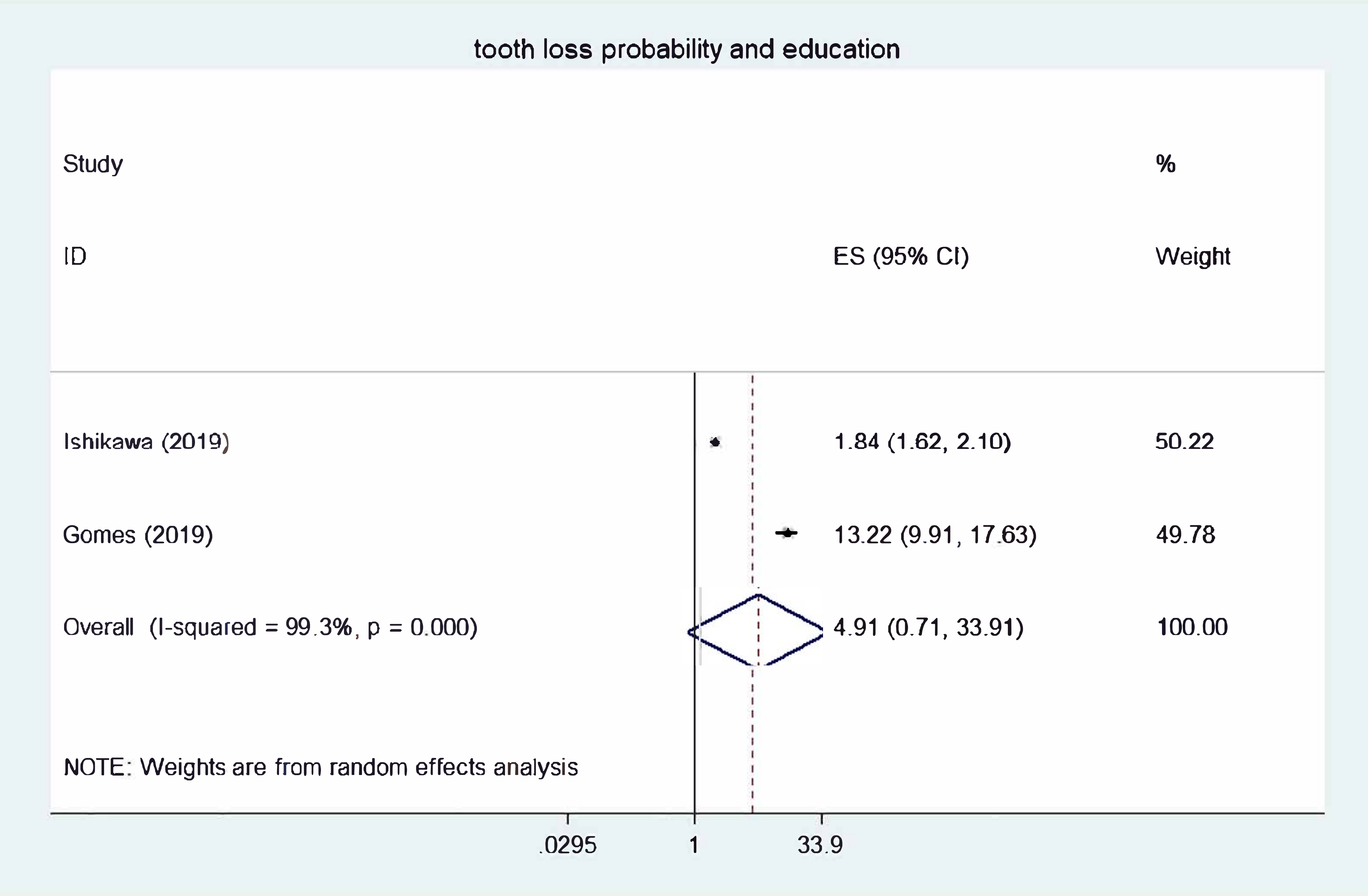
tooth loss probability and education
Study
ID
ES (95% CI)
%
Weight
Ishikawa (2019)
1.84 (1.62, 2.10)
50.22
Gomes (2019)
13.22 (9.91, 17.63)
49.78
Overall (I-squared = 99.3%, p = 0.000)
4.91 (0.71, 33.91)
100.00
NOTE: Weights are from random effects analysis
.0295
1
33.9

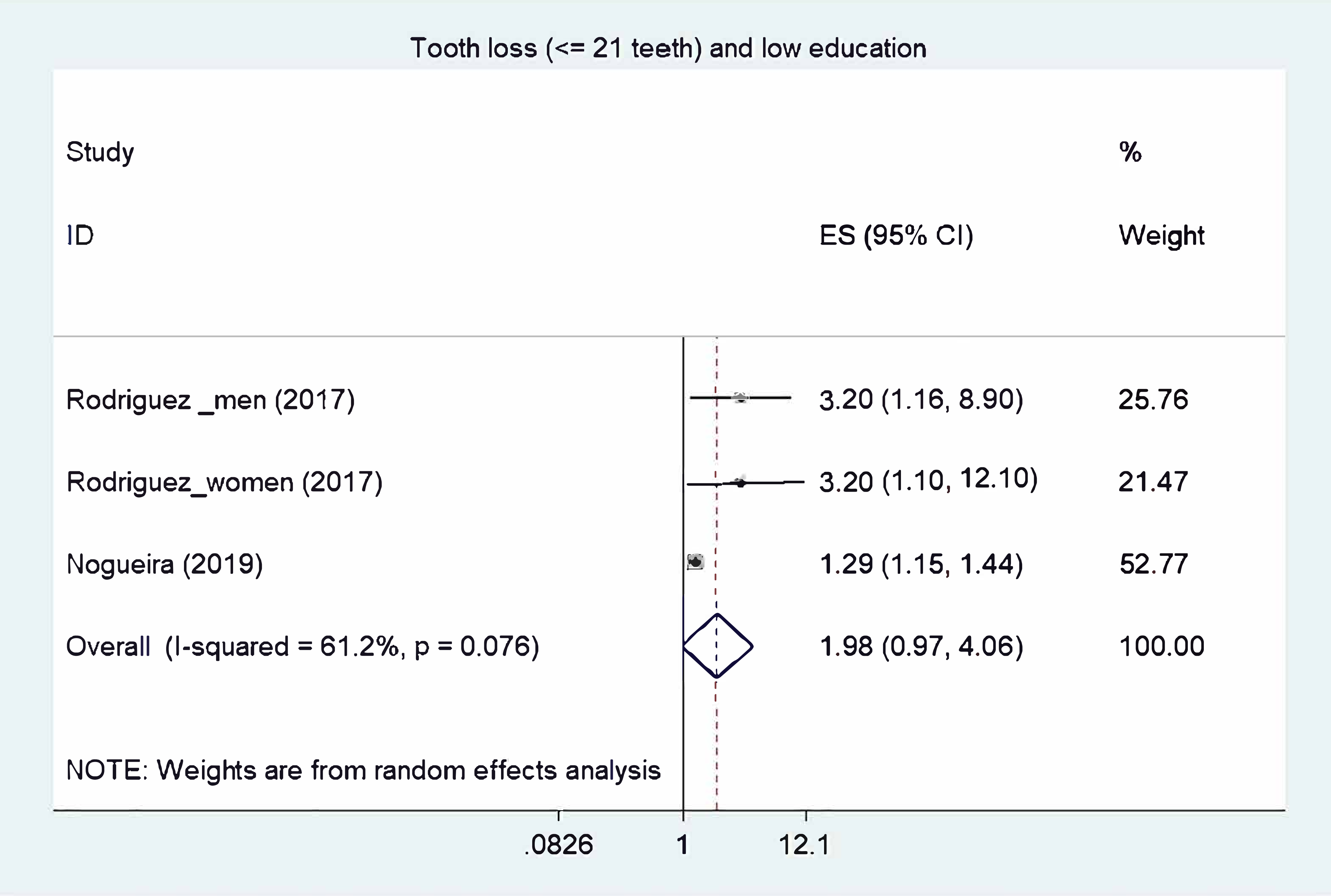

Tooth loss (<= 21 teeth) and low education
Study
%
ID
ES (95% CI)
Weight
Rodriguez _men (2017)
3.20 (1.16, 8.90)
25.76
Rodriguez_women (2017)
3.20 (1.10, 12.10)
21.47
Nogueira (2019)
1.29 (1.15, 1.44)
52.77
Overall (I-squared = 61.2%, p = 0.076)
1.98 (0.97, 4.06)
100.00
NOTE: Weights are from random effects analysis
.0826
1
12.1

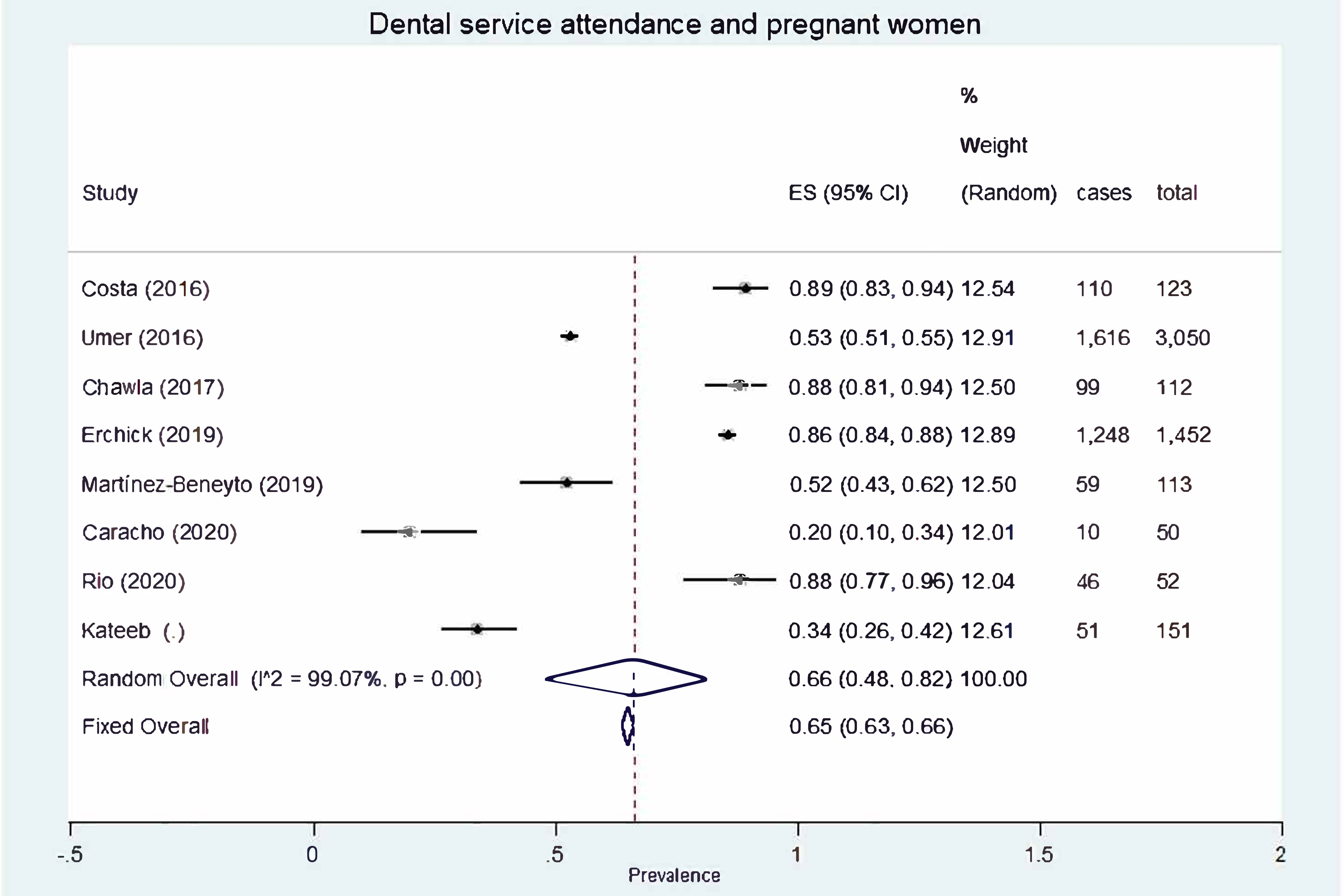
Dental service attendance and pregnant women
%
Weight
Study
ES (95% CI)
(Random)
cases
total
Costa (2016)
0.89 (0.83, 0.94) 12.54
110
123
Umer (2016)
0.53 (0.51, 0.55) 12.91
1,616
3,050
Chawla (2017)
0.88 (0.81, 0.94) 12.50
99
112
Erchick (2019)
0.86 (0.84, 0.88) 12.89
1,248
1,452
Martínez-Beneyto (2019)
0.52 (0.43, 0.62) 12.50
59
113
Caracho (2020)
0.20 (0.10, 0.34) 12.01
10
50
Rio (2020)
0.88 (0.77, 0.96) 12.04
46
52
Kateeb (.)
0.34 (0.26, 0.42) 12.61
51
151
Random Overall (I^2 = 99.07%, p = 0.00)
0.66 (0.48, 0.82) 100.00
Fixed Overall
0.65 (0.63, 0.66)
-.5
0
.5
1
1.5
2
Prevalence

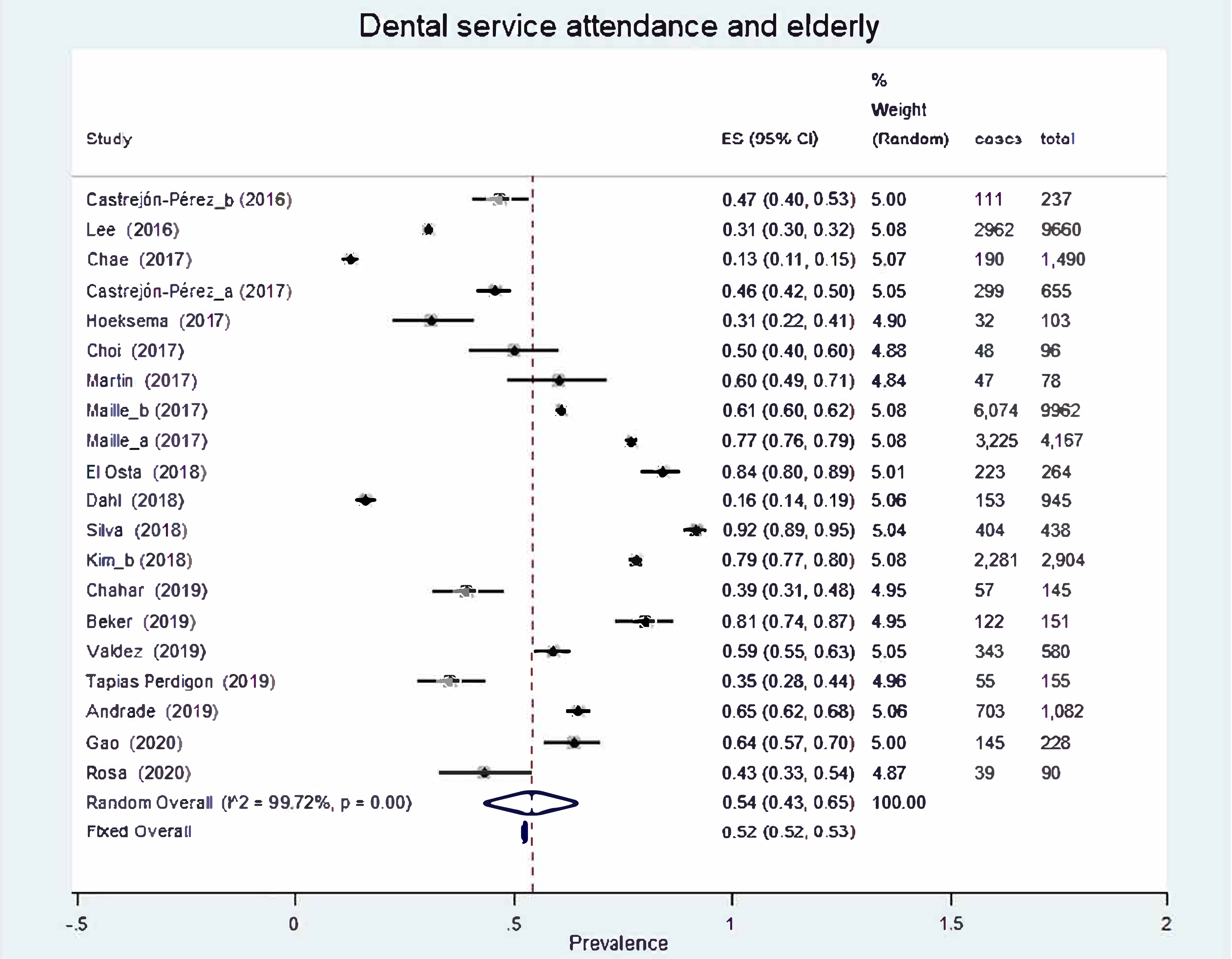
Dental service attendance and elderly
Study
ES (95% CI)
% Weight (Random)
cases
total
Castrejón-Pérez_b (2016) 0.47 (0.40, 0.53) 5.00 111 237
Lee (2016) 0.31 (0.30, 0.32) 5.08 2962 9660
Chae (2017) 0.13 (0.11, 0.15) 5.07 190 1,490
Castrejón-Pérez_a (2017) 0.46 (0.42, 0.50) 5.05 299 655
Hoeksema (2017) 0.31 (0.22, 0.41) 4.90 32 103
Choi (2017) 0.50 (0.40, 0.60) 4.88 48 96
Martin (2017) 0.60 (0.49, 0.71) 4.84 47 78
Maille_b (2017) 0.61 (0.60, 0.62) 5.08 6,074 9962
Maille_a (2017) 0.77 (0.76, 0.79) 5.08 3,225 4,167
El Osta (2018) 0.84 (0.80, 0.89) 5.01 223 264
Dahl (2018) 0.16 (0.14, 0.19) 5.06 153 945
Silva (2018) 0.92 (0.89, 0.95) 5.04 404 438
Kim_b (2018) 0.79 (0.77, 0.80) 5.08 2,281 2,904
Chahar (2019) 0.39 (0.31, 0.48) 4.95 57 145
Beker (2019) 0.81 (0.74, 0.87) 4.95 122 151
Valdez (2019) 0.59 (0.55, 0.63) 5.05 343 580
Tapias Perdigon (2019) 0.35 (0.28, 0.44) 4.96 55 155
Andrade (2019) 0.65 (0.62, 0.68) 5.06 703 1,082
Gao (2020) 0.64 (0.57, 0.70) 5.00 145 228
Rosa (2020) 0.43 (0.33, 0.54) 4.87 39 90
Random Overall (I^2 = 99.72%, p = 0.00) 0.54 (0.43, 0.65) 100.00
Fixed Overall 0.52 (0.52, 0.53)
-.5
0
.5
1
1.5
2
Prevalence

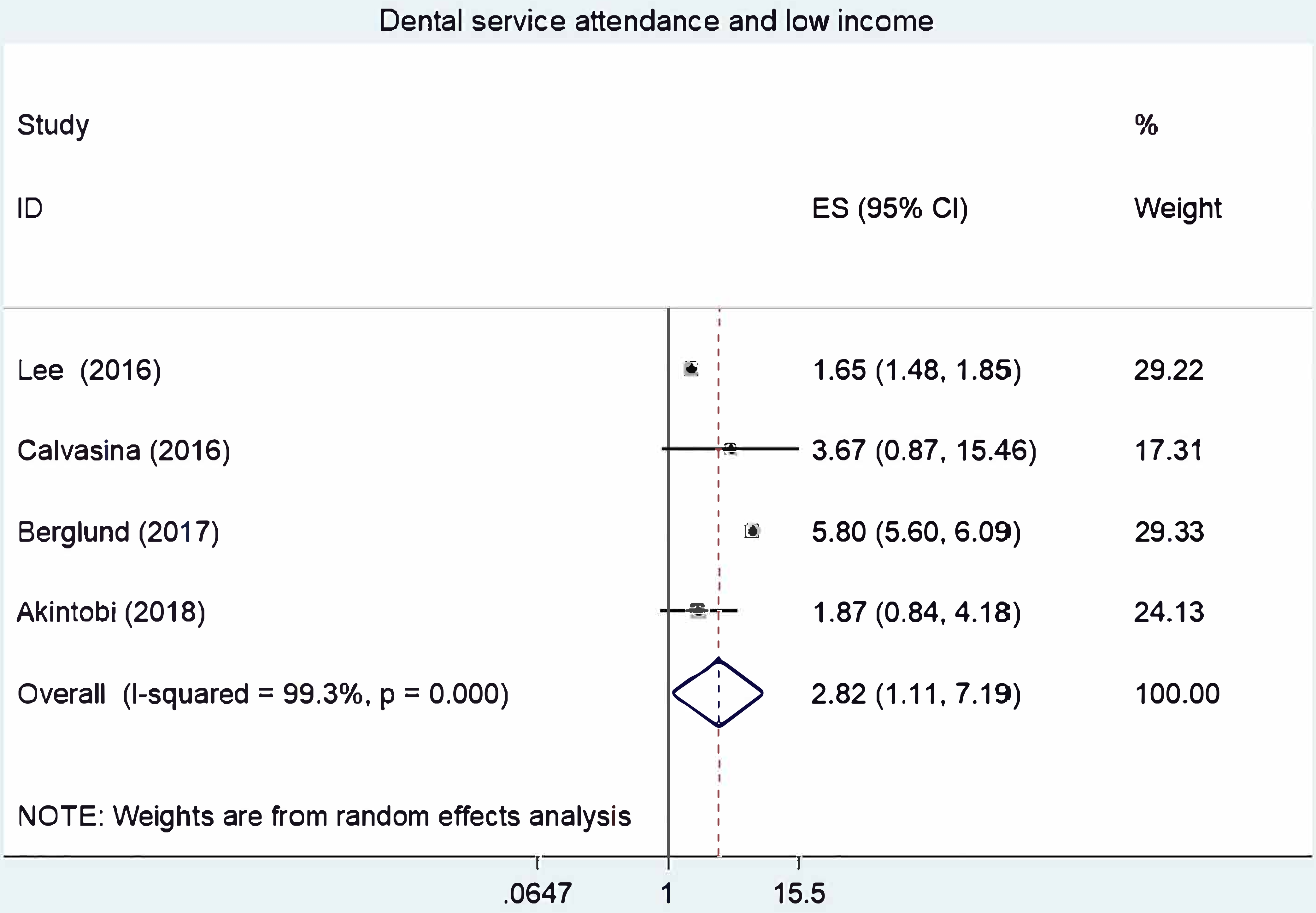

Dental service attendance and low income
Study
ID
ES (95% CI)
%
Weight
Lee (2016)
1.65 (1.48, 1.85)
29.22
Calvasina (2016)
3.67 (0.87, 15.46)
17.31
Berglund (2017)
5.80 (5.60, 6.09)
29.33
Akintobi (2018)
1.87 (0.84, 4.18)
24.13
Overall (I-squared = 99.3%, p = 0.000)
2.82 (1.11, 7.19)
100.00
NOTE: Weights are from random effects analysis
.0647
1
15.5

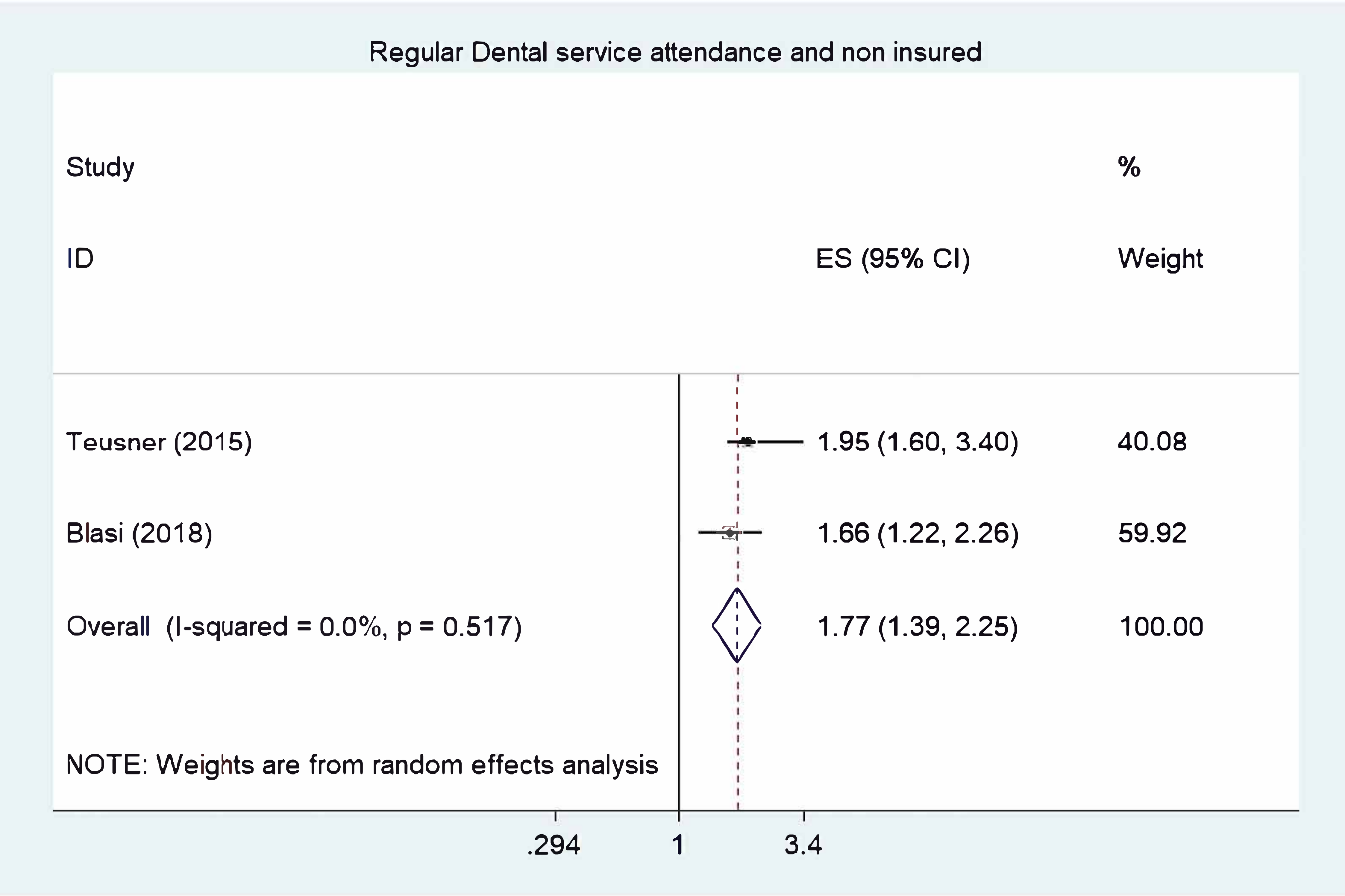
Regular Dental service attendance and non insured
Study
ID
ES (95% CI)
%
Weight
Teusner (2015)
1.95 (1.60, 3.40)
40.08
Blasi (2018)
1.66 (1.22, 2.26)
59.92
Overall (I-squared = 0.0%, p = 0.517)
1.77 (1.39, 2.25)
100.00
NOTE: Weights are from random effects analysis
.294
1
3.4

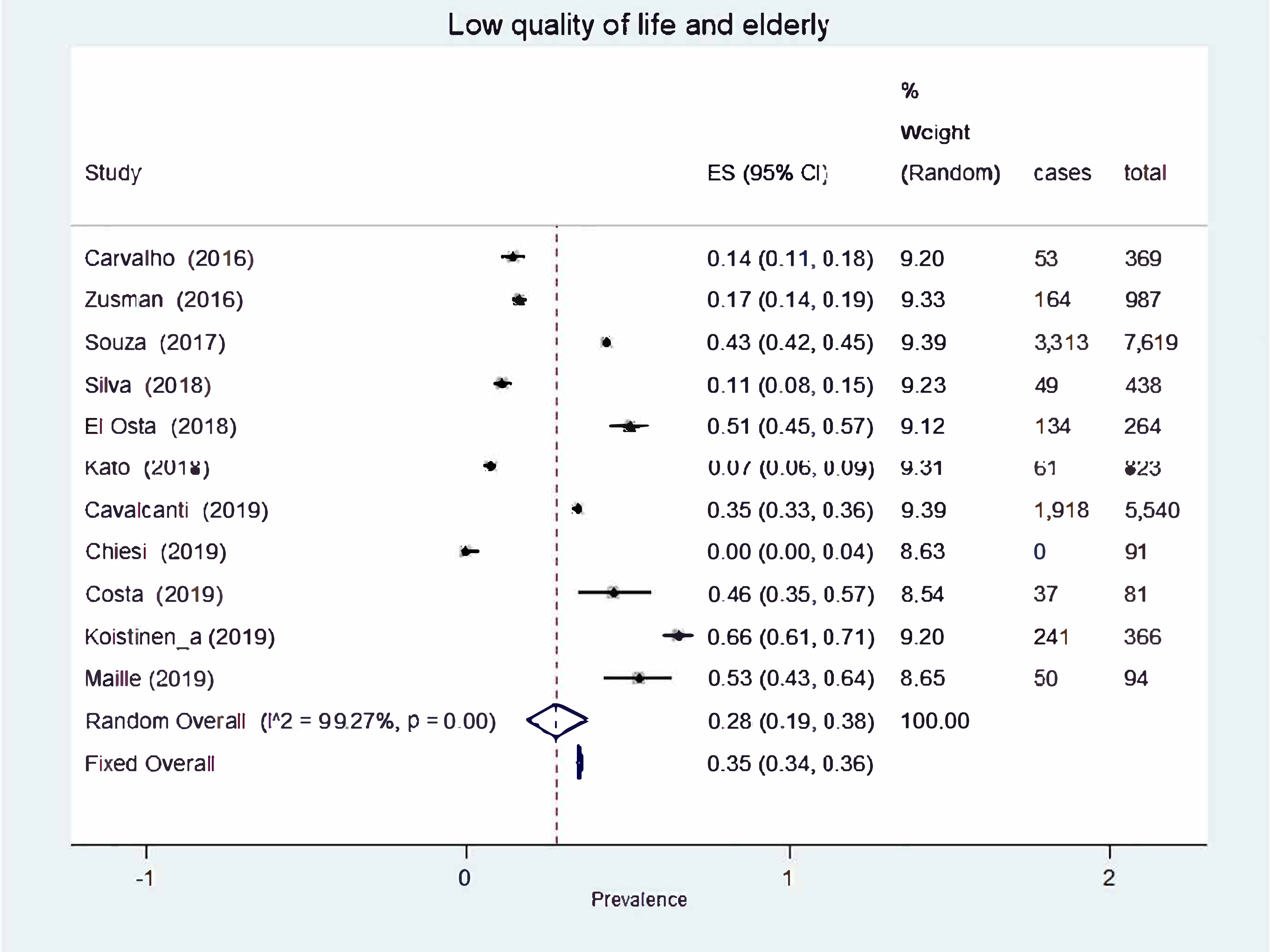
Low quality of life and elderly
Study
ES (95% CI)
% Weight (Random)
cases
total
Carvalho (2016) 0.14 (0.11, 0.18) 9.20 53 369
Zusman (2016) 0.17 (0.14, 0.19) 9.33 164 987
Souza (2017) 0.43 (0.42, 0.45) 9.39 3,313 7,619
Silva (2018) 0.11 (0.08, 0.15) 9.23 49 438
El Osta (2018) 0.51 (0.45, 0.57) 9.12 134 264
Kato (2018) 0.07 (0.06, 0.09) 9.31 61 823
Cavalcanti (2019) 0.35 (0.33, 0.36) 9.39 1,918 5,540
Chiesi (2019) 0.00 (0.00, 0.04) 8.63 0 91
Costa (2019) 0.46 (0.35, 0.57) 8.54 37 81
Koistinen_a (2019) 0.66 (0.61, 0.71) 9.20 241 366
Maille (2019) 0.53 (0.43, 0.64) 8.65 50 94
Random Overall (I^2 = 99.27%, p = 0.00) 0.28 (0.19, 0.38) 100.00
Fixed Overall 0.35 (0.34, 0.36)
-1
0
1
2
Prevalence

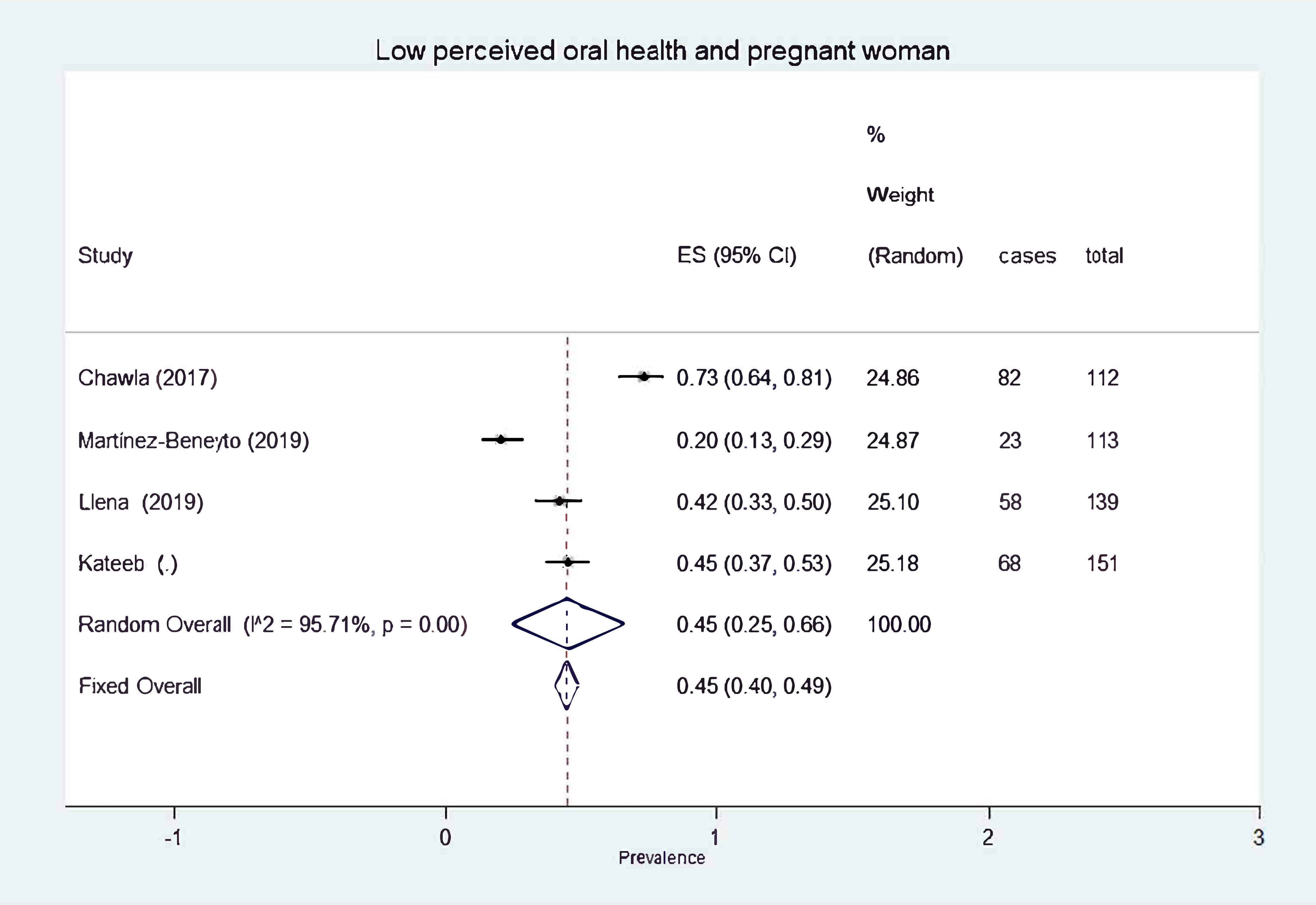
Low perceived oral health and pregnant woman
%
Weight
Study
ES (95% CI)
(Random)
cases
total
Chawla (2017)
0.73 (0.64, 0.81)
24.86
82
112
Martínez-Beneyto (2019)
0.20 (0.13, 0.29)
24.87
23
113
Llena (2019)
0.42 (0.33, 0.50)
25.10
58
139
Kateeb (.)
0.45 (0.37, 0.53)
25.18
68
151
Random Overall (I^2 = 95.71%, p = 0.00)
0.45 (0.25, 0.66)
100.00
Fixed Overall
0.45 (0.40, 0.49)
-1
0
1
2
3
Prevalence

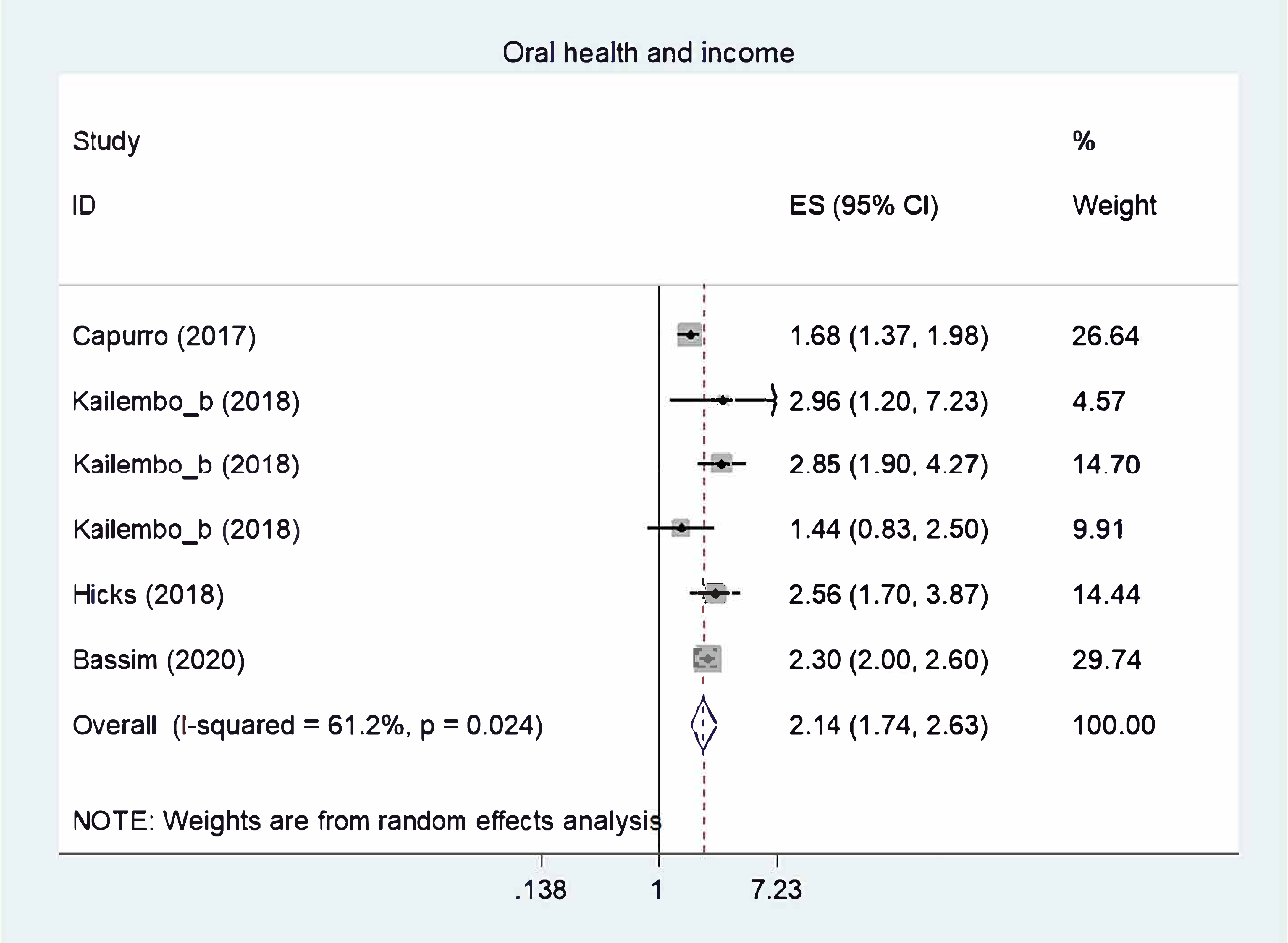
Oral health and income
Study
ID
ES (95% CI)
%
Weight
Capurro (2017)
1.68 (1.37, 1.98)
26.64
Kailembo_b (2018)
2.96 (1.20, 7.23)
4.57
Kailembo_b (2018)
2.85 (1.90, 4.27)
14.70
Kailembo_b (2018)
1.44 (0.83, 2.50)
9.91
Hicks (2018)
2.56 (1.70, 3.87)
14.44
Bassim (2020)
2.30 (2.00, 2.60)
29.74
Overall (I-squared = 61.2%, p = 0.024)
2.14 (1.74, 2.63)
100.00
NOTE: Weights are from random effects analysis
.138
1
7.23

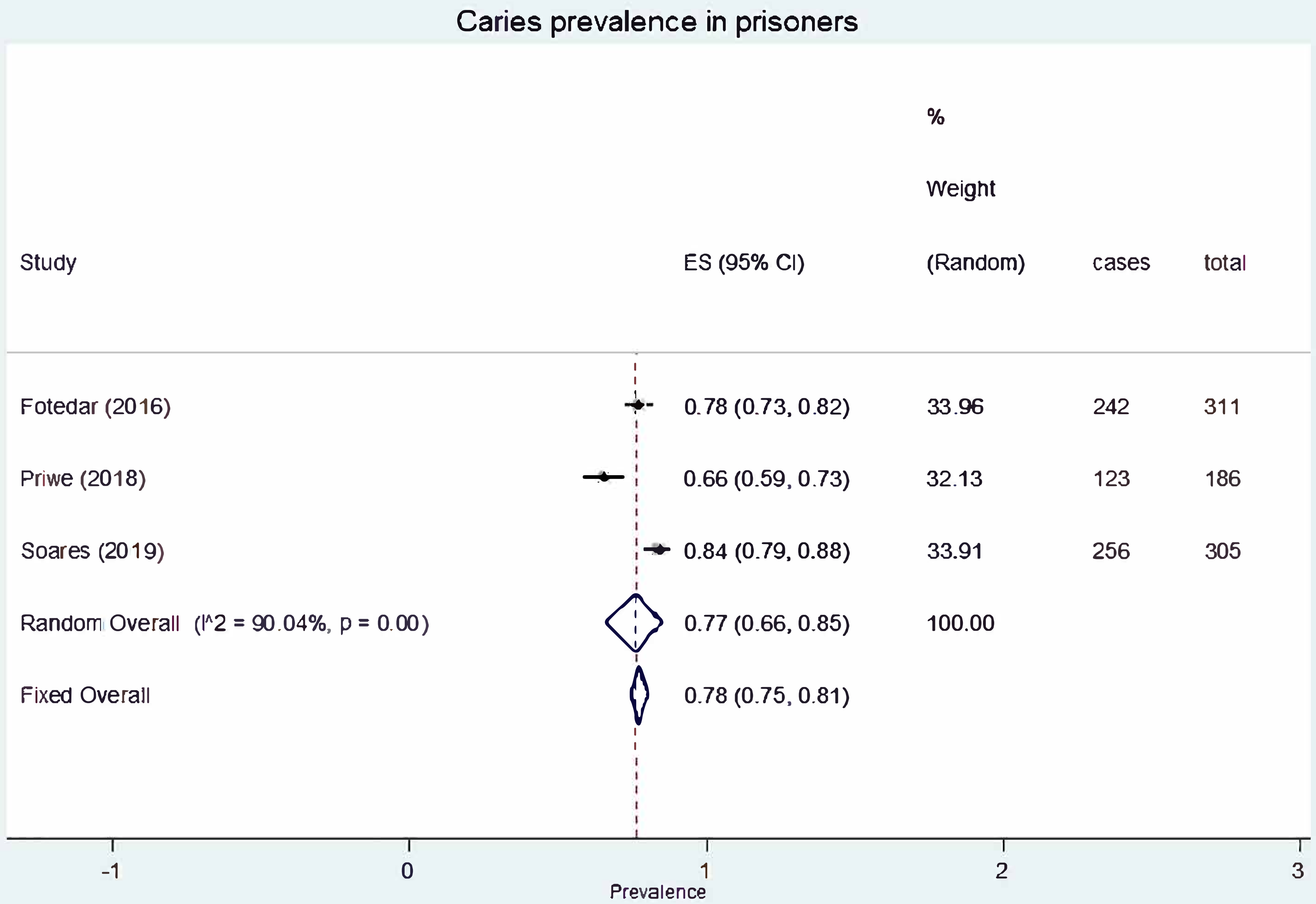
Caries prevalence in prisoners
Study
ES (95% CI)
% Weight (Random)
cases
total
Fotedar (2016)
0.78 (0.73, 0.82)
33.96
242
311
Priwe (2018)
0.66 (0.59, 0.73)
32.13
123
186
Soares (2019)
0.84 (0.79, 0.88)
33.91
256
305
Random Overall (I^2 = 90.04%, p = 0.00)
0.77 (0.66, 0.85)
100.00
Fixed Overall
0.78 (0.75, 0.81)
-1
0
1
2
3
Prevalence

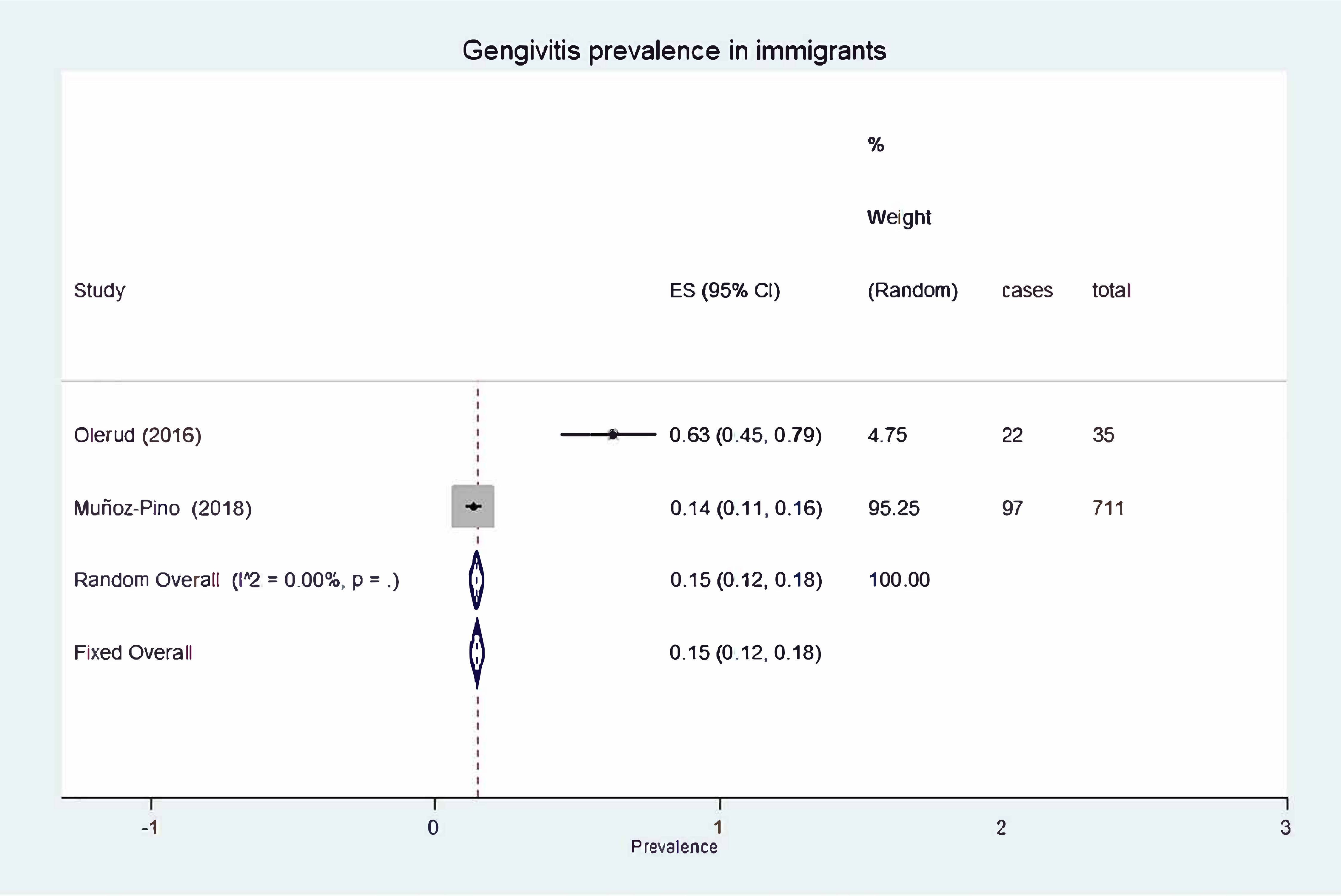

Gengivitis prevalence in immigrants
%
Weight
Study
ES (95% CI)
(Random)
cases
total
Olerud (2016)
0.63 (0.45, 0.79)
4.75
22
35
Muñoz-Pino (2018)
0.14 (0.11, 0.16)
95.25
97
711
Random Overall (I^2 = 0.00%, p = .)
0.15 (0.12, 0.18)
100.00
Fixed Overall
0.15 (0.12, 0.18)
-1
0
1
2
3
Prevalence

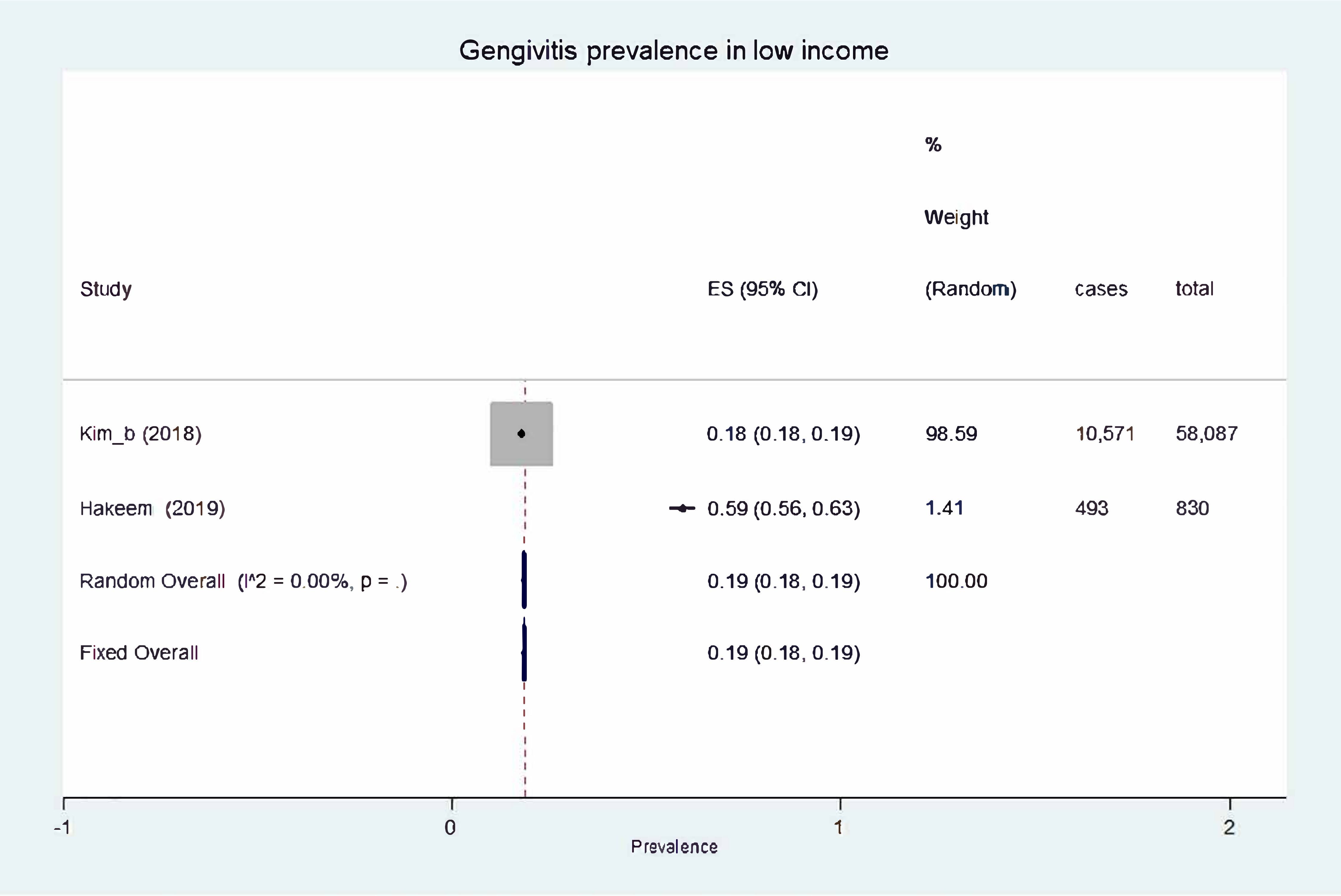

Gengivitis prevalence in low income
%
Weight
Study
ES (95% CI)
(Random)
cases
total
Kim_b (2018)
0.18 (0.18, 0.19)
98.59
10,571
58,087
Hakeem (2019)
0.59 (0.56, 0.63)
1.41
493
830
Random Overall (I^2 = 0.00%, p = .)
0.19 (0.18, 0.19)
100.00
Fixed Overall
0.19 (0.18, 0.19)
-1
0
1
2
Prevalence

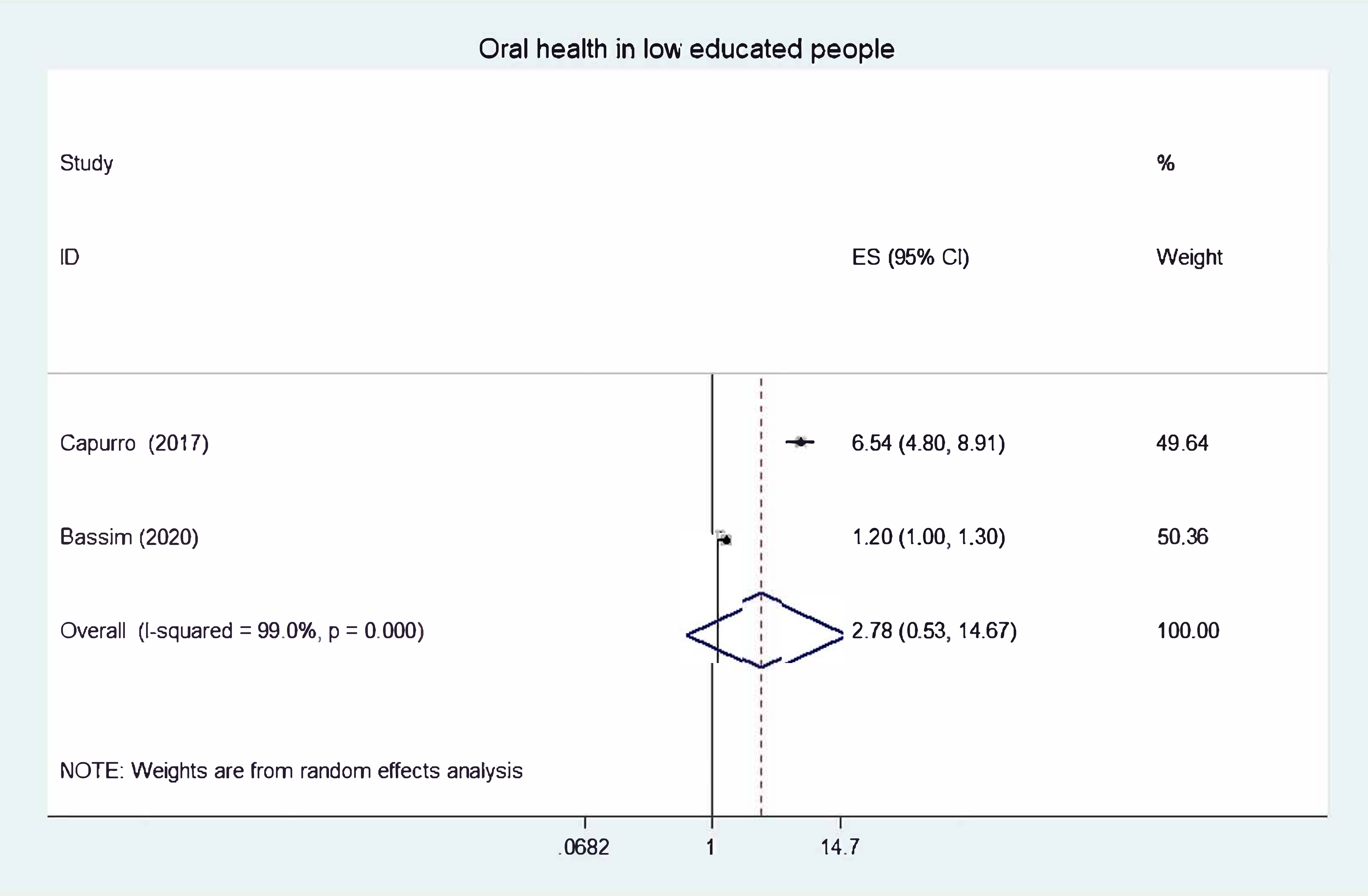

Oral health in low educated people
Study
ID
ES (95% CI)
%
Weight
Capurro (2017)
6.54 (4.80, 8.91)
49.64
Bassim (2020)
1.20 (1.00, 1.30)
50.36
Overall (I-squared = 99.0%, p = 0.000)
2.78 (0.53, 14.67)
100.00
NOTE: Weights are from random effects analysis
.0682
1
14.7

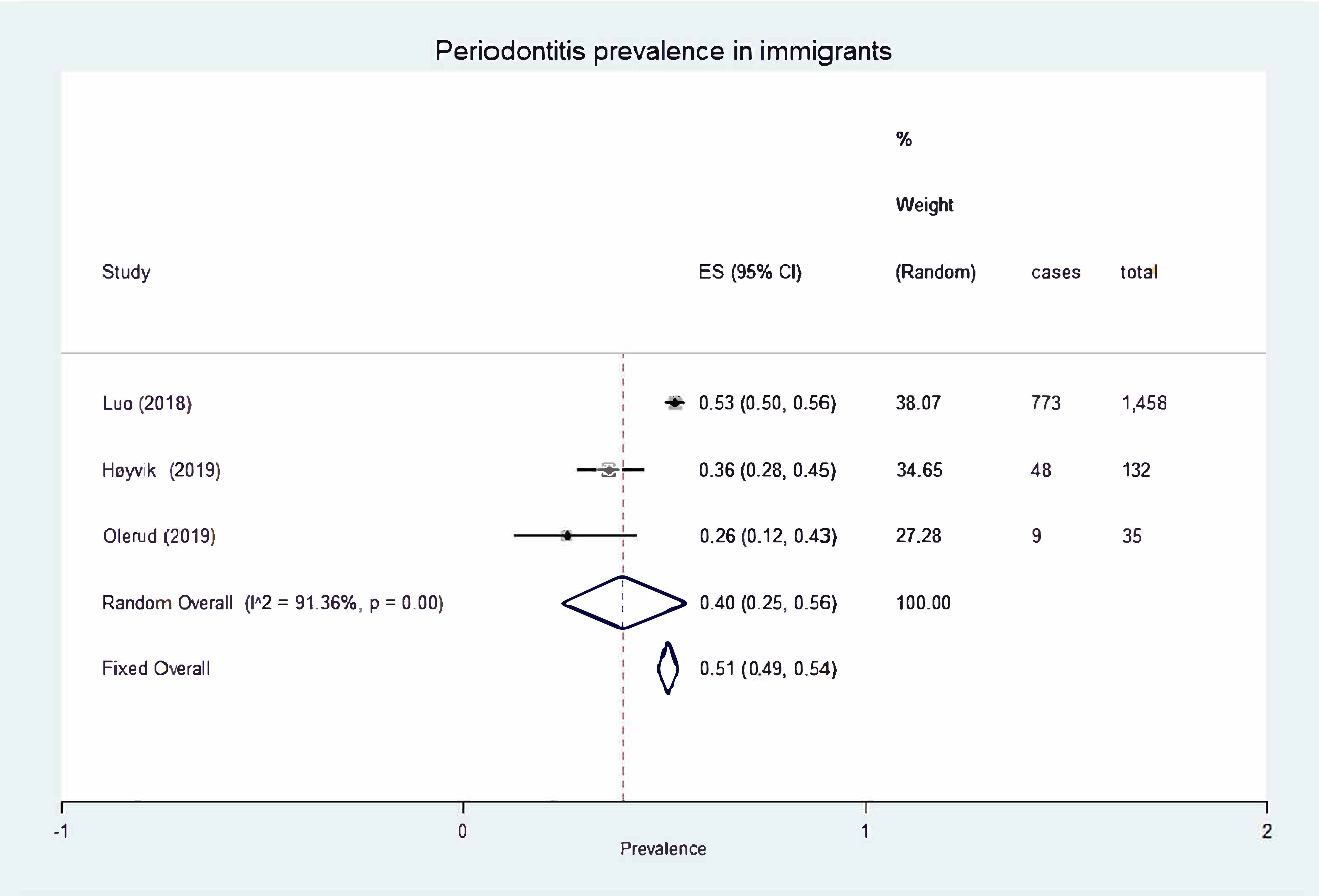
Periodontitis prevalence in immigrants
%
Weight
Study
ES (95% CI)
(Random)
cases
total
Luo (2018)
0.53 (0.50, 0.56)
38.07
773
1,458
Høyvik (2019)
0.36 (0.28, 0.45)
34.65
48
132
Olerud (2019)
0.26 (0.12, 0.43)
27.28
9
35
Random Overall (I^2 = 91.36%, p = 0.00)
0.40 (0.25, 0.56)
100.00
Fixed Overall
0.51 (0.49, 0.54)
-1
0
1
2
Prevalence

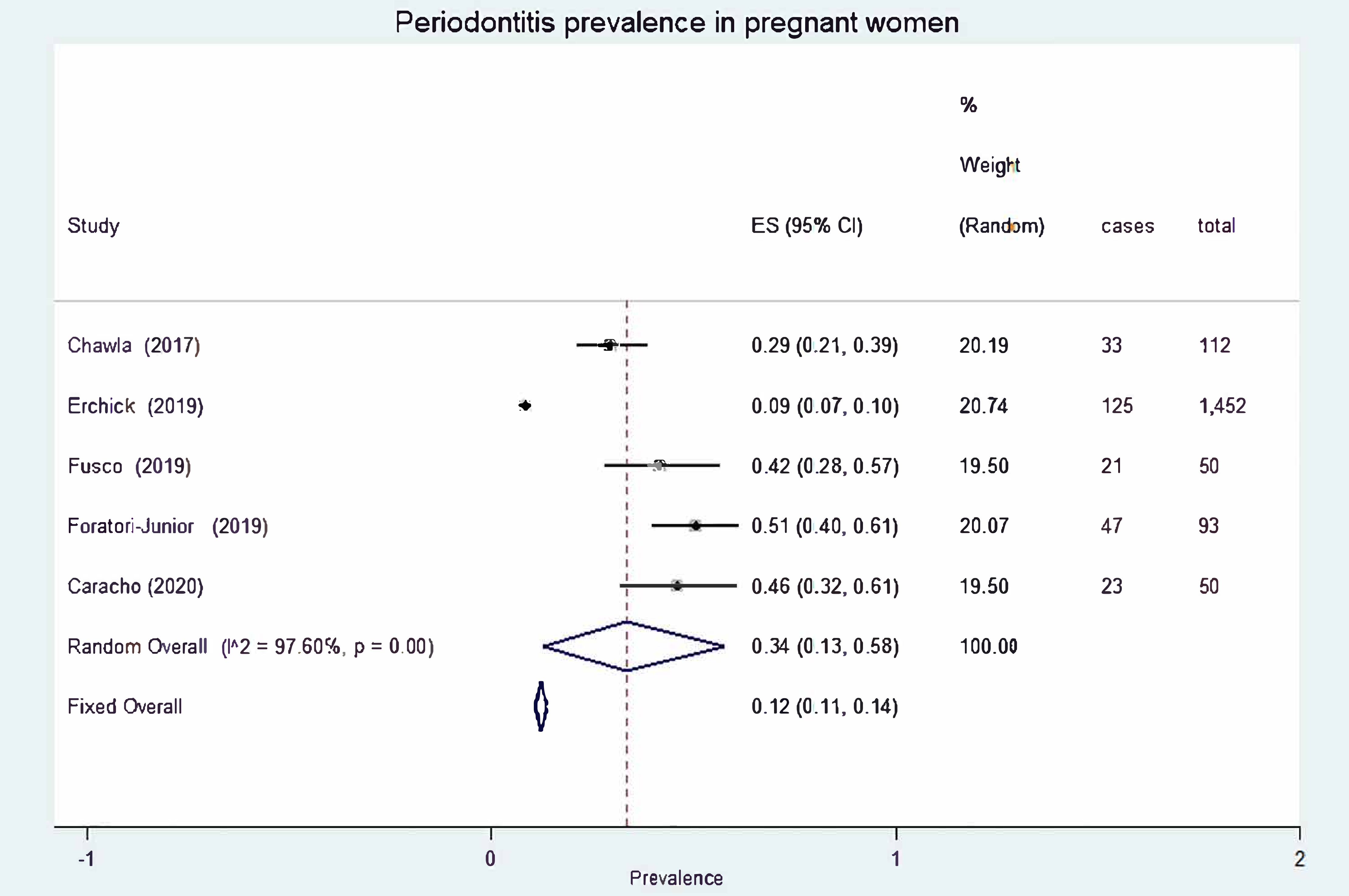

Periodontitis prevalence in pregnant women
%
Weight
Study
ES (95% CI)
(Random)
cases
total
Chawla (2017)
0.29 (0.21, 0.39)
20.19
33
112
Erchick (2019)
0.09 (0.07, 0.10)
20.74
125
1,452
Fusco (2019)
0.42 (0.28, 0.57)
19.50
21
50
Foratori-Junior (2019)
0.51 (0.40, 0.61)
20.07
47
93
Caracho (2020)
0.46 (0.32, 0.61)
19.50
23
50
Random Overall (I^2 = 97.60%, p = 0.00)
0.34 (0.13, 0.58)
100.00
Fixed Overall
0.12 (0.11, 0.14)
-1
0
1
2
Prevalence

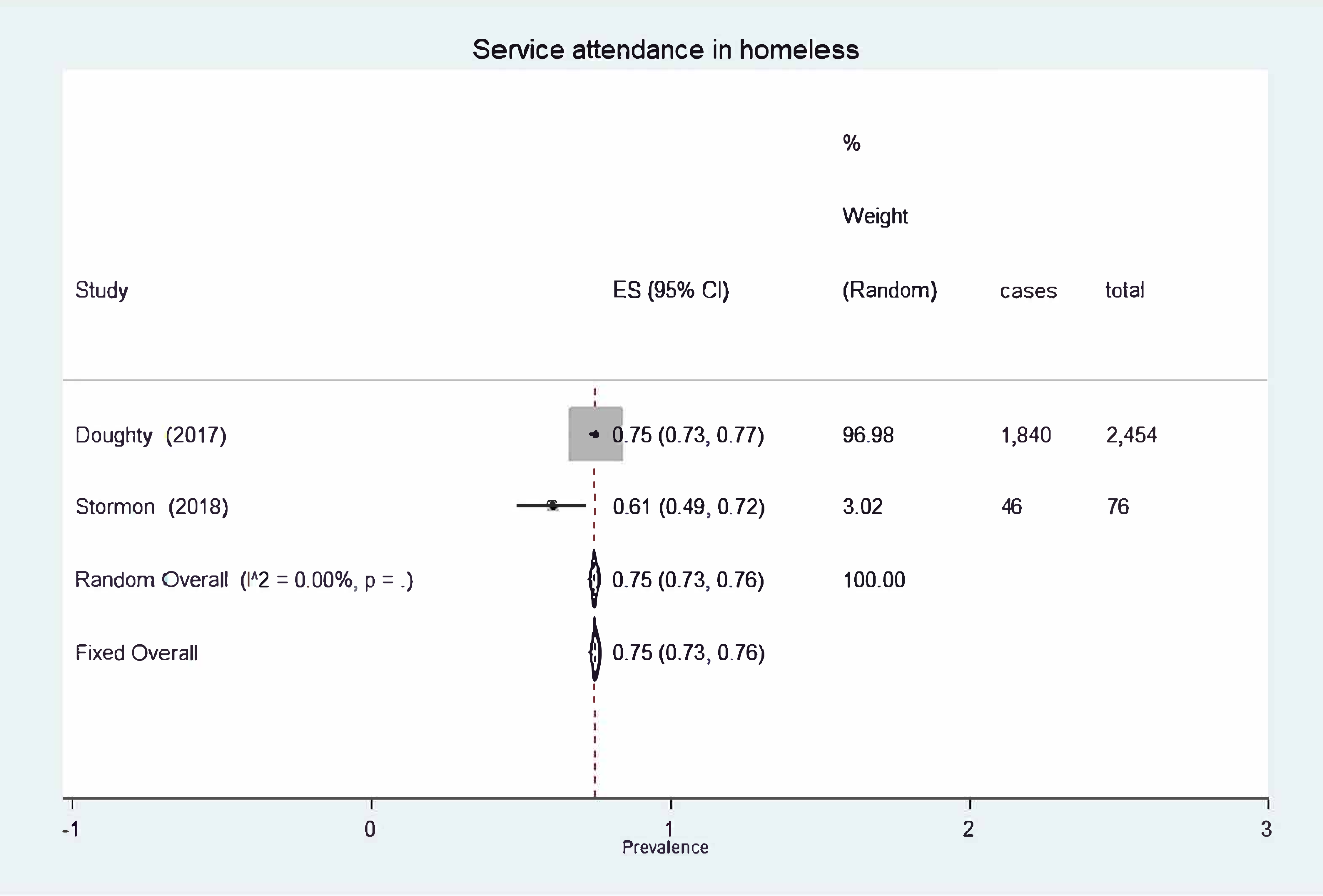
Service attendance in homeless
%
Weight
Study
ES (95% CI)
(Random)
cases
total
Doughty (2017)
0.75 (0.73, 0.77)
96.98
1,840
2,454
Stormon (2018)
0.61 (0.49, 0.72)
3.02
46
76
Random Overall (I^2 = 0.00%, p = .)
0.75 (0.73, 0.76)
100.00
Fixed Overall
0.75 (0.73, 0.76)
-1
0
1
2
3
Prevalence
