# Supplementary material for: Systematic Review of the Literature on Dental Caries and Periodontal Disease in Socio-Economically Disadvantaged Individuals
Source: Int J Environ Res Public Health. 2021 Nov 24;18(23):12360. doi: 10.3390/ijerph182312360 (PMC8656978; doi:10.3390/ijerph182312360)
Supplement: Supplementary file 1 [file ijerph-18-12360-s001.zip › Supplementary Table S1.pdf]

Strategy Search

	**Pubmed (30/11/2020)**
Search	Query
#1	(vulnerable OR vulnerability OR vulnerabilities OR disadvantage OR disadvantaged) AND (("2015/11/30"[Date - Publication] : "2020/11/30"[Date - Publication]))
#2	((("frail elderly"[MeSH Terms]) OR ("aged, 80 and over"[MeSH Terms]) OR ("senior centers"[MeSH Terms]) OR ("homes for the aged"[MeSH Terms]) OR ("homebound persons"[MeSH Terms]) OR ("crime victims"[MeSH Terms]) OR ("pregnant women"[MeSH Terms]) OR ("prisoners"[MeSH Terms]) OR ("refugees"[MeSH Terms]) OR ("refugee camps"[MeSH Terms]) OR ("emigrants and immigrants"[MeSH Terms]) OR ("homeless persons"[MeSH Terms]) OR ("health services for transgender persons"[MeSH Terms]) OR ("sexual and gender minorities"[MeSH Terms]) OR ("sex workers"[MeSH Terms]) OR ("drug users"[MeSH Terms]) OR ("substance related disorders"[MeSH Terms]) OR ("medical indigency"[MeSH Terms]) OR ("medically uninsured"[MeSH Terms]) OR ("socioeconomic factors"[MeSH Terms]) OR ("social problems"[MeSH Terms]) OR ("single parent family"[MeSH Terms]) OR ("working poor"[MeSH Terms]))) AND (("2015/11/30"[Date - Publication] : "2020/11/30"[Date - Publication]))
#3	((dental caries OR periodontal disease OR "oral health" OR "Oral Health"[Mesh] OR tooth loss)) AND (("2015/11/30"[Date - Publication] : "2020/11/30"[Date - Publication]))
#4	(#1 OR #2) AND #3
#5	(#1 OR #2) AND #3Filters: Humans, English, Italian, Adult: 19+ years

	**Embase (30/11/2020)**
Search	Query
#1	(vulnerable OR vulnerability OR vulnerabilities OR disadvantage OR disadvantaged) AND [30-11-2015]/sd NOT [1-12-2020]/sd
#2	('frail elderly' OR 'very elderly' OR 'senior center' OR 'home for the aged' OR 'homebound patient' OR 'crime victim' OR 'pregnant woman' OR 'prisoner' OR 'refugee' OR 'refugee camp' OR 'migrant' OR 'homeless person' OR 'transgender' OR 'sexual and gender minority' OR 'sex worker' OR 'drug use' OR 'drug dependence' OR 'medical indigency' OR 'medically uninsured' OR 'socioeconomics' OR 'social problem' OR 'single-parent family' OR 'working poor') AND [30-11-2015]/sd NOT [1-12-2020]/sd
#3	#1 OR #2
#4	('dental caries' OR 'tooth loss' OR 'periodontal disease' OR 'oral health') AND [30-11-2015]/sd NOT [1-12-2020]/sd
#5	#3 AND #4
#6	#5 AND 'human'/de AND ('article'/it OR 'article in press'/it OR 'review'/it) AND ([adult]/lim OR [aged]/lim) AND ([english]/lim OR [italian]/lim)

	**Web of Science (30/11/2020)**
Search	Query

#1	(TS=(vulnerable OR vulnerability OR vulnerabilities OR disadvantage OR disadvantaged))AND LANGUAGE: (English OR Italian) AND DOCUMENT TYPES: (Article OR Review)<br>Indexes=SCI-EXPANDED, SSCI, A&HCI, CPCI-S, CPCI-SSH, ESCI Timespan=2016-2020
#2	(TS=((("frail elderly") OR ("very aged") OR ("senior center*") OR (homes AND aged) OR ("homebound person*") OR ("crime victim*") OR ("pregnant women" OR "pregnant woman") OR ("prisoner*") OR ("refugee*") OR ("refugee camp*") OR (emigrant* OR immigrant* OR migrant*) OR ("homeless person*") OR (health services AND transgender*) OR ((sexual OR gender) AND minorit*) OR ("sex worker*") OR ("drug user*") OR ("substance related disorder*") OR ("medical indigenc*") OR ("medically uninsured") OR ("socioeconomic factor*") OR ("social problem*") OR ("single parent famil*") OR ("working poor")))) AND LANGUAGE: (English) AND DOCUMENT TYPES: (Article)<br>Indexes=SCI-EXPANDED, SSCI, A&HCI, CPCI-S, CPCI-SSH, ESCI Timespan=2016-2020
#3	(TS=("dental caries" OR "tooth loss" OR "periodontal disease*" OR "oral health")) AND LANGUAGE: (English) AND DOCUMENT TYPES: (Article)<br>Indexes=SCI-EXPANDED, SSCI, A&HCI, CPCI-S, CPCI-SSH, ESCI Timespan=2016-2020
#4	(#1 OR #2) AND #3
