# Supplementary material for: Systematic Review of the Literature on Dental Caries and Periodontal Disease in Socio-Economically Disadvantaged Individuals
Source: Int J Environ Res Public Health. 2021 Nov 24;18(23):12360. doi: 10.3390/ijerph182312360 (PMC8656978; doi:10.3390/ijerph182312360)
Supplement: Supplementary file 1 [file ijerph-18-12360-s001.zip › Supplementary Table S2.pdf]

Quality assessment

Cross-sectional studies

Author/s and years	Item 1: Inclusion criteria definition	Item 2: Subjects and the setting description	Item 3: Exposure measurements	Item 4: Standard criteria for measurements	Item 5: Confounding factors identification	Item 6: Strategies to deal with confounding factors	Item 7: Outcomes measurements	Item 8: Statistical analysis
Abu-Awwad 2019	yes	yes	yes	yes	no	no	yes	yes
Adesina 2018	yes	yes	yes	yes	no	no	yes	yes
Aida 2016	yes	yes	yes	yes	no	no	yes	yes
Akintobi 2018	yes	yes	yes	yes	yes	yes	yes	yes
Álvarez 2015	yes	yes	yes	yes	no	no	yes	yes
Anderson 2016	yes	yes	yes	yes	no	no	yes	yes
Andersson 2017	yes	yes	yes	yes	no	no	yes	yes
Andrade 2019	yes	yes	yes	yes	no	no	yes	yes
Antoniazzi 2016	yes	yes	yes	yes	yes	yes	yes	yes
Arrica 2017	yes	yes	yes	yes	yes	yes	yes	yes
Aukštakalnis 2018	yes	yes	yes	yes	no	no	yes	yes
Bachkati 2016	yes	yes	yes	yes	no	no	yes	yes
Bakker 2018	yes	yes	yes	yes	no	no	yes	yes
Bakker 2020	yes	yes	yes	yes	no	no	yes	yes
Bassim 2020	yes	yes	yes	yes	yes	yes	yes	yes
Berglund 2017	yes	yes	yes	yes	yes	yes	yes	yes
Bhat 2017	yes	yes	yes	yes	yes	yes	yes	yes
Blasi 2018	yes	yes	yes	yes	yes	yes	yes	yes
Bomfim 2018	yes	yes	yes	yes	no	no	yes	yes
Caldwell 2017	yes	yes	yes	yes	yes	yes	yes	yes
Calvasina 2016	yes	yes	yes	yes	yes	yes	yes	yes
Capurro 2017	yes	yes	yes	yes	yes	yes	yes	yes
Caracho 2020	yes	yes	yes	yes	no	no	yes	yes
Carvalho 2016	yes	yes	yes	yes	no	no	yes	yes
Carvalho 2020	yes	yes	yes	yes	no	no	yes	yes
Castrejón-Pérez 2017b	yes	yes	yes	yes	no	no	yes	yes
Celeste 2019	yes	yes	yes	yes	yes	yes	yes	yes

Chae 2017	yes	yes	yes	yes	no	no	yes	yes
Chahar 2019	yes	yes	yes	yes	no	no	yes	yes
Chalub 2016	yes	yes	yes	yes	yes	yes	yes	yes
Chawla 2017	yes	yes	yes	yes	no	no	yes	yes
Chen 2018	yes	yes	yes	yes	no	no	yes	yes
Chiesi 2019	yes	yes	yes	yes	no	no	yes	yes
Chiu 2017	yes	yes	yes	yes	no	no	yes	yes
Choi 2017	yes	yes	yes	yes	yes	yes	yes	yes
Clague 2017	yes	yes	yes	yes	no	no	yes	yes
Cocco 2018	yes	yes	yes	yes	no	no	yes	yes
Costa 2019	yes	yes	yes	yes	no	no	yes	yes
da Veiga Pessoa 2016	yes	yes	yes	yes	no	no	yes	yes
Dahl 2018	yes	yes	yes	yes	no	no	yes	yes
De Visschere 2016	yes	yes	yes	yes	no	no	yes	yes
De Visschere 2016	yes	yes	yes	yes	no	no	yes	yes
Delwel 2018	yes	yes	yes	yes	no	no	yes	yes
Doughty 2018	yes	yes	yes	yes	no	no	yes	yes
Duijster 2018	yes	yes	yes	yes	yes	yes	yes	yes
El Osta 2018	yes	yes	yes	yes	no	no	yes	yes
Elkerdany 2020	yes	yes	yes	yes	no	no	yes	yes
Erchick 2019	yes	yes	yes	yes	no	no	yes	yes
Fantin 2017	yes	yes	yes	yes	no	no	yes	yes
Fotedar 2016	yes	yes	yes	yes	no	no	yes	yes
Franciscatto 2020	yes	yes	yes	yes	yes	yes	yes	yes
Furuta 2018	yes	yes	yes	yes	no	no	yes	yes
Gao 2018a	yes	yes	yes	yes	no	no	yes	yes
Głowacka 2019	yes	yes	yes	yes	no	no	yes	yes
Gomes Filho 2019	yes	yes	yes	yes	yes	yes	yes	yes
Goulart Mde 2016	yes	yes	yes	yes	yes	yes	yes	yes
Griffin 2019	yes	yes	yes	yes	no	no	yes	yes
Grover 2016	yes	yes	yes	yes	yes	yes	yes	yes
Hach 2019	yes	yes	yes	yes	yes	yes	yes	yes

Hakeem 2019	yes	yes	yes	yes	yes	yes	yes	yes
Han 2015	yes	yes	yes	yes	no	no	yes	yes
Heredia-Ponce 2017	yes	yes	yes	yes	no	no	yes	yes
Hicks 2018	yes	yes	yes	yes	yes	yes	yes	yes
Hoeksema 2018	yes	yes	yes	yes	no	no	yes	yes
Hoeksema 2017	yes	yes	yes	yes	no	no	yes	yes
Hoeksema 2018	yes	yes	yes	yes	no	no	yes	yes
Høyvik 2019	yes	yes	yes	yes	no	no	yes	yes
Ishikawa 2019	yes	yes	yes	yes	yes	yes	yes	yes
Janssens 2017	yes	yes	yes	yes	no	no	yes	yes
Janssens 2017	yes	yes	yes	yes	no	no	yes	yes
Janssens 2018	yes	yes	yes	yes	no	no	yes	yes
Kailembo 2018	yes	yes	yes	yes	yes	yes	yes	yes
Kateeb 2018	yes	yes	yes	yes	no	no	yes	yes
Kato 2018	yes	yes	yes	yes	no	no	yes	yes
Kim 2016	yes	yes	yes	yes	yes	yes	yes	yes
Kim 2018	yes	yes	yes	yes	no	no	yes	yes
Klotz 2020	yes	yes	yes	yes	no	no	yes	yes
Laguzzi 2016	yes	yes	yes	yes	no	no	yes	yes
Lambert 2018	yes	yes	yes	yes	yes	yes	yes	yes
Lee 2016	yes	yes	yes	yes	yes	yes	yes	yes
Lee 2018	yes	yes	yes	yes	no	no	yes	yes
León 2016	yes	yes	yes	yes	no	no	yes	yes
Llena 2019	yes	yes	yes	yes	no	no	yes	yes
Maille 2017	yes	yes	yes	yes	no	no	yes	yes
Maille 2019	yes	yes	yes	yes	no	no	yes	yes
Marques 2015	yes	yes	yes	yes	no	no	yes	yes
Martin 2017	yes	yes	yes	yes	no	no	yes	yes
Matsuyama 2019	yes	yes	yes	yes	no	no	yes	yes
Mehr 2018	yes	yes	yes	yes	no	no	yes	yes
Mejia 2018	yes	yes	yes	yes	no	no	yes	yes
Mochida 2018	yes	yes	yes	yes	no	no	yes	yes

Monaghan 2017	yes	yes	yes	yes	no	no	yes	yes
Mulic 2020	yes	yes	yes	yes	no	no	yes	yes
Muñoz-Pino 2018	yes	yes	yes	yes	no	no	yes	yes
Nakahori 2019	yes	yes	yes	yes	no	no	yes	yes
Naorungroj 2017	yes	yes	yes	yes	no	no	yes	yes
Niesten 2016	yes	yes	yes	yes	no	no	yes	yes
Niesten 2017	yes	yes	yes	yes	no	no	yes	yes
Nihtilä 2017	yes	yes	yes	yes	no	no	yes	yes
Nilsson 2018	yes	yes	yes	yes	no	no	yes	yes
Nogueira 2019	yes	yes	yes	yes	yes	yes	yes	yes
Nouaman 2015	yes	yes	yes	yes	no	no	yes	yes
Novrinda 2019	yes	yes	yes	yes	no	no	yes	yes
Okamoto 2019	Yes	yes	yes	yes	no	no	yes	yes
Olerud 2016	Yes	yes	yes	yes	no	no	yes	yes
Olerud 2016	Yes	yes	yes	yes	no	no	yes	yes
Piotrowska 2020	Yes	yes	yes	yes	yes	yes	yes	yes
Priwe 2018	Yes	yes	yes	yes	no	no	yes	yes
Puturidze 2019	Yes	yes	yes	yes	no	no	yes	yes
Rafat 2020	Yes	yes	yes	yes	no	no	yes	yes
Ramsay 2015	Yes	yes	yes	yes	no	no	yes	yes
Ramsay 2018	Yes	yes	yes	yes	yes	yes	yes	yes
Rao 2017	Yes	yes	yes	yes	no	no	yes	yes
Rekhi 2016	Yes	yes	yes	yes	no	no	yes	yes
Rekhi 2018	Yes	yes	yes	yes	no	no	yes	yes
Ribeiro 2016	Yes	yes	yes	yes	no	no	yes	yes
Roberto 2020	Yes	yes	yes	yes	yes	yes	yes	yes
Rodriguez 2017	Yes	yes	yes	yes	yes	yes	yes	yes
Rommel 2016	Yes	yes	yes	yes	no	no	yes	yes
Rosa 2020	Yes	yes	yes	yes	no	no	yes	yes
Rosa 2020	Yes	yes	yes	yes	no	no	yes	yes
Rota 2019	Yes	yes	yes	yes	no	no	yes	yes
Rouxel 2015	Yes	yes	yes	yes	yes	yes	yes	yes

Sáez-Prado 2016	Yes	yes	yes	yes	no	no	yes	yes
Saintrain 2018	Yes	yes	yes	yes	no	no	yes	yes
Saito 2019	Yes	yes	yes	yes	no	no	yes	yes
Sato 2018	Yes	yes	yes	yes	yes	yes	yes	yes
Schwindling 2018	Yes	yes	yes	yes	no	no	yes	yes
Sheng 2018	Yes	yes	yes	yes	no	no	yes	yes
Shetty 2015	Yes	yes	yes	yes	yes	yes	yes	yes
Shim 2018	Yes	yes	yes	yes	yes	yes	yes	yes
Silva 2018	Yes	yes	yes	yes	no	no	yes	yes
Singh 2016	Yes	yes	yes	yes	no	no	yes	yes
Singh 2016	yes	yes	yes	yes	no	no	yes	yes
Skośkiewicz-Malinowska 2016	yes	yes	yes	yes	no	no	yes	yes
Soares 2019	yes	yes	yes	yes	no	no	yes	yes
Song 2016	yes	yes	yes	yes	no	no	yes	yes
Sordi 2017	yes	yes	yes	yes	no	no	yes	yes
Souza 2018	yes	yes	yes	yes	no	no	yes	yes
Srisilapanan 2016	yes	yes	yes	yes	no	no	yes	yes
Srivastava 2017	yes	yes	yes	yes	yes	yes	yes	yes
Steele 2015	yes	yes	yes	yes	no	no	yes	yes
Stormon 2018	yes	yes	yes	yes	no	no	yes	yes
Sun 2016	yes	yes	yes	yes	no	no	yes	yes
Suzuki 2016	yes	yes	yes	yes	yes	yes	yes	yes
Tafere 2018	yes	yes	yes	yes	yes	yes	yes	yes
Tapias Perdigon 2019	yes	yes	yes	yes	no	no	yes	yes
Tiwari 2016	yes	yes	yes	yes	no	no	yes	yes
Umer 2016	yes	yes	yes	yes	no	no	yes	yes
Valdez 2020	yes	yes	yes	yes	no	no	yes	yes
Wang 2017	yes	yes	yes	yes	no	no	yes	yes
Wang 2017	yes	yes	yes	yes	no	no	yes	yes
Weintraub 2019	yes	yes	yes	yes	yes	yes	yes	yes
Wennström 2015	yes	yes	yes	yes	no	no	yes	yes
Wilson 2018	yes	yes	yes	yes	yes	yes	yes	yes

Wright 2017	yes	yes	yes	yes	no	no	yes	yes
Wright 2018	yes	yes	yes	yes	no	no	yes	yes
Xhihani 2017	yes	yes	yes	yes	no	no	yes	yes
Ye 2018	yes	yes	yes	yes	no	no	yes	yes
Yoon 2018	yes	yes	yes	yes	no	no	yes	yes
Yoshino 2016	yes	yes	yes	yes	yes	yes	yes	yes
Yoshino 2017	yes	yes	yes	yes	yes	yes	yes	yes
Zhang 2016b	yes	yes	yes	yes	no	no	yes	yes
Zhang_a 2016	yes	yes	yes	yes	no	no	yes	yes
Ziebolz 2017	yes	yes	yes	yes	no	no	yes	yes
Zimmerman 2017	yes	yes	yes	yes	no	no	yes	yes
Zusman 2016	yes	yes	yes	yes	no	no	yes	yes

Cohort studies

Author/s and years	Item 1: Population and groups recruitment	Item 2: Exposure similarity measures (exposed/unexposed groups)	Item 3: Exposure measurements	Item 4: Confounding factors identification	Item 5: Strategies to deal with confounding factors	Item 6: No outcome at the moment of exposure	Item 7: Outcomes measurements	Item 8: Appropriate follow-up	Item 9: Follow-up completeness	Item 10: Strategies to address incomplete follow up	Item 11: Statistical analysis
Beker 2019	yes	yes	yes	no	no	yes	yes	yes	yes	yes	yes
Castrejón-Pérez 2017a	yes	yes	yes	no	no	yes	yes	yes	yes	yes	yes
Costa 2016	yes	yes	yes	no	no	yes	yes	yes	yes	yes	yes
DeLia 2016	yes	yes	yes	no	no	yes	yes	yes	yes	yes	yes
Freiberg 2020	yes	yes	yes	no	no	yes	yes	yes	yes	yes	yes
Laniado 2020	yes	yes	yes	no	no	yes	yes	yes	yes	yes	yes
Lee 2019	yes	yes	yes	yes	yes	yes	yes	yes	yes	yes	yes
Rampa 2016	yes	yes	yes	yes	yes	yes	yes	yes	yes	yes	yes
Rio 2020	yes	yes	yes	no	no	yes	yes	yes	yes	yes	yes

Case control studies

Author/s and years	Item 1: Groups comparability other than presence/absence of disease	Item 2: Cases and controls matching	Item 3: Cases/controls criteria identification	Item 4: Exposure measurements	Item 5: Procedure similarity for cases and controls	Item 6: Confounding factors identification	Item 7: Strategies to deal with confounding factors	Item 8: Outcomes assessments	Item 9: Appropriate exposure period	Item 10: Statistical analysis
Gao 2018b	yes	yes	yes	yes	yes	no	no	yes	yes	yes
Gao 2020	yes	yes	yes	yes	yes	no	no	yes	yes	yes
Manicone 2017	yes	yes	yes	yes	yes	no	no	yes	yes	yes
Martínez-Beneyto 2019	yes	yes	yes	yes	yes	no	no	yes	yes	yes
